# The anti-aromatic dianion and aromatic tetraanion of [18]annulene

**DOI:** 10.1038/s41557-024-01469-1

**Published:** 2024-03-06

**Authors:** Wojciech Stawski, Yikun Zhu, Igor Rončević, Zheng Wei, Marina A. Petrukhina, Harry L. Anderson

**Affiliations:** 1 Department of Chemistry, University at Albany, State University of New York, Albany, NY USA; 2 Department of Chemistry, Oxford University, Chemistry Research Laboratory, Oxford, UK

**Keywords:** Structure elucidation, Density functional theory, Chemical bonding

## Abstract

π-Conjugated macrocycles behave differently from analogous linear chains because their electronic wavefunctions resemble a quantum particle on a ring, leading to aromaticity or anti-aromaticity. [18]Annulene, (CH)_18_, is the archetypal non-benzenoid aromatic hydrocarbon. Molecules with circuits of 4*n* + 2 π electrons, such as [18]annulene (*n* = 4), are aromatic, with enhanced stability and diatropic ring currents (magnetic shielding inside the ring), whereas those with 4*n* π electrons, such as the dianion of [18]annulene, are expected to be anti-aromatic and exhibit the opposite behaviour. Here we use ^1^H NMR spectroscopy to re-evaluate the structure of the [18]annulene dianion. We also show that it can be reduced further to an aromatic tetraanion, which has the same shape as the dianion. The crystal structure of the tetraanion lithium salt confirms its geometry and reveals a metallocene-like sandwich, with five Li^+^ cations intercalated between two [18]annulene tetraanions. We also report a heteroleptic sandwich, with [18]annulene and corannulene tetraanion decks.

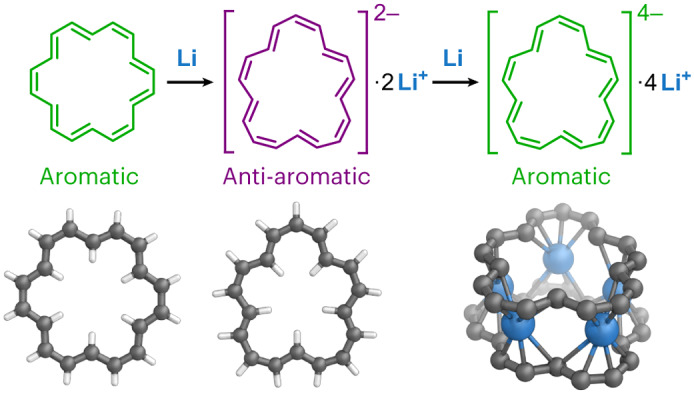

## Abstract (web summary)

A previous investigation of the anti-aromatic dianion of [18]annulene concluded that it consists of a mixture of two isomers. Now it has been shown that this dianion exists as a single isomer, with a different geometry from neutral [18]annulene, and that it can be reduced further to an aromatic tetraanion.

## Main

[18]Annulene, (CH)_18_, is one of the iconic molecules of organic chemistry. Sondheimer’s investigation of this compound in the 1960s provided a compelling endorsement for molecular orbital theory by showing that Hückel’s 4*n* + 2 rule extends to molecules substantially larger than benzene^1–3^. In 1973, Oth, Woo and Sondheimer reported that [18]annulene can be reduced to an anti-aromatic dianion (20 π electrons)^4^. Here we show that their published structural assignment of this dianion was incorrect, and that [18]annulene can also be reduced to a stable aromatic tetraanion (22 π electrons). The ^1^H nuclear magnetic resonance (NMR) spectra of the dianion and tetraanion confirm that they are anti-aromatic and aromatic, respectively, and indicate that [18]annulene adopts a *C*_2v_ conformation in both reduced states that contrasts the virtual *D*_6h_ symmetry of the neutral ring.

**Fig. 1:**
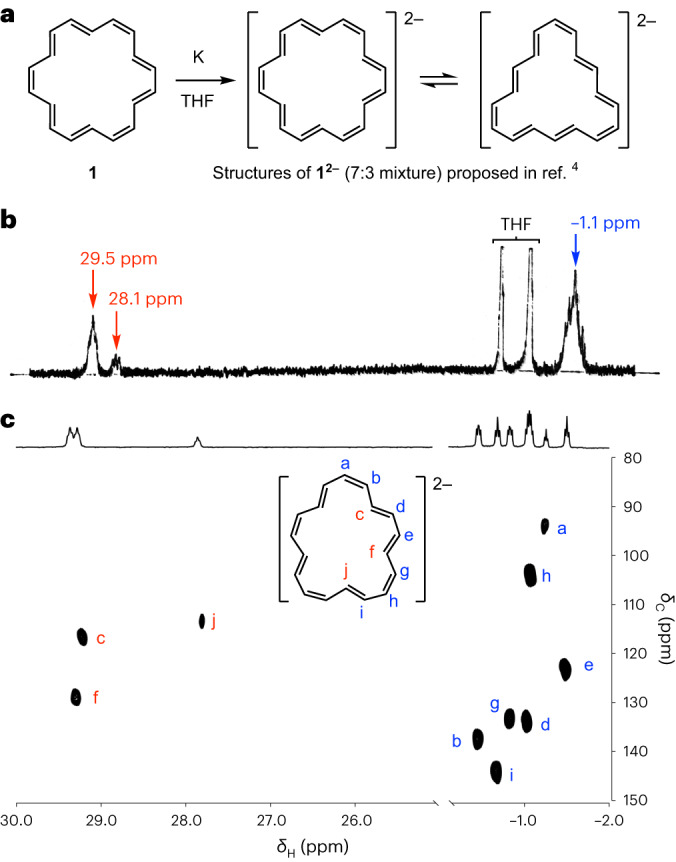
Reduction of [18]annulene with potassium. **a**, Structures of the dianion proposed in ref. ^4^. **b**, ^1^H NMR spectrum reported in ref. ^4^ (THF-*d*_8_, 60 MHz, −110 °C). **c**, ^1^H-^13^C heteronuclear single quantum coherence NMR spectrum (THF-*d*_8_, 500 MHz, −70 °C), and revised structural assignment of the dianion. Resonances a–f were assigned from the correlated spectroscopy (^1^H-^1^H COSY) spectrum (Supplementary Figs. 21 and 22).

Oth et al. reported^4^ that [18]annulene **1** can be reduced with potassium, and that the ^1^H NMR spectrum of the resulting dianion exhibits peaks at 29.5, 28.1 and −1.13 ppm, at −110 °C in THF-*d*_8_. They assigned this spectrum to an interconverting mixture of two isomers of the dianion in a 7:3 ratio (Fig. 1a). However, the published 60 MHz ^1^H NMR spectrum (Fig. 1b) is poorly resolved, so we decided to reinvestigate this dianion.

## Results and discussion

### ^1^H NMR spectroscopy of the dianion and tetraanion

We found that reduction of [18]annulene with potassium metal in THF-*d*_8_ gives a green solution and that the ^1^H NMR spectrum of this solution (recorded at −70 °C, 500 MHz) reveals ten CH multiplets (triplets or double doublets) that are clearly resolved in the ^1^H-^13^C heteronuclear single quantum coherence NMR spectrum (Fig. 1c). Three signals resonate at high chemical shift: 29.47 (2H), 29.39 (2H) and 27.97 (1H) ppm, and seven appear at low chemical shift: −0.50 (2H), −0.73 (2H), −0.87 (2H), −1.11 (2H), −1.08 (2H), −1.29 (1H) and −1.54 (2H) ppm. This pattern of integrals and number of signals indicates that the dianion has twofold symmetry with five protons inside the ring (strongly deshielded) and 13 protons outside the ring (shielded), as shown in our revised structural assignment (Fig. 1c). The signals that we observed have very similar chemical shifts to those reported in ref. ^4^ and it is clear we observe the same dianion salt, **1·K**_**2**_.

**Fig. 2:**
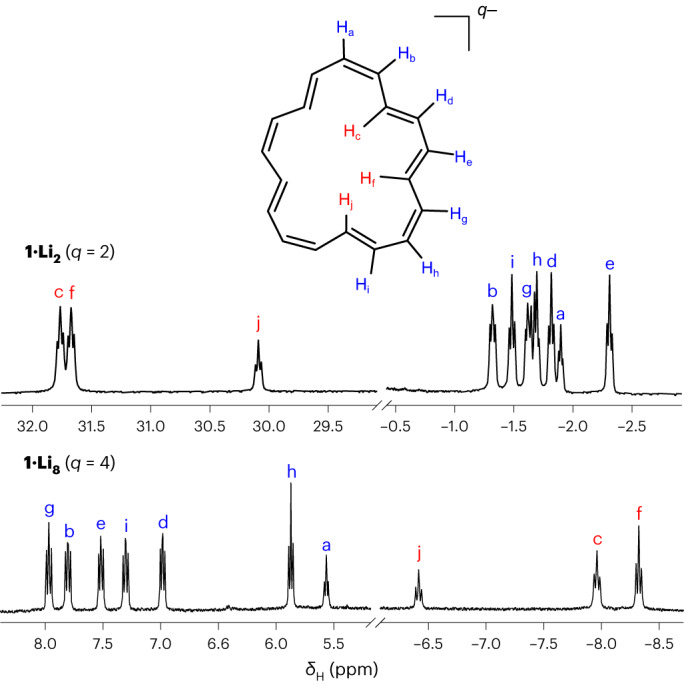
^1^H NMR spectra of the dianion (top) and tetraanion (bottom) with Li^+^ counter ions. THF-*d*_8_; temperatures were −70 °C for **1**^**2−**^ and 25 °C for **1**^**4−**^; 500 MHz. The spectra were fully assigned using ^1^H-^1^H COSY and nuclear Overhauser effect spectroscopy techniques (Supplementary Figs. 32, 33, 38, 39 and 44–46).

**Extended Data Fig. 1:**
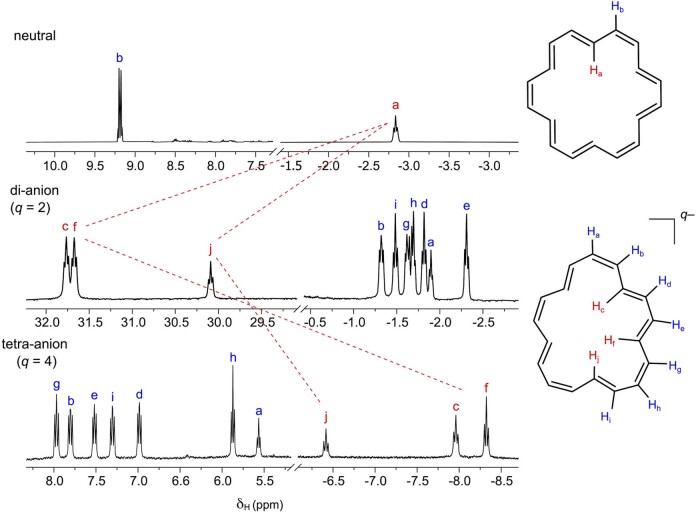
Overview of ^1^H NMR spectra. Comparison of the ^1^H NMR spectra of neutral **1** (THF-*d*_8_, 500 MHz, –40 °C), di-anion **1·Li**_**2**_ (at –60 °C) and tetra-anion **1**_**2**_**·Li**_**8**_ (at –40 °C). All spectra in THF-*d*_8_, 500 MHz.

**Extended Data Fig. 2:**
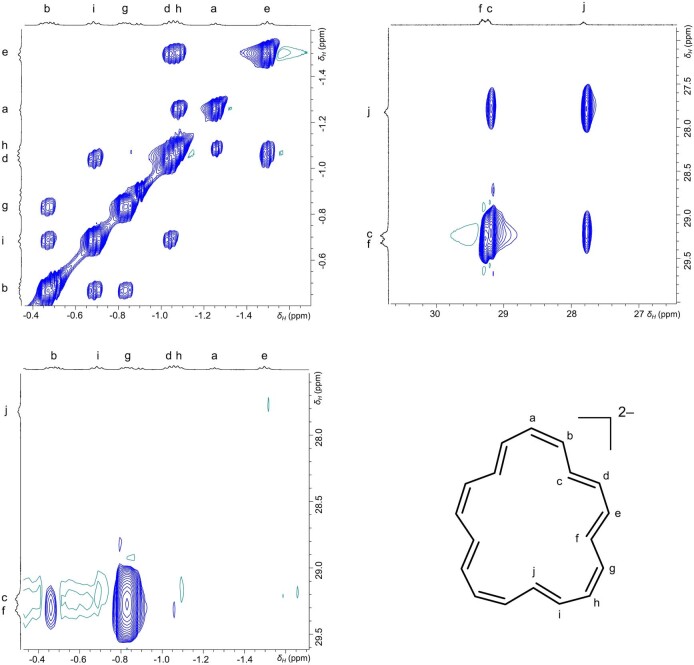
Fragments of the ^1^H-^1^H EXSY spectrum of in-situ generated di-anion 1·K_2_. This spectrum characterizes the dynamic exchange processes in **1·K**_**2**_ (THF-*d*_8_, –70 °C, 500 MHz, mixing time 0.0 sec).

**Extended Data Fig. 3:**
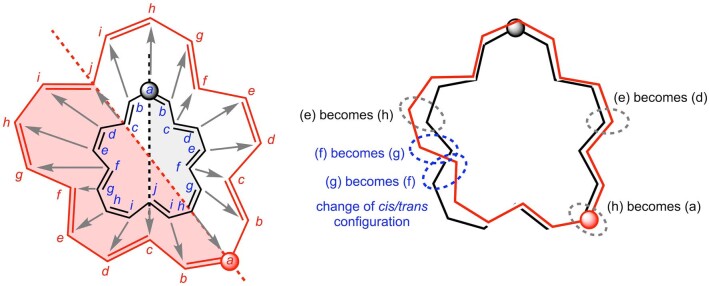
The dynamic exchange processes in **1·K**_**2**_. This diagram shows which environment undergo direct exchange, based on the observed ^1^H-^1^H EXSY NMR correlations.

Reduction of [18]annulene with potassium gives the paramagnetic radical anion^5^, followed by the dianion. By contrast, we found that reduction with lithium gives the monoanion, then the dianion, then the **1**^**4−**^ tetraanion. All ten ^1^H NMR multiplets in the spectra of the lithium salts of **1**^**2−**^ and **1**^**4−**^ have been fully assigned (Fig. 2 and Extended Data Fig. 1). The spectra of **1**^**2−**^ and **1**^**4−**^ show that they have the same symmetry, but the five inner protons that are strongly deshielded in **1**^**2−**^ (*δ*_H_ 30–32 ppm) become strongly shielded in **1**^**4−**^ (*δ*_H_ −8 to −9 ppm), while the signals from the outer 13 protons shift in the opposite direction. This reversal is as expected when converting a 20-electron anti-aromatic ring into a 22-electron aromatic system. The ^1^H NMR spectrum of the tetraanion is essentially independent of temperature in the range of −40 to 55 °C, whereas the spectrum of the dianion becomes broad at temperatures above −50 °C due to dynamic exchange between the environments. Two-dimensional NMR exchange spectroscopy reveals that this dynamic process involves rotation of two CH units (f and g) to redefine the symmetry plane of the ring (Extended Data Figs. 2 and 3 and Supplementary Figs. 24–26). It was reported in ref. ^4^ that solutions of the potassium salt **1·K**_**2**_ decompose rapidly at temperatures above 0 °C, whereas in our hands, the solution in anhydrous THF is stable even at 40 °C and can be stored for weeks in a sealed glass tube at room temperature. Exposing a solution of **1**^**4−**^ to oxygen (O_2_) regenerates neutral **1**.

### X-ray crystallography of the tetraanion lithium salt

**Fig. 3:**
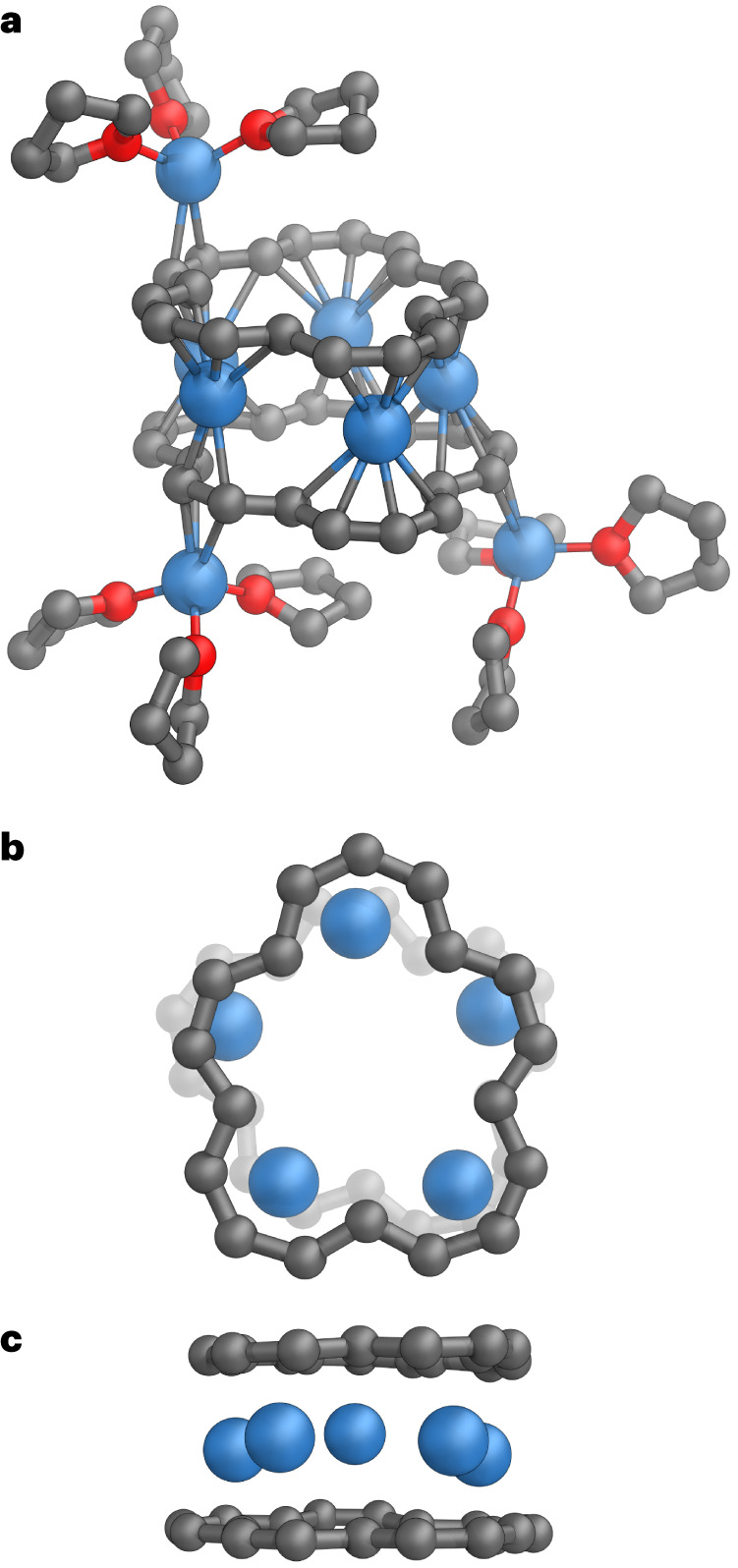
Single-crystal X-ray structure of **1**_**2**_**·Li**_**8**_. **a**, The asymmetric unit (interstitial solvent, hydrogen atoms and minor disorder components omitted for clarity) with C–Li distances in the range 2.175(4)–2.745(4) Å (internal ions) and 2.285(3)–2.591(4) Å (external ions) indicated by thin tubes. **b**,**c**, Two orthogonal views on the sandwich part: top view (**b**) and side view (**c**). Colour code: C, grey; O, red and Li, blue.

The lithium salt of **1**^**4−**^ was crystallized by layered addition of hexanes to a solution in THF. The geometry of the **1**^**4−**^ tetraanion from single-crystal X-ray diffraction analysis (Fig. 3) is fully consistent with the ^1^H NMR spectra of **1**^**2−**^ and **1**^**4−**^ (Figs. 1 and 2). To our surprise, the crystal structure reveals a sandwich complex composed of two tetraanionic macrocycles with five Li^+^ ions intercalated between them and three external Li^+^ cations each coordinated to three THF ligands (Fig. 3). The molecular formula of the solid-state tetraanion salt can be written as (THF)_3_Li//**1**//Li_5_//**1**//[Li(THF)_3_]_2_ and we use the simplified abbreviation **1**_**2**_**·Li**_**8**_. Formation of sandwich columnar species by **1** has been postulated theoretically, although those models assumed that the conformation of the neutral [18]annulene is preserved on reduction^6^.

In the crystal structure of **1**_**2**_**·Li**_**8**_, the distance between centroids of the rings is 3.8926(6) Å and the *C*_2_ axes of the stacked C_18_H_18_^4−^ units are rotated with respect to each other by a torsional angle of 74° (where the axis of rotation is the line connecting the centroids, Supplementary Fig. 7). The molecules stack to form columns in the solid state, due to C–H···*π* interactions between the coordinated THF molecules and anions (Supplementary Fig. 9). The C–C bond lengths in the [18]annulene skeleton are in the range of 1.390(3)–1.436(3) Å (mean 1.411 Å), which is slightly longer than the mean C–C bond length in neutral [18]annulene (1.395 Å). There is no evidence for bond-length alternation (BLA < 0.02 Å; see Supplementary Information for details), as expected for an aromatic system. Density functional theory (DFT) calculations at the BLYP45/def2-TZVP level confirm the absence of BLA in the **1**^**4−**^ anion in the gas phase. This contrasts with the situation in the neutral macrocycle. The crystal structure of neutral **1** shows no indications of BLA^7–10^, but computational studies conclude that the molecule rapidly interconverts between bond-alternate structures (for example, at BLYP45/def2-TZVP, the BLA in neutral **1** is 0.039 Å)^11–16^. Although the neutral molecule has approximate *D*_6h_ symmetry, its symmetry is reduced by BLA and by the fact that the inner hydrogen atoms may sit slightly out of plane^8^.

### ^7^Li NMR spectroscopy of the tetraanion lithium salt

^7^Li NMR spectroscopy shows that the sandwich complex is formed in solution in THF, as well as in the solid state. Thus, the spectrum of the tetraanion (recorded at −80 °C in THF-*d*_8_) gives signals for two types of lithium environment: a sharp peak at *δ*_Li_ = −15.66 ppm for intercalated lithium cations, strongly shielded by the two aromatic **1**^**4−**^ rings, and a broad peak at −2.0 ppm for the external lithium cations. By contrast, the anti-aromatic dianion gives a ^7^Li NMR spectrum (recorded at −60 °C in THF-*d*_8_) with one broad peak at *δ*_Li_ = 2.43 ppm, indicating weak interactions of the Li^+^ ions with the dianion (*δ*_Li_ = 0 ppm for LiCl in THF-*d*_8_).

### Formation of a heteroleptic sandwich with corannulene

**Fig. 4:**
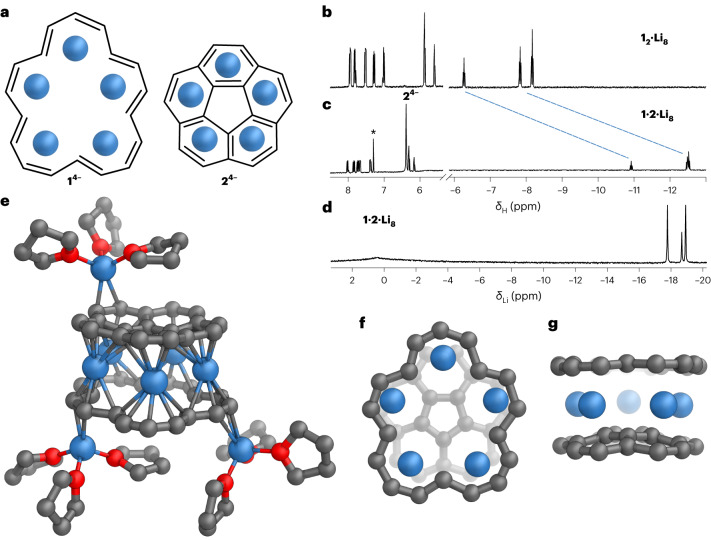
Formation of a heteroleptic sandwich. **a**, Observed conformation of **1**^**4−**^ and the corannulene tetraanion **2**^**4−**^. **b**,**c**, ^1^H NMR spectra of **1**_**2**_**·Li**_**8**_ (**b**) and **1·2·Li**_**8**_ (**c**) (500 MHz, THF-*d*_8_, 25 °C; * denotes a trace of benzene). **d**, ^7^Li NMR spectrum of **1·2·Li**_**8**_ (194 MHz, THF-*d*_8_, −80 °C). **e**, The single-crystal X-ray structure of **1·2·Li**_**8**_ with minor disorder components and hydrogen atoms omitted for clarity and with C–Li distances in the range 2.180(15)–2.694(16) Å indicated by thin tubes. **f**,**g**, Two orthogonal views of the sandwich unit: top view (**f**) and side view (**g**). Colour code: C, grey; O, red; Li, blue.

Corannulene is the only hydrocarbon previously reported to form a polynuclear anion sandwich structure similar to **1**_**2**_**·Li**_**8**_, and there is a close resemblance between these structures^17–19^. The geometry of **1**^**4−**^ resembles that of corannulene (**2**, Fig. 4a) and both hydrocarbons form lithium sandwich complexes in their tetraanionic states, so we decided to test the possibility of forming a heteroleptic sandwich built from **1**^**4−**^ and **2**^**4−**^. Exposing an equimolar mixture of **1** and **2** to lithium metal in THF-*d*_8_ led to a distinct ^1^H NMR spectrum (Fig. 4b,c). The symmetry of the annulene signals is similar to that in **1**_**2**_**·Li**_**8**_ and integration of the singlet peak corresponding to corannulene suggested formation of a 1:1 complex. The most notable difference was observed for the inner [18]annulene protons, which shift to *δ*_H_ = −12.50 and −10.92 ppm, indicating that they are strongly shielded by the corannulene bowl. The ^7^Li NMR spectrum recorded at −60 °C features three highly shielded Li environments at *δ*_Li_ = −17.92, −18.81 and −19.12 ppm integrating as 2:1:2, matching the symmetry of [18]annulene anion (Fig. 4d). Variable temperature ^1^H NMR studies showed that the corannulene singlet peak becomes sharper at 40 °C; on the other hand, lowering the temperature leads to splitting of this peak, indicating that the rotation of the corannulene bowl becomes slow on the NMR time scale making its protons inequivalent.

Definitive evidence for formation of the heteroleptic sandwich came from single-crystal X-ray diffraction analysis. The molecular structure is similar to the homo-sandwich **1**_**2**_**·Li**_**8**_ (Fig. 4e) and its formula can be written as [(THF)_3_Li]_2_//**1**//Li_5_//**2**//Li(THF)_3_, abbreviated to **1·2·Li**_**8**_. Both decks coordinate externally to three cations in total, whereas five Li^+^ cations are jammed in between the rings. The five internal lithium cations lie approximately in the same plane; the root-mean-square deviations from planarity for the intercalated Li^+^ ions in **1**_**2**_**·Li**_**8**_, **1·2·Li**_**8**_ and **2**_**2**_**·Li**_**8**_ are 0.169, 0.044 and 0.010 Å, respectively. The bowl depth of the corannulene is reduced from 0.875(2) in neutral **2** to 0.549(6) Å in **1·2·Li**_**8**_, to a smaller extent than in the corannulene homo-sandwich **2**_**2**_**·Li**_**8**_ (0.288(2) Å, ref. ^17^).

### Theoretical modelling

**Fig. 5:**
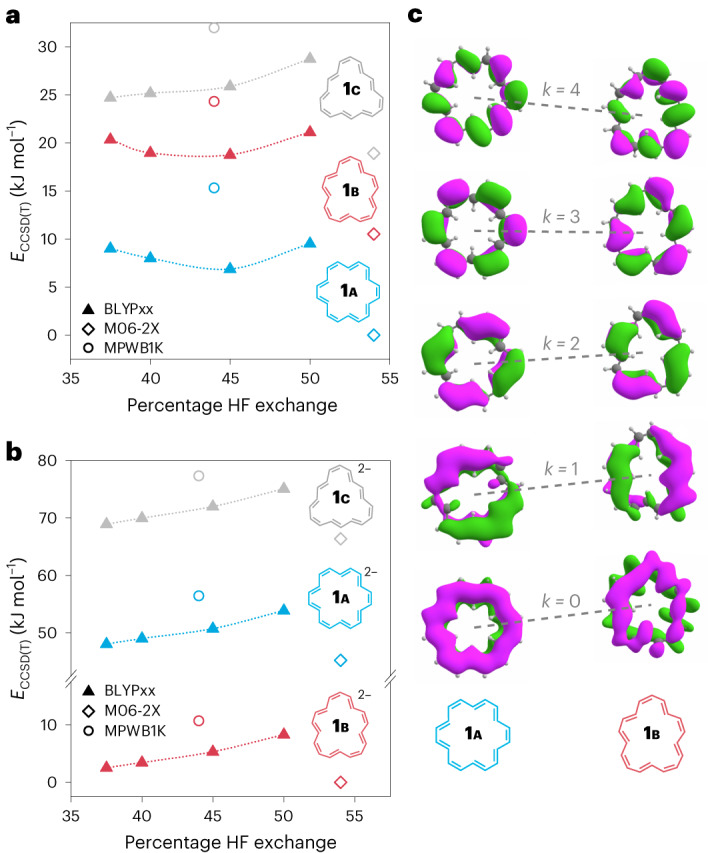
Energies of different molecular geometries for neutral [18]annulene and the dianion. **a**,**b**, DLPNO-CCSD(T*)-F12 energies of possible conformers of **1a**, **1b**, **1c** in their neutral form (**a**) and as dianions (**b**), calculated for optimized geometries obtained using M06-2X (hollow diamonds), MPWB1K (hollow circles) and BLYPxx (triangles; xx denotes the percentage of exact exchange, given on the horizontal axis). **c**, A qualitative Walsh diagram comparing π-orbital energies of **a** and **b** conformers (MOs calculated using BLYP45).

Quantum-chemical calculations, at various levels of theory (Fig. 5), confirmed that [18]annulene undergoes a dramatic change in geometry on reduction to the dianion, and provide insights into the causes of this switch in conformation. Schleyer showed that density functionals with a high proportion of exact exchange are needed to properly describe annulenes^12^. Thus, we optimized the geometries of three possible conformers (**a**, **b** and **c**) of **1**^**0**^ and **1**^**2−**^ using a range of functionals with an exact exchange of 37.5–54%. We then evaluated the energies of these optimized geometries at the highly accurate coupled clusters singles, doubles and perturbative triples level^20,21^, DLPNO-CCSD(T*)-F12 (Fig. 5a,b and Supplementary Information Section 7.3). In the neutral aromatic state, the virtually *D*_6h_ conformer **1a**^**0**^ has the lowest energy (Fig. 5a), whereas in the anti-aromatic dianion the less symmetric conformer **1b**^**2−**^ (five inner and 13 outer protons) is more stable (Fig. 5b).

Owing to the higher symmetry of **1a**, π orbitals with small angular momentum (*k* = 0 and *k* = 1; Fig. 5c) have a larger overlap in **1a** than in **1b**, leading to a lower-energy π system (*E*_π,1B_ − *E*_π,1A_ = 22 kJ mol^−1^) and an overall lower energy of **1a** (*E*_CCSD(T),1b_ − *E*_CCSD(T),1A_ ≅ 9 kJ mol^−1^). By contrast, more localized π orbitals (*k* = 4 and *k* = 5) are lower in **1b**, so adding two electrons stabilizes **1b**^**2−**^ relative to **1a**^**2−**^. Moreover, Coulombic repulsion in **1b**^**2−**^ is lower than in **1a**^**2−**^ (Supplementary Fig. 65), resulting in a strong preference for **1b**^2−^ ($E_{{\rm{CCSD}}({\rm{T}}),1{\rm{B}}^{2-}}$ − $E_{{\rm{CCSD}}({\rm{T}}),1{\rm{A}}^{2-}}$  ≅ 46 kJ mol^−1^). As the tetraanion always appears as an Li–bound dimer sandwich, the preference for **1b**^**4−**^ also can be attributed to electrostatics. In both anions, the **c** conformer proposed in ref. ^4^ (Fig. 1a) has higher energy than both **a** and **b**.

We find that the BLYP45 functional (45% exact exchange), which is a slight modification of BHLYP (Becke’s half and half functional combined with the LYP correlation functional)^22^ (50% exact exchange), gives a good account of both energies and chemical shifts in all three reduction states (Supplementary Figs. 66–68), confirming the anti-aromaticity of **1b**^**2−**^ (*δ*_out_ − *δ*_in_ = −30.2 experiment versus −28.5 calculation) and the aromaticity of **1b**^**4−**^ (*δ*_out_ − *δ*_in_ **=** +14.9 experiment versus +17.9 calculation). BLYP45 predicts that both **1b**^**2−**^ and **1b**^**4−**^ have *C*_2v_ symmetry, but **1b**^**2−**^ has significant BLA (0.06 Å on average) consistent with charge localization (Supplementary Fig. 65), whereas **1b**^**4−**^ has no BLA (<0.01 Å).

## Conclusions

Our results reveal that the postulated and widely accepted geometry of dianionic [18]annulene was incorrect, and that [18]annulene undergoes a dramatic switch in conformation on reduction. Quantum calculations demonstrate that this surprising conformational change is driven by Coulombic repulsion and optimization of π-bonding interactions. In contradiction with previous reports, this dianion is stable even at 40 °C, if it is protected from oxygen and moisture. We also show that the dianion can be further reduced to a tetraanion, which features a substantial aromatic ring current, despite forming a lithium-intercalated sandwich. This redox activity, and the ability to intercalate lithium cations, point to potential applications of annulenes as energy storage materials^23^. The global change in conformation of [18]annulene on reduction to the dianion contrasts with the behaviour of other π-conjugated macrocycles, such as [2_4_]paracyclophanetetraene, which only exhibit subtle structural changes on reduction^24^. Oth and coworkers were correct in concluding that the dianion of [18]annulene is anti-aromatic, and they made the most of the tools available to them, but the availability of high-field ^1^H NMR spectroscopy changes our view of these anions.

## Methods

[18]Annulene **1** was synthesized using the route reported in ref. ^10^, with some modifications (Supplementary Information Section 2 for details). In situ reduction experiments were carried out in the absence of air and moisture, using established techniques^25,26^, as detailed in the Supplementary Information Section 2.

DFT calculations used the def2-TZVP basis^27^, while DLPNO-CCSD(T*)-F12 calculations used the explicitly correlated cc-pVDZ-F12 basis set^28^. BLYPxx functionals, in which xx is the proportion of exact exchange, were constructed by starting from BHLYP and modifying the proportion of exact and DFT exchange, with their sum kept at 100%. Details and representative ORCA^29^ input files are given in the Supplementary Information Section 7.

## Online content

Any methods, additional references, Nature Portfolio reporting summaries, source data, extended data, supplementary information, acknowledgements, peer review information; details of author contributions and competing interests; and statements of data and code availability are available at 10.1038/s41557-024-01469-1.

### Supplementary information

Supplementary Information Supplementary Figs. 1–68, Tables 1 and 2, experimental procedures and discussion.

Supplementary data 1 Crystallographic data for compound S3; CCDC reference no. 2293564.

Supplementary data 2 Crystallographic data for compound 1; CCDC reference no. 2293565.

Supplementary data 3 Crystallographic data for compound 1_2_·Li_8_ CCDC reference no. 2293566.

Supplementary data 4 Crystallographic data for compound 1_2_·2·Li_8_; CCDC reference no. 2293567.

Supplementary data 5 Calculated *xyz* coordinates of [18]annulene neutral, dianion and tetraanion.

## Figures and Tables

**Extended Data Fig. 4:**
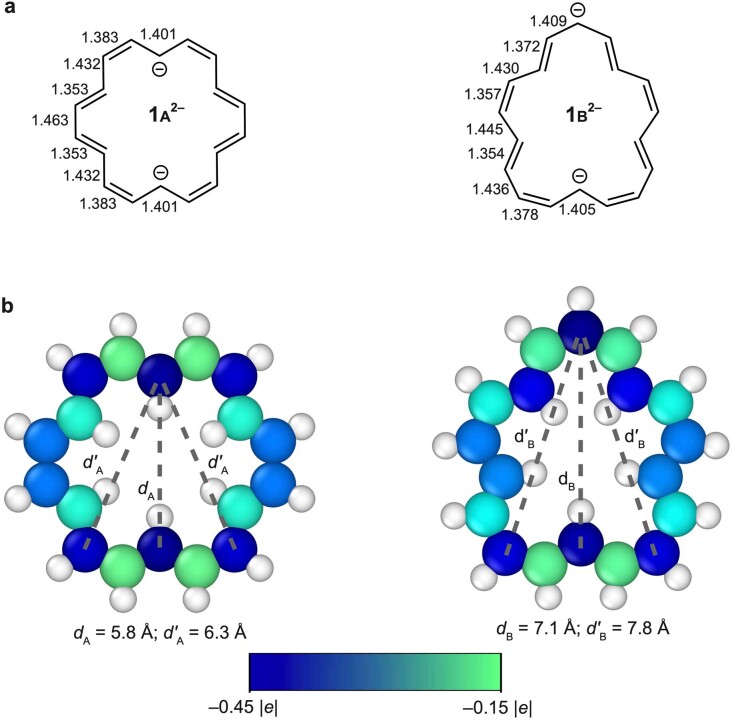
Bond lengths, charge distributions and trans-annular distances in two geometries of the dianion. **a** Bond lengths and **b** NBO charges in **1a**^2–^ (left) and **1b**^2–^ (right) calculated at the BLYP45/def2-TZVP level. Dark blue corresponds to –0.45 |*e*|, light green to –0.15 |*e*|. Distances between atoms with the largest negative charge are highlighted.

## Data Availability

All data are available in the main text or the supplementary materials. Crystallographic data for structures reported in this Article have been deposited at the Cambridge Crystallographic Data Centre, under deposition numbers CCDC 2293564 (**S3**), 2293565 (**1**), 2293566 (**1**_**2**_**·Li**_**8**_) and 2293567 (**1·2·Li**_**8**_). Copies of the data can be obtained free of charge from http://www.ccdc.cam.ac.uk/structures/.
