# Supplementary material for: The anti-aromatic dianion and aromatic tetraanion of [18]annulene
Source: Nat Chem. 2024 Mar 6;16(6):998–1002. doi: 10.1038/s41557-024-01469-1 (PMC11164681; doi:10.1038/s41557-024-01469-1)
Supplement: Supplementary file 1 — Supplementary Figs. 1–68, Tables 1 and 2, experimental procedures and discussion. [file 41557_2024_1469_MOESM1_ESM.pdf]

nature chemistry

Article https://doi.org/10.1038/s41557-024-01469-1

The anti-aromatic dianion and aromatic tetraanion of [18]annulene

In the format provided by the authors and unedited

Table of Contents page

Section 1. General experimental methods 1
Section 2. Synthetic procedures 2
Section 3. X-ray crystallography 6
Section 4. UV-vis spectroscopy 13
Section 5. NMR spectroscopy 15
Section 6. HOMA and BLA calculations 39
Section 7. Theoretical calculations 41
Section 8. References 45

1. General Experimental Methods

Tetrahydrofuran (THF) and hexanes were purchased as dry from Sigma Aldrich and then freshly distilled over sodium with benzophenone as indicator before use. Other solvents and reagents were purchased from the same company and used directly. THF-d_8 for NMR purposes was purchased from Sigma Aldrich and distilled over sodium/potassium alloy in the presence of benzophenone under reduced pressure and then stored inside an argon-filled glovebox. Alkali metals were purchased from Sigma Aldrich, washed with hexanes to remove mineral oil and stored inside a glovebox under argon. All reactions and follow-up procedures were performed in custom-made glass systems under an atmosphere of argon.

^{1}H and ^{7}Li NMR spectra were recorded on Bruker AVIII HD 400 MHz, Bruker Ascend 500 MHz or Bruker AVIII HD 600 MHz NMR spectrometers. ^{1}H NMR spectra were referenced against the residual solvent peak (THF-d_8 δ_H = 3.58 ppm, δ_C = 67.0 ppm); for ^{7}Li NMR, 0.1 M LiCl in THF-d_8 (δ_{Li} = 0.00 ppm) was used as a reference. Samples were prepared in NMR tubes inside a glovebox, wrapped with Parafilm™ and then quickly flame-sealed after removing from glovebox. UV-vis-NIR absorption measurements were carried out in a 1 cm path length quartz cuvette with a PTFE cap at 298 K using a Jasco V770 spectrophotometer. THF for UV-vis-NIR measurements was stored over lithium metal to remove traces of oxygen/moisture. Samples were prepared inside a glovebox, the cuvette was closed tightly with a PTFE cap, wrapped with Parafilm™, and removed from the glovebox.

2. Synthetic Procedures

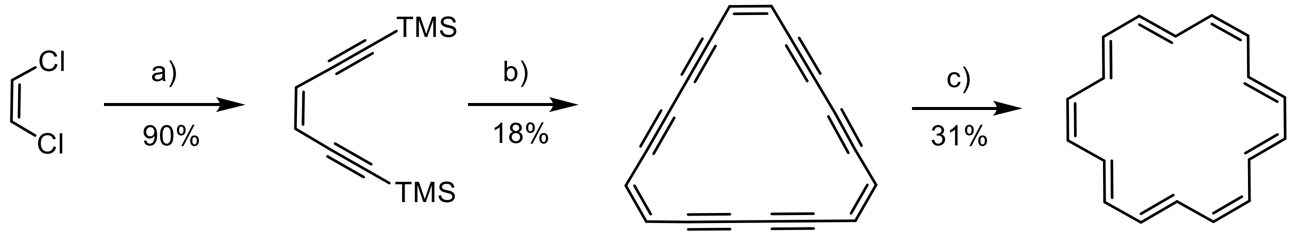

S1 **S2** **S3** **1**

Supplementary Scheme 1. Synthetic route to [18]annulene **1**.[1,2] Conditions: a) Me_3SiC_2H, 2% $Pd(PPh_3)_2Cl_2$, 4% CuI in benzene/*n*-butylamine, 20 °C (90%); (b) (i) K_2CO_3 in MeOH, 40 °C, (ii) excess $Cu(OAc)_2·H_2O$ in pyridine, 20 °C, air (18%); (c) 5% $Pd/CaCO_3/Pb$, quinoline, benzene, H_2, 20 °C (31%).

2.1 Synthesis of hexadehydro[18]annulene **S3**

Two previously reported procedures[1,2] were combined and modified: Dialkyne **S2** (2.0 g, 9.07 mmol) was deprotected first by vigorous stirring at 45 °C in MeOH (50 mL, directly from a bottle) with K_2CO_3 (3.42 g, 13.1 mmol, 1.44 equiv.) for 30 min in a closed flask under air and then allowed to reach room temperature (20 °C). The solution was decanted to remove solid salts, the salts were washed with MeOH (5 mL) and solutions combined. The resulting solution of the deprotected alkyne in MeOH was added in one portion (important!) to a solution of copper(II) acetate monohydrate (12.0 g, 59.9 mmol, 6.6 equiv.) in pyridine (200 mL, directly from a bottle) and the mixture was stirred vigorously, protected from light at 20 °C for 2 h in an open flask under air. After that time, aqueous 20% H_2SO_4 solution (100 mL) was added. The solution became warm and it was allowed to cool to room temperature. The solution was filtered on a Büchner funnel to remove precipitated copper salts, then transferred to a separatory funnel, followed by addition of water (200 mL) and saturated brine (200 mL) and shaken. Diethyl ether (120 mL) was added and the mixture was shaken. After that, another portion of diethyl ether (120 mL) was added and the funnel was slowly inverted 15 times, to avoid formation of emulsion. Two layers were visible. If they were not formed, more diethyl ether (100 mL) was added and the funnel was gently inverted a few more times. The bottom, blue aqueous layer was separated from the upper ether brown/black-colored phase containing some black particles. The ether phase was washed with 20% aqueous H_2SO_4 (5 × 100 mL) to remove the pyridine, followed by washing with water (3 × 200 mL). The solution was dried over anhydrous Na_2SO_4 and filtered.

Petroleum ether (20 mL, 40–60 °C fraction) was added to the resulting solution. The mixture was concentrated on a rotary evaporator equipped with dry ice cooling system, using an ice-cooled water bath, to remove diethyl ether, while not allowing it to become dry, otherwise decomposition is observed. Addition of petroleum ether is important to prevent all the solvent from evaporating. The receiving flask was constantly inside a bucket filled with dry ice during the evaporation process and was emptied every few minutes.

Silica gel column chromatography (4:1 petrol/EtOAc) was performed. The first fraction is orange/red and contains some very quickly decomposing compound (black decomposition product upon drying or concentrating), which is probably a cyclic dimer, but all attempts to characterize it were met with failure due to its instability. The second, intensely yellow fraction contains the desired cyclic trimer.

The yellow fraction containing trimer was collected. Benzene (15 mL) was added, hexane and ethyl acetate were removed on a rotary evaporator in the same conditions as described above. Addition of benzene is important because, in the absence of benzene, the cyclic trimer decomposes when it precipitates out of the solution. However, when benzene is added, it forms stable cocrystals[1] that precipitate during evaporation. The obtained orange-colored solution in benzene (ca. 15 mL) was transferred to a vial and stored in a freezer. Yield of **S3**: 120 mg (18%) (because of the rapid decomposition in the solid state, this yield was determined

by taking aliquot of benzene solution, weighing it and evaporating to dryness (decomposition to black material), and weighing it again, calculating the overall yield).

^{1}H NMR (400 MHz, $C_2D_2Cl_4$, 25 °C) δ 7.20 (s).
^{13}C NMR (101 MHz, $C_2D_2Cl_4$, 25 °C) δ 121.5, 83.4, 83.2.

2.2 Synthesis of [18]annulene **1**

The compound was prepared using a modified literature procedure.[1] To a 100 mL round-bottom flask, a solution of hexadehydro[18]annulene (118 mg, 0.53 mmol) in benzene (10 mL) was added, followed by an additional portion of benzene (40 mL) and quinoline (50 µL, 0.42 mmol). Nitrogen was bubbled through the suspension for 10 min to remove most of the oxygen. After that, the Lindlar catalyst (5% Pd/$CaCO_3$ poisoned with Pb, 49.0 mg, Sigma Aldrich) was added and nitrogen was bubbled for 5 min. After that, hydrogen gas from a balloon was bubbled through the solution with vigorous stirring. The progress of the reaction was monitored by TLC. Usually, the volume of two balloons was found to be enough (1 h reaction time). The mixture should not be allowed to react for too long, otherwise the product gets over-reduced. The yellow-green mixture was concentrated in vacuo and silica gel chromatography was performed (23:1:1 pentane/cyclohexane/benzene). The fraction containing the product has yellow-green color and the progress of the chromatography can be easily monitored by UV-vis spectroscopy, collecting fractions that give characteristic bands. The product **1** was dried, which provided amber solid (38 mg, 31%). Spectroscopic data were consistent with the literature.[1] The product was stored by adding benzene (10 mL) and the solution was kept at –20 °C in a freezer.

^{1}H NMR (500 MHz, THF-d_8, –40 °C) δ 9.19 (d, J = 11.6 Hz, 12H), –2.80 (t, J = 11.6 Hz, 6H).
UV-vis: (THF, 298 K) λ, log ε: 373 (5.17), 452 (4.08).

2.3 In-situ generation of **1·K$_2$**

A solution of [18]annulene **1** (1.5 mg. 6.4 µmol) in THF-d_8 (0.5 mL) was flame-sealed in an NMR tube with excess of potassium metal (10 mg, 0.256 mmol, 40 equiv.) in the form of potassium mirror in the half-length of the tube. The tube was shaken (at room temperature) and the progress of the reaction was monitored by ^{1}H NMR spectroscopy. The solution is stable at the room temperature. It can be stored for weeks, as long as the metal is not in contact with the solution as after prolonged time the color of the solution changes to brown and the NMR signals disappear, presumably due to formation of higher reduced species, resembling reactivity of corannulene for which the third reduction state is the maximum available when potassium metal was used.[3]

^{1}H NMR (500 MHz, THF-d_8, –70 °C) δ 29.47 (t, J = 11.5 Hz, 2H), 29.39 (t, J = 11.5 Hz, 2H), 27.97 (t, J = 11.5 Hz, 1H), –0.50 (dd, J = 11.5, 9.0 Hz, 2H), –0.73 (t, J = 11.9 Hz, 2H), –0.87 (dd, J = 11.9, 8.9 Hz, 2H), –1.09 (two overlapping m, 4H), –1.29 (t, J = 9.0 Hz, 1H), –1.54 (t, J = 11.5 Hz, 2H).

^{13}C NMR (from ^{1}H-^{13}C HSQC experiment, 126 MHz, THF-d_8, –70 °C) δ 144.1, 137.5, 133.6, 133.2, 128.5, 123.0, 116.4, 113.3, 103.9, 94.1.

2.4 In-situ generation of **1·Li$_2$** and **1$_2$·Li$_8$**

A solution of [18]annulene **1** (1.5 mg. 6.4 µmol) in THF-d_8 (0.5 mL) was flame-sealed in an NMR tube with excess of lithium metal (10 mg, 1.4 mmol, 226 equiv.). The tube was shaken (at room temperature) and the progress of the reaction was monitored by ^{1}H NMR spectroscopy.

Di-anion **1·Li$_2$**

^{1}H NMR (500 MHz, THF-d_8, –60 °C) δ 31.76 (t, *J* = 12.6 Hz, 2H), 31.67 (t, *J* = 12.6 Hz, 2H), 30.09 (t, *J* = 12.6 Hz 1H), –1.32 (dd, *J* = 9.6 and 12.6 Hz, 2H), –1.48 (t, *J* = 12.3 Hz, 2H), –1.62 (dd, *J* = 9.0 and 13.5 Hz, 2H), –1.69 (overlapping m, 2H), –1.82 (t, *J* = 10.6 Hz, 2H), –1.90 (t, *J* = 9.6 Hz, 1H), –2.31 (t, *J* = 10.6 Hz, 2H).

^{13}C NMR (from ^{1}H-^{13}C HSQC experiment, 126 MHz, THF-d_8, –60 °C) δ 144.5, 138.4, 134.5, 133.3, 130.2, 122.6, 118.9, 116.0, 104.6, 95.1

^{7}Li NMR (194 MHz, THF-d_8, –60 °C) δ 2.43.

Tetra-anion **1$_2$·Li$_8$**

^{1}H NMR (500 MHz, THF-d_8, 25 °C) δ 7.94 (dd, *J* = 12.1, 9.9 Hz, 2H), 7.81 (dd, *J* = 13.1, 8.8 Hz, 2H), 7.52 (dd, *J* = 11.7, 8.5 Hz, 2H), 7.28 (dd, *J* = 12.4, 9.3 Hz, 2H), 7.01 (dd, *J* = 11.6, 8.5 Hz, 2H), 5.87 (t, *J* = 9.5 Hz, 2H), 5.60 (t, *J* = 8.8 Hz, 1H), –6.27 (t, *J* = 12.4 Hz, 1H), –7.83 (t, *J* = 12.4 Hz, 2H), –8.17 (t, *J* = 12.0 Hz, 2H).

^{13}C NMR (from ^{1}H-^{13}C HSQC experiment, 126 Hz, THF-d_8, 25 °C) δ 105.7, 105.1, 104.1, 101.5, 94.4, 93.8, 93.5, 91.5, 86.7, 84.5.

^{7}Li NMR (194 MHz, THF-d_8, –40 °C) δ –15.66.

2.5 Crystallization of $[Li(THF)_3]_2//[Li_5(C_{18}H_{18})_2]//[Li(THF)_3]_2^-]$ (**1$_2$·Li$_8$**)

THF (1.0 mL) was added to a customized glass system (Supplementary Figure 1)[4] containing [18]annulene **1** (3.0 mg, 0.012 mmol) and excess of Li metal (10 mg, 1.4 mmol). The reaction mixture was stirred at 25 °C under argon for 1.5 h. The initial yellow color (neutral macrocycle) changed to brownish-green in 15 minutes. It gradually became grass green over the next 30 min. After another 30 min, the color changed to dark green and the mixture was filtered and the green filtrate was layered with anhydrous hexanes (2.0 mL). The ampule was sealed under argon and stored at 5 °C. Dark green plates started appearing after two days. After a week, crystals were taken for X-ray measurements. Yield: 1.6 mg, 20%.

2.6 In-situ generation of **1·2·Li$_8$**

A solution of [18]annulene (1.20 mg, 0.005 mmol, 1 equiv.) and corannulene (1.28 mg, 0.005 mmol, 1 equiv.) in THF-d_8 (0.5 mL) was flame-sealed in an NMR tube together with excess of lithium metal (10 mg, 1.4 mmol, 288 equiv.) under argon. The tube was shaken for 1 h until the color changed from yellow through green to brown. Precipitation was observed.

^{1}H NMR (500 MHz, THF-d_8, 25 °C) δ 8.01 (dd, *J* = 13.0, 8.7 Hz, 2H), 7.84 (dd, *J* = 12.4, 9.5 Hz, 2H), 7.73 (dd, *J* = 12.4, 9.5 Hz, 2H), 7.67 (dd, *J* = 11.8, 8.3 Hz, 2H), 7.38 (dd, *J* = 11.5, 8.3 Hz, 2H), 6.39 (s, 10H), 6.31 (t, *J* = 9.5 Hz, 2H), 6.16 (t, *J* = 8.7 Hz, 1H), –10.92 (t, *J* = 12.4 Hz, 2H), –12.50 (two overlapping t, 4H).

^{13}C NMR (151 MHz, THF-d_8, 25 °C) from ^{1}H-^{13}C HSQC and HMBC experiments δ 111.7 (cor. HMBC) 101.9, 99.2, 98.4, 96.1, 94.4 (cor. HMBC), 91.4, 89.04, 89.03, 86.7, 85.3 (cor. HSQC), 82.4, 82.0

^{7}Li NMR (194 MHz, THF-d_8, –80 °C) δ –17.92 (2Li), –18.81 (1Li), –19.12 (2Li).

2.7 Crystallization of **1·2·Li$_8$**

[18]annulene (1.20 mg, 0.005 mmol, 1 equiv.) and corannulene (1.28 mg, 0.005 mmol, 1 equiv.) were dissolved in THF (0.5 mL) and reacted with excess of lithium metal (10 mg, 1.4 mmol, 288 equiv.). Within 10 min, color changed to green and then after 1 h to brown, precipitate was present. After filtration, solution was transferred to an ampule, layered with anhydrous hexanes (1.0 mL) and the ampule was flame sealed and placed at 5 °C for one week. Crystals appeared as brown plates. Yield: 1.9 mg (30%).

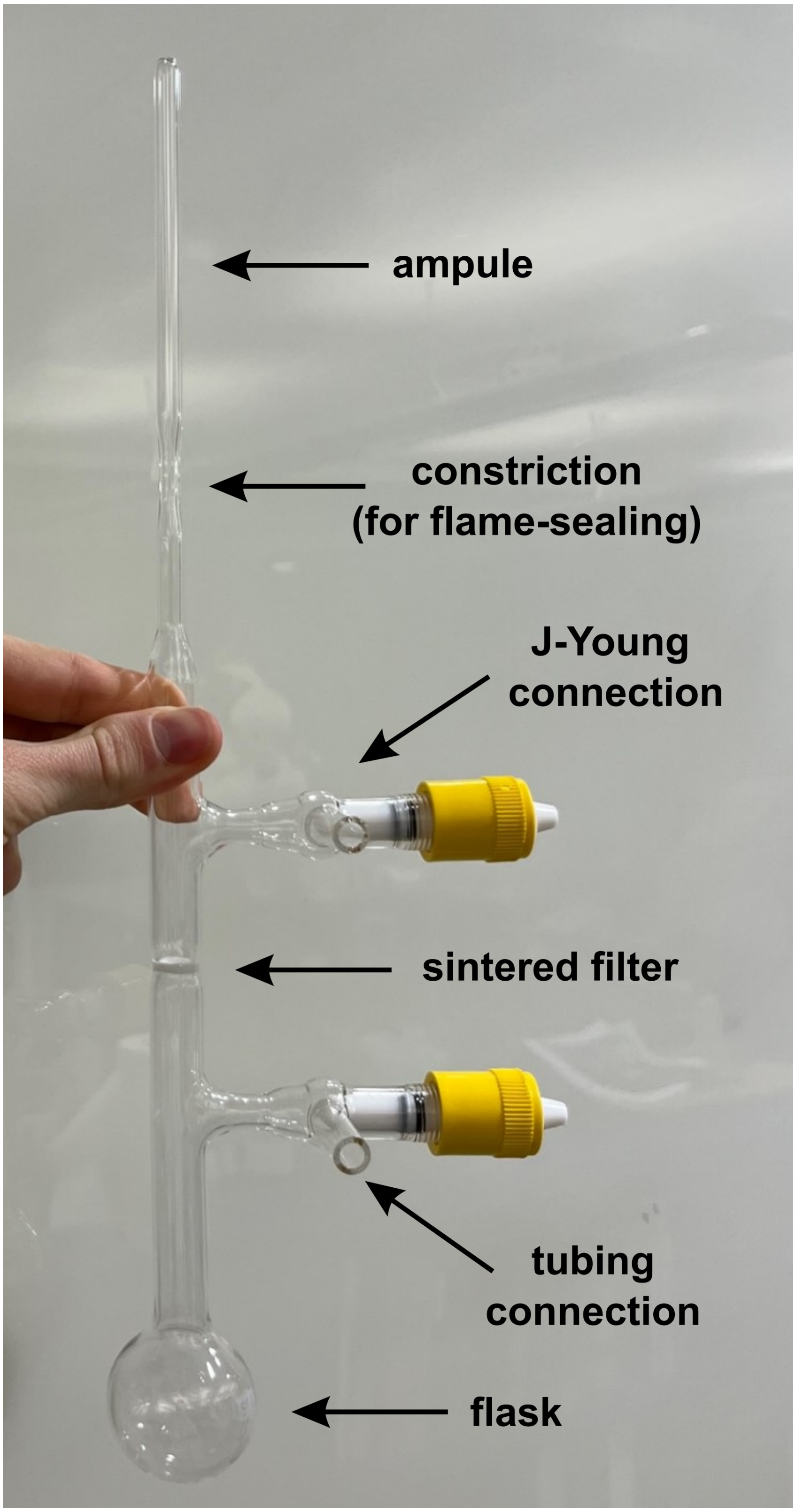

Supplementary Figure 1. Glassware used for reducing [18]annulene and crystallizing the anion salts.[4] The solvent, [18]annulene and alkali metal were originally introduced into the flask via the lower J-Young tap, under a high flow of argon. After reaction, the apparatus was inverted so that the solution flows through the sinter into the ampoule. Then the filtrate was carefully layered with hexane to crystallize the product. Finally, the ampule was flame-sealed under slightly reduced pressure and placed in a fridge. After successful crystallization, the ampule was placed in a glovebox, opened, and the crystals were immersed in dry oil on a glass slide. The slide was removed from the glovebox and quickly placed under microscope and appropriate crystal was selected and mounted on a diffractometer.

3. X-ray Crystallography

3.1. Measurement and refinement details

Data collection on **1** was performed at the Department of Chemistry, University of Oxford using Rigaku Super Nova A diffractometer at 150 K and then reduced using CrysAlisPro.

Data collection for **S3** and **1·2·Li$_8$** was performed at the Department of Chemistry, University of Oxford using Rigaku Synergy DW diffractometer equipped with rotating anode source and HyPix-Arc 150° detector at 100(2) K and subsequently reduced using CrysAlisPro.

Data collection for **1·Li$_8$** was performed at 100(2) K on a Huber 4-circle system with a DECTRIS PILATUS3 X 2M(CdTe) pixel array detector using ϕ scans (synchrotron radiation at λ = 0.49594 Å) located at the Advanced Photon Source, Argonne National Laboratory (NSF's ChemMatCARS, Sector 15, Beamline 15-ID-D). The dataset reduction and integration were performed with the Bruker software package SAINT (version 8.38A).[5] Data were corrected for absorption effects using the empirical methods as implemented in SADABS (version 2016/2).[6]

The structures were solved by SHELXT (version 2018/2)[7] and refined by full-matrix least-squares procedures using SHELXTL (version 2019/2)[8] software package through the OLEX2 graphical interface.[9] All non-hydrogen atoms, including those in disordered parts, were refined anisotropically. Hydrogen atoms were included in idealized positions for structure factor calculations with U_{iso}(H) = 1.2 U_{eq}(C).

Crystallization of **S3** was performed by vapor diffusion of MeOH into solution in toluene overnight and provided a new polymorph of hexadehydro[18]annulene **S3**. Measurement was performed ca. 16 h after setting up the crystallization as the crystals decompose quickly in the solid state; most of the material turned black but some crystals were still yellow and diffracted well.

Crystals of [18]annulene **1** were obtained as amber plates by vapor diffusion of methanol into toluene solution overnight. All previously described crystallization conditions for [18]annulene used low-temperature conditions and we found that it can be done reproducibly within one night, providing crystals of very good quality which diffracted providing structure with *R* factors lower than in the previously reported structures.[1,10-12] The whole molecule exhibits disorder (ratio 84:16) and was modelled using SADI restraints with default strength applied for appropriate 1,2- and 1,3-distances. The anisotropic displacement parameters of the disordered molecules were restrained to have the same U_{ij} components by using the restraint SIMU, with a standard uncertainty of 0.01 Å^2. Displacement parameters in the direction of the bonds were restrained using RIGU restraints with default strength.

1$_2$·Li$_8$ was crystallized as described in Section 2.5. In this structure model, one annulene tetra-anion and part of the other, as well as four THF molecules, were found to be disordered. This disordered molecule was modelled with two orientations with their relative occupancies refined. The geometries of the disordered parts were restrained to be similar. The anisotropic displacement parameters of the disordered molecules were restrained to have the same U_{ij} components, with a standard uncertainty of 0.01 Å^2.

1·2·Li$_8$ was crystallized as described in Section 2.7. Several crystals from different batches were tested for diffraction and the data were collected using a high flux rotating anode source. The crystals were of poor quality. Data on the best crystal were collected up to 0.9 Å resolution due to lack of diffraction spots beyond. The crystal was split. Moreover, beam damage was observed during data collection. Multiple data processing was attempted in order to get the best parameters and at the end, the structure was solved from the .hkl4 file generated during attempts of treating the data as twinned, with the second component much weaker and with much higher R_{int} and therefore not included in the refinement. The structure was found to be severely disordered. Both hydrocarbons in the sandwiched complex as well as lithium cations in between them and some of the coordinated THF molecules were treated as disordered and were modelled using SADI restraints with default strength applied for appropriate 1,2- and 1,3-distances (not including lithium cations in the list of these distance restraints). The anisotropic displacement parameters of the disordered parts were restrained to have the same U_{ij} components by using the restraint SIMU, with a standard uncertainty of 0.01 Å^2. Displacement parameters in the direction of the bonds in the disordered parts were restrained using RIGU restraints with default strength. Solvent masking (Olex2 subroutine) was used to account for electron density

from severely disordered interstitial THF molecule, indicating 304 electrons which corresponds to ca. 7.6 molecules per unit cell. Due to poor data quality and severe disorder in the structure we do not discuss this structure in detail (bond lengths etc.). Despite these problems, the obtained model provides useful information, correlates well with NMR data and therefore is fit for purpose and valuable.

Supplementary Table 1. Crystal data and structure refinement parameters for **S3**, **1**, $\mathbf{1_2{\cdot}Li_8}$ and $\mathbf{1{\cdot}2{\cdot}Li_8}$.

Identification code	**S3**	**1**	$\mathbf{1_2{\cdot}Li_8}$	$\mathbf{1{\cdot}2{\cdot}Li_8}$
CCDC number	2293564	2293565	2293566	2293567
Empirical formula	$C_{18}H_6$	$C_{18}H_{18}$	$C_{76}H_{116}Li_8O_{10}$	$C_{74}H_{100}Li_8O_9$
Formula weight	222.23	234.32	1245.2	1189.05
Temperature/K	100(1)	150(1)	100(2)	100(1)
Crystal system	orthorhombic	monoclinic	Monoclinic	monoclinic
Space group	Pbcn	$P2_1/n$	*C*2/*c*	C2/c
a/Å	7.48280(10)	10.2692(2)	46.1055(16)	44.4352(3)
b/Å	16.3801(2)	4.79470(8)	11.5268(4)	11.77190(10)
c/Å	10.26430(10)	14.8008(3)	27.3333(10)	26.6414(2)
α/°	90	90	90	90
β/°	90	108.715(2)	90.201(2)	91.2130(10)
γ/°	90	90	90	90
Volume/Å^3	1258.09(3)	690.23(3)	14526.2(9)	13932.64(18)
Z	4	2	8	8
ρ_{calc}g/cm^3	1.173	1.127	1.139	1.134
μ/mm^{-1}	0.516	0.472	0.039	0.545
F(000)	456	252	5408	5120
Crystal size/mm^3	0.15 × 0.14 × 0.09	0.37 × 0.28 × 0.04	0.02×0.05×0.11	0.2 × 0.08 × 0.04
Radiation	Cu Kα (λ = 1.54184)	Cu Kα (λ = 1.54184)	synchrotron, λ = 0.49594	Cu Kα (λ = 1.54184)
2Θ range for data collection/°	10.802 to 149.604	9.256 to 152.474	1.040 to 18.813	3.978 to 128.844
Reflections collected	19828	25079	195721	159999
Independent reflections	1292 [R_{int} = 0.0538]	1432 [R_{int} = 0.0292]	16675 [R_{int} = 0.0529]	11459 [R_{int} = 0.0764]
Data/restraints/ parameters	1292/0/82	1432/368/164	16675/1079/1141	11459/3022/1347
Goodness-of-fit on F^2	1.084	1.038	1.041	1.046
Final R indexes [I>=2σ (I)]	R_1 = 0.0322, wR_2 = 0.0871	R_1 = 0.0281, wR_2 = 0.0736	R_1 = 0.0590, wR_2 = 0.1659	R_1 = 0.1088, wR_2 = 0.3095
Final R indexes [all data]	R_1 = 0.0383, wR_2 = 0.0908	R_1 = 0.0319, wR_2 = 0.0771	R_1 = 0.0693, wR_2 = 0.1745	R_1 = 0.1222, wR_2 = 0.3223
Largest diff. peak/hole / e Å^{-3}	0.12/–0.12	0.10/–0.11	0.60/–0.38	0.54/–0.32

3.2 Structures and structural parameters

3.2.1 Hexadehydro[18]annulene **S3**

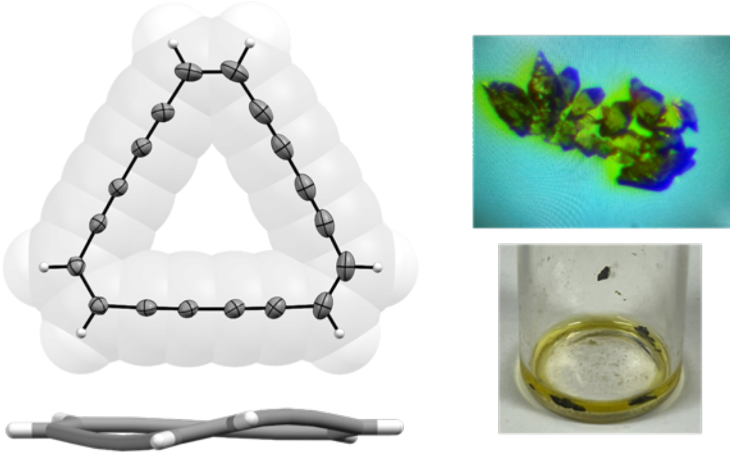

Supplementary Figure 2. Molecular structure of a new polymorph of **S3** (ellipsoids drawn at 50% probability level) and appearance of single crystals. Most of them decomposed during crystallization, forming black material. Asymmetric unit contains half of the molecule.

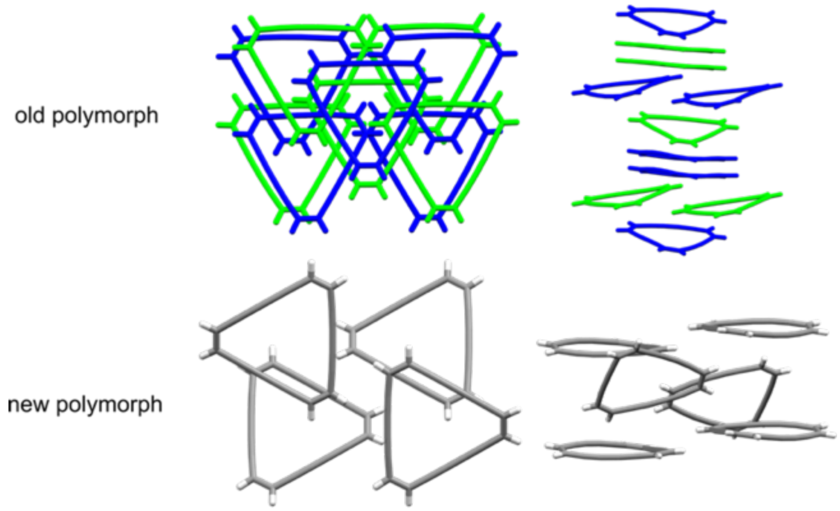

Supplementary Figure 3. Packing in crystal structures of old (CCDC 804216) and new polymorph of **S3**. Two crystallographically independent molecules are shown in blue and green.

3.2.2 [18]Annulene **1**

The obtained structure is consistent with previously reported ones[1, 10-12], the obtained R factors are lower.

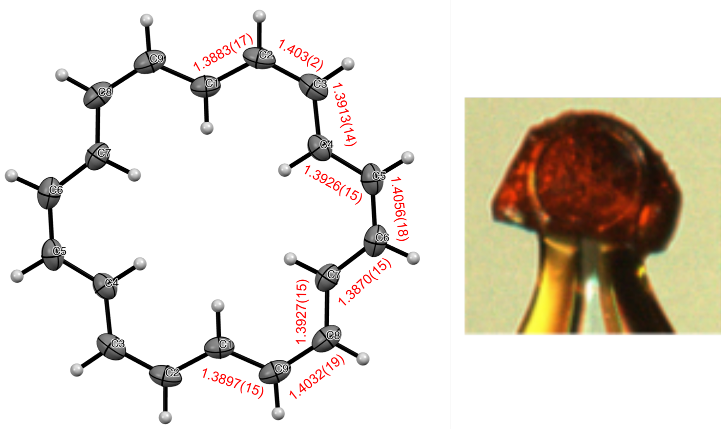

Supplementary Figure 4. Molecular structure of **1** (ellipsoids: 50% probability level) and appearance of a single crystal mounted on a goniometer head (loop diameter: 0.2 mm). The minor disorder component was omitted for clarity.

3.2.3 [18]Annulene tetra-anion sandwich **1·Li$_8$**

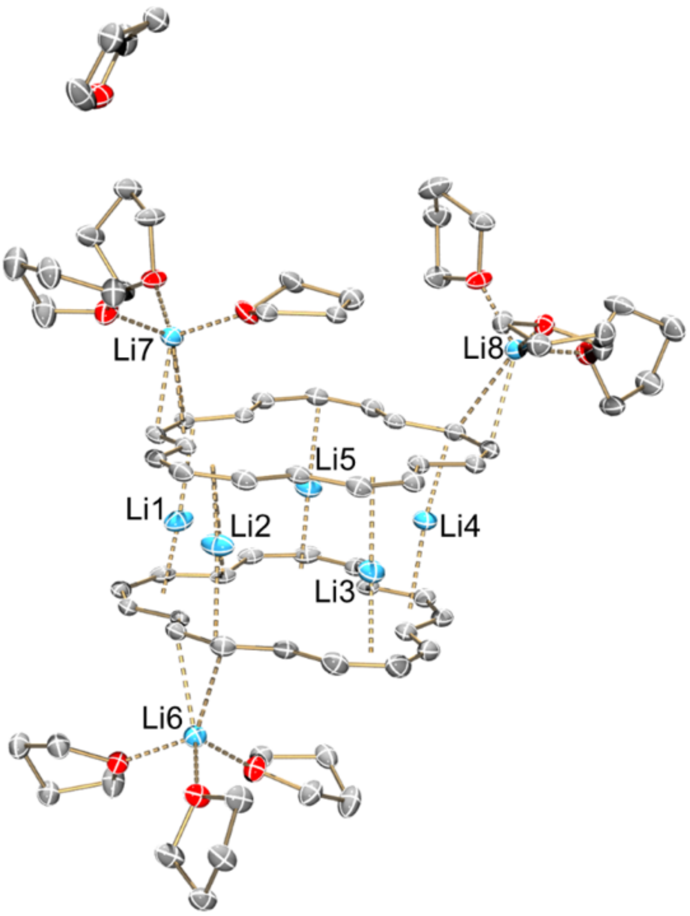

Supplementary Figure 5. ORTEP drawing of the asymmetric unit of **1$_2$·Li$_8$**, drawn with thermal ellipsoids at the 40% probability level. The interstitial THF molecules and minor disorder components are omitted for clarity. Color key: C grey, O red, and Li sky-blue.

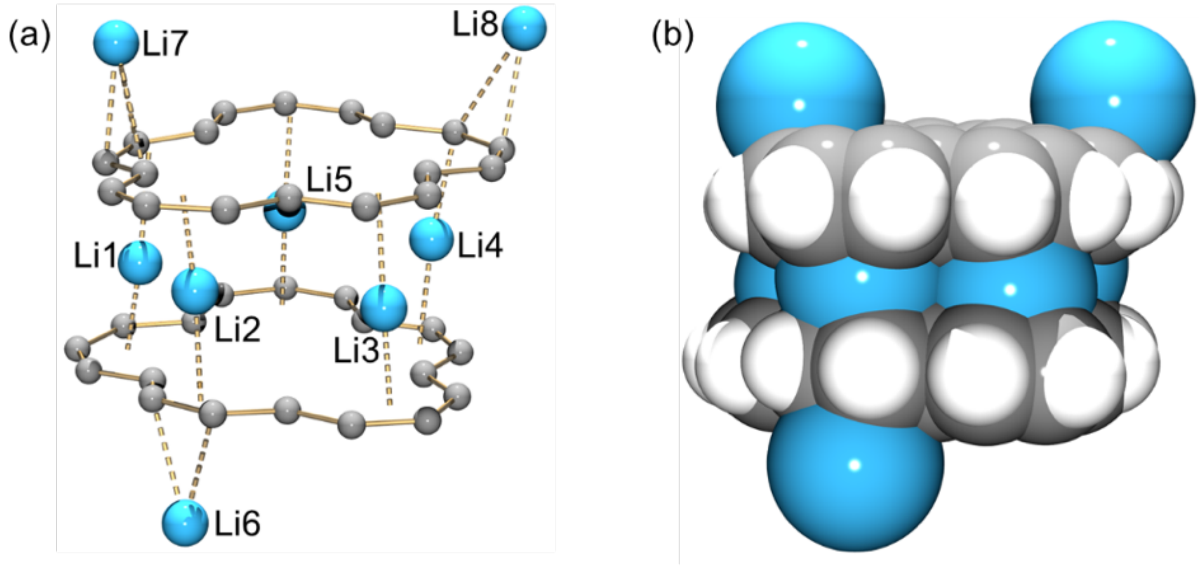

Supplementary Figure 6. Li metal binding in **1$_2$·Li$_8$**, (a) ball-and-stick model; (b) space-filling model.

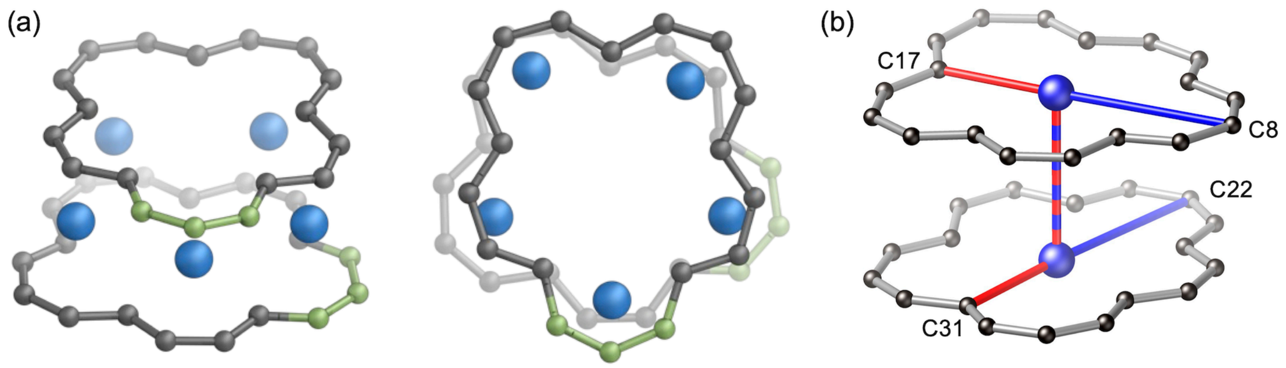

Supplementary Figure 7. Projection of the structure of $\mathbf{1_2{\cdot}Li_8}$, showing the relative orientations of the two [18]annulene tetra-anion units. (a) Including intercalated lithium ions; green-colored atoms highlight different orientation of macrocycles with respect to each other. (b) Not showing lithium cations; the centroids of the two units are shown as blue spheres [separation 3.8926(6) Å]. The torsional angle C8-centroid-centoid-C22 and C31-centoid-centoid-C17 is 74°, where the vectors C8-C17 and C22-C31 are the C_2 axes of the [18]annulene tetra-anion units.

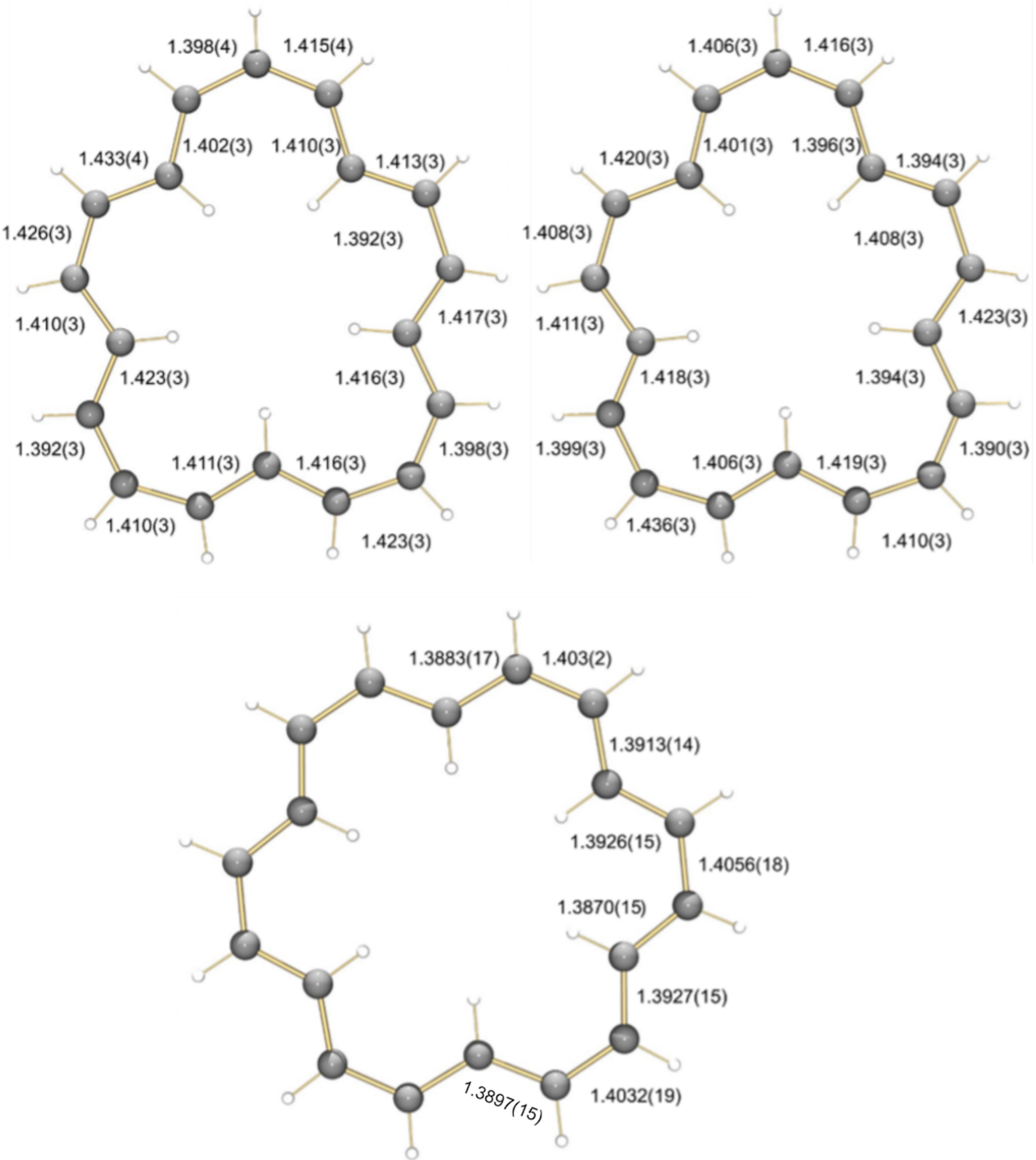

Supplementary Figure 8. C–C bond distances (Å) in $\mathbf{1_2{\cdot}Li_8}$ (top - upper and bottom decks from Fig. S6, respectively) and neutral [18]annulene **1** (bottom, crystal structure measured during our studies), ball-and-stick model. In neutral [18]annulene, two halves of the molecule are related by inversion center.

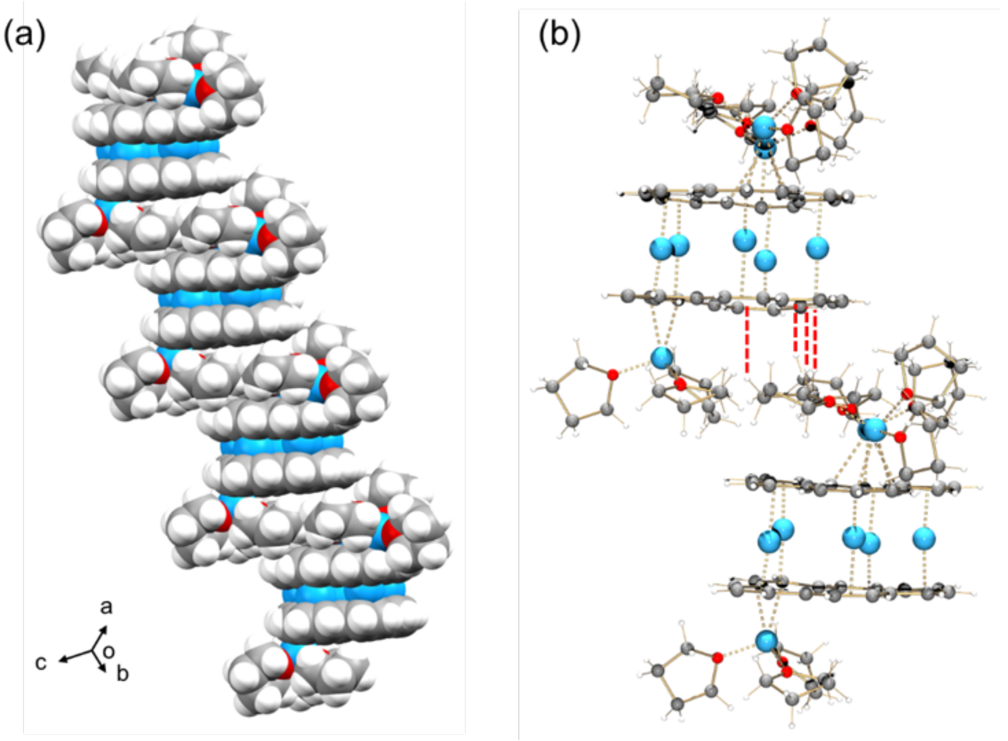

Supplementary Figure 9. (a) 1D column structure of $\mathbf{1_2{\cdot}Li_8}$, space-filling model. (b) C–H⋯π interactions (2.542(3)–2.960(3) Å) between two molecules are shown in red.

3.2.4 Heteroleptic sandwich $\mathbf{1{\cdot}2{\cdot}Li_8}$

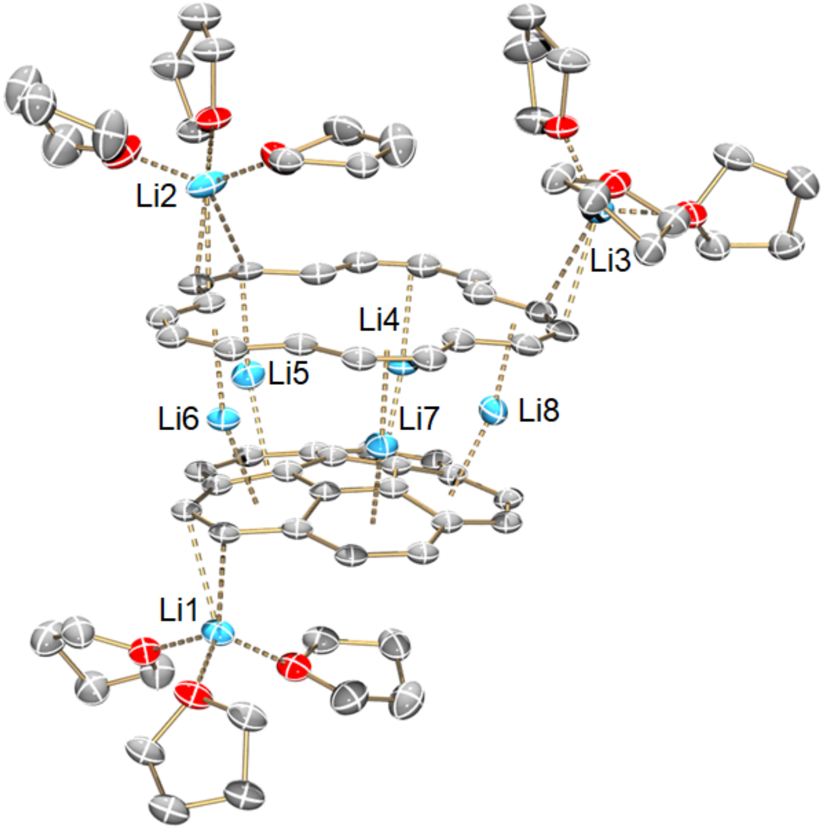

Supplementary Figure 10. ORTEP drawing of the asymmetric unit of $\mathbf{1{\cdot}2{\cdot}Li_8}$, drawn with thermal ellipsoids at the 40% probability level. The interstitial THF molecules and minor disorder components are omitted for clarity. Color key: C grey, O red, and Li sky-blue.

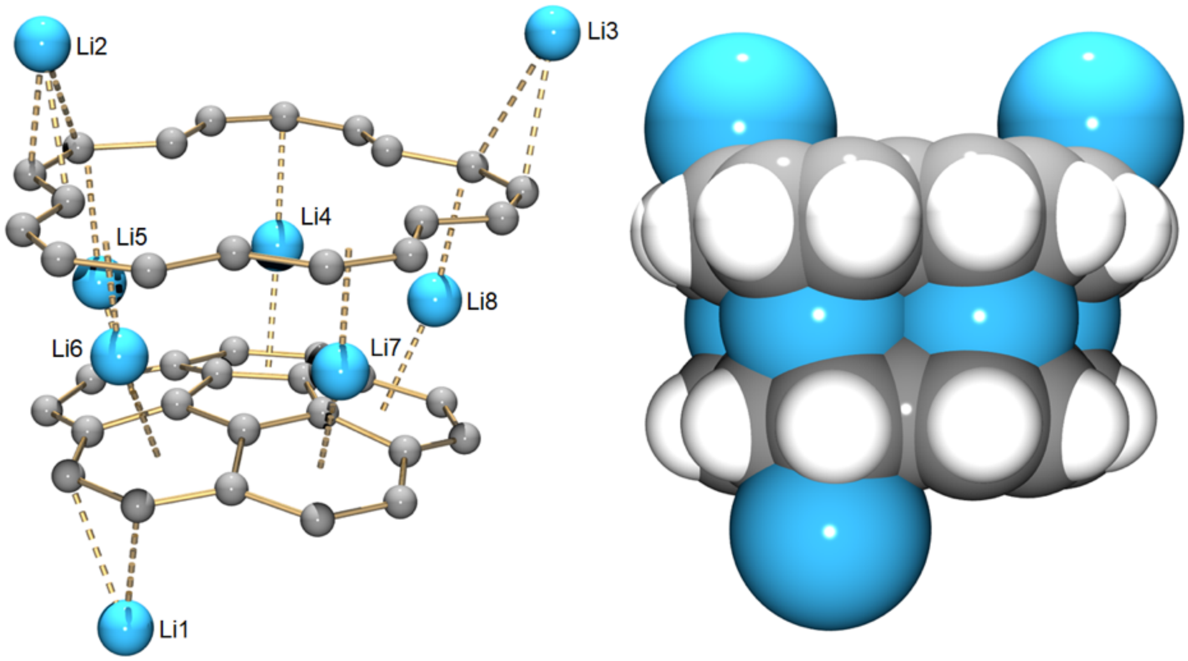

Supplementary Figure 11. Li metal binding in **1·2·Li$_8$**, (a) ball-and-stick model; (b) space-filling model.

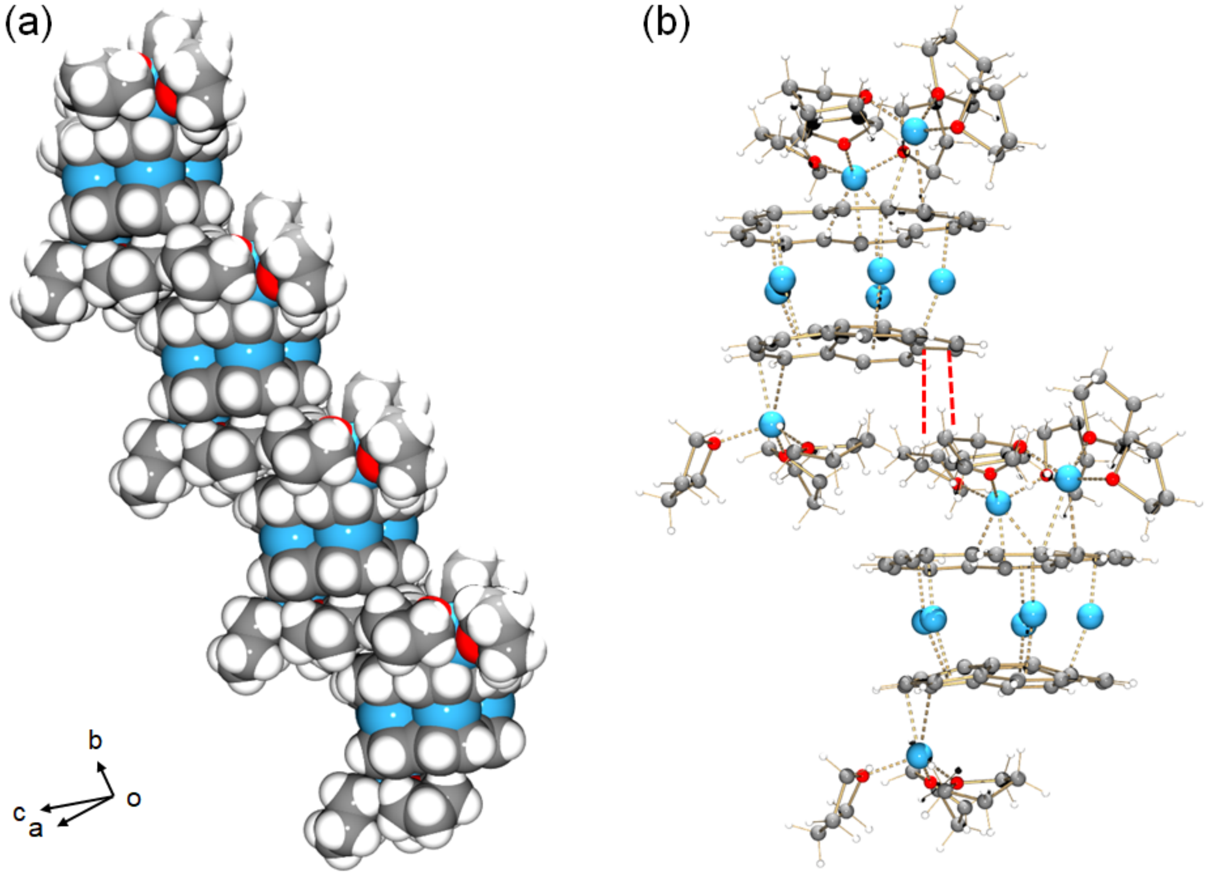

Supplementary Figure 12. (a) 1D column structure of **1·2·Li$_8$**, space-filling model. (b) C–H···π interactions (2.696–2.819 Å) between two molecules are shown in red.

4. UV-vis-NIR Spectroscopy

<u>**4.1** Stability test of [18]annulene in solution and in the solid state</u>

Equal amounts of solution of [18]annulene were dried in a stream of nitrogen gas and placed in separate vials. The samples were then stored in conditions described in the legend of Fig. S13 (below) after dissolving in equal volume of *n*-hexane or as solid, dissolving in *n*-hexane after given time. All samples were prepared in the atmosphere of air. The solid samples were placed at a freezer or in dry ice whereas the solution was kept exposed to laboratory light. The sample stored for three months as solution at room temperature (20 °C) (yellow trace in Fig. S13) was diluted to the initial volume due to evaporation of a small amount of solvent after the prolonged time.

The experiments indicate that [18]annulene **1** is not stable as solid at 20 °C, but can be stored at lower temperatures. When the sample was stored at 20 °C for two days, the main UV-vis band decreased almost three times, indicating decomposition.

Storing [18]annulene as solution for the same amount of time did not have influence on the UV-vis spectrum. After three months of storing that solution, the main band decreased ca. 1.6 times; still, less than after storing the sample as solid at the same conditions for only two days.

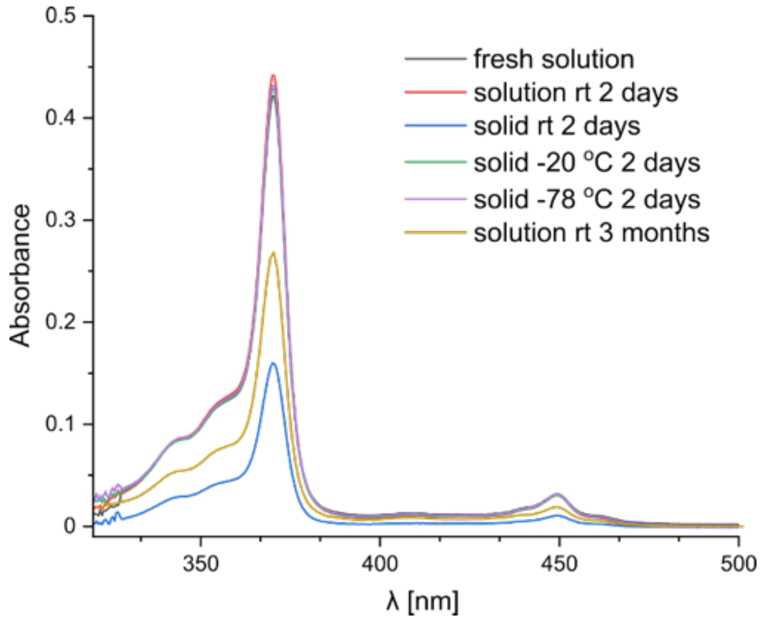

Supplementary Figure 13. Stability test of [18]annulene **1** in solution and in the solid state. *n*-hexane, 20 °C.

4.2 UV-vis-NIR monitoring of reaction of lithium metal with [18]annulene **1**

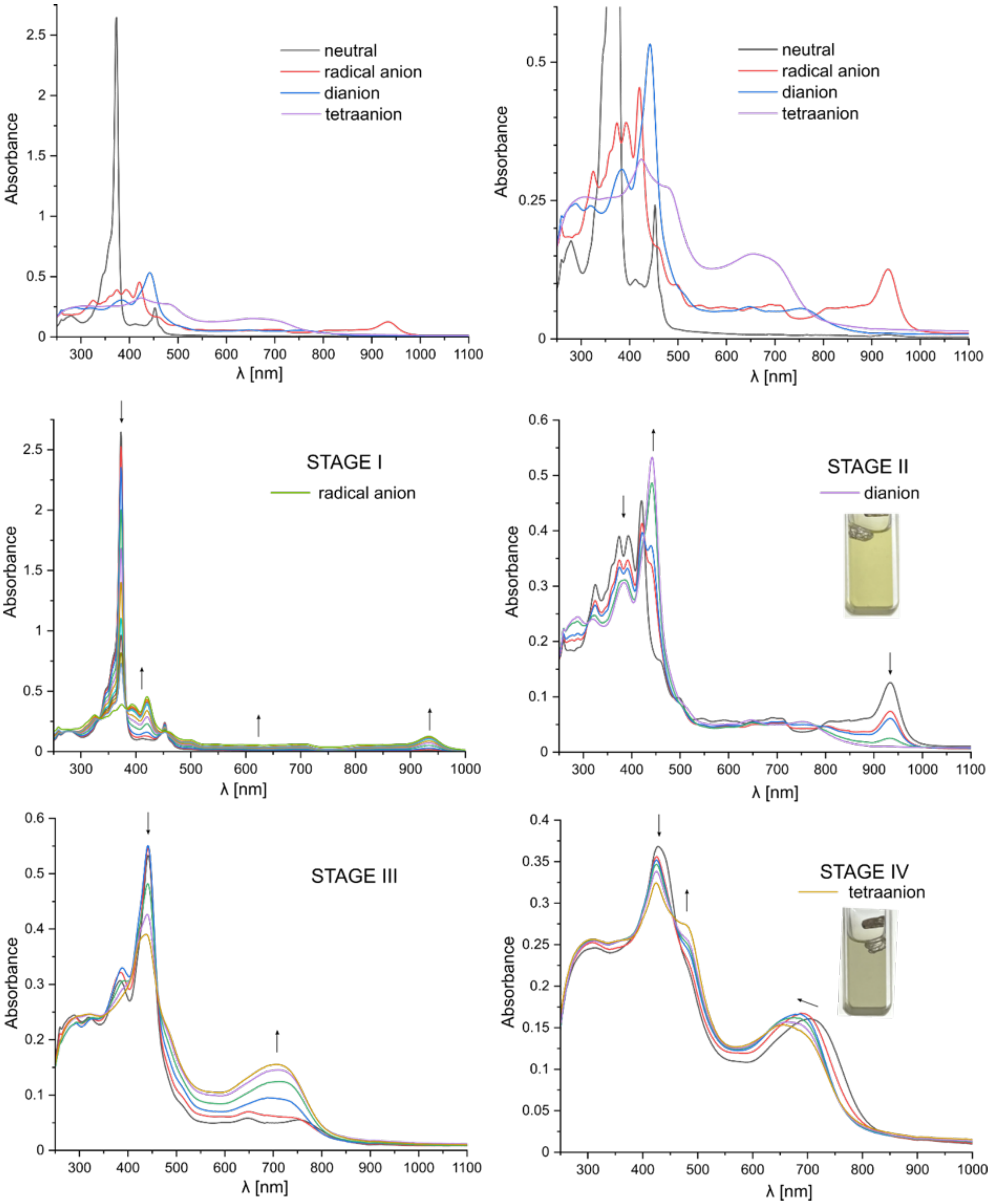

Supplementary Figure 14. Progress of UV-vis-NIR monitored reaction of **1** with Li metal. 20 °C, THF.

5. NMR Spectroscopy

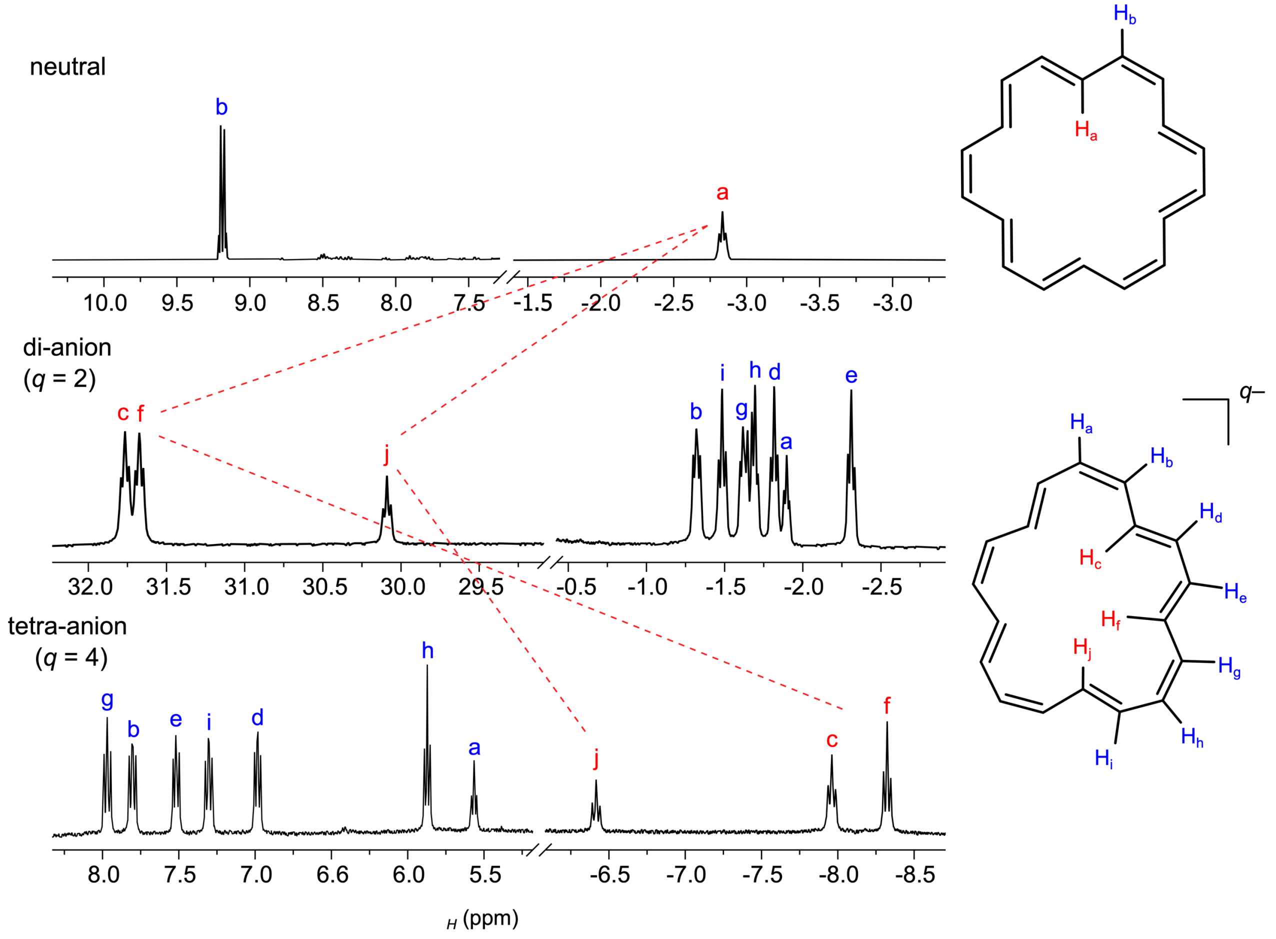

Supplementary Figure 15. Overview comparing the ^{1}H NMR spectra of neutral **1** (THF-d_8, 500 MHz, –40 °C), di-anion **1·Li$_2$** (at –60 °C) and tetra-anion **1$_2$·Li$_8$** (at –40 °C). All spectra in THF-d_8, 500 MHz.

<u>**5.1** Hexadehydro[18]annulene **S3**</u>

Data are consistent with the literature.[2] The sample was prepared by dissolving cocrystals with benzene in $C_2D_2Cl_4$ (the same solvent as used in the literature[1]), obtained during concentration on a rotary evaporator at low temperature.

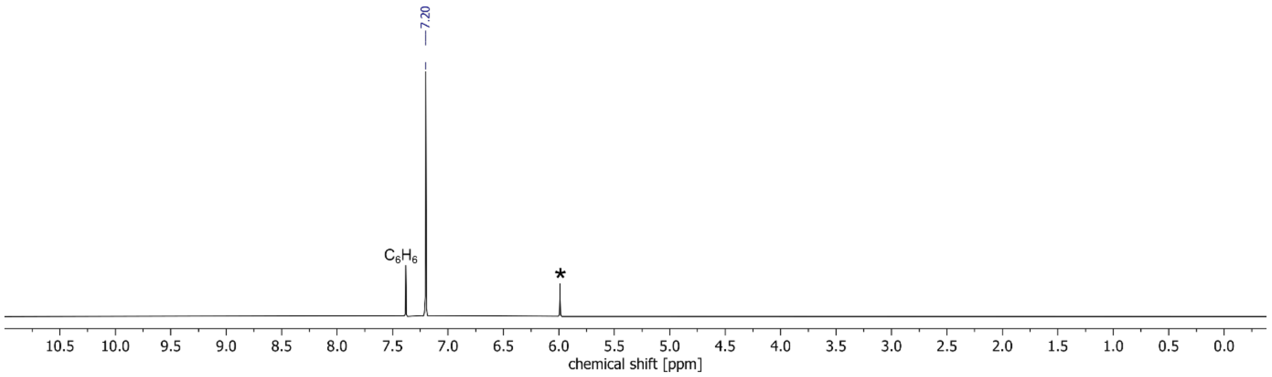

Supplementary Figure 16. ^{1}H NMR spectrum of **S3**, $C_2D_2Cl_4$, 400 MHz, 25 °C. Asterisk indicates residual solvent peak.

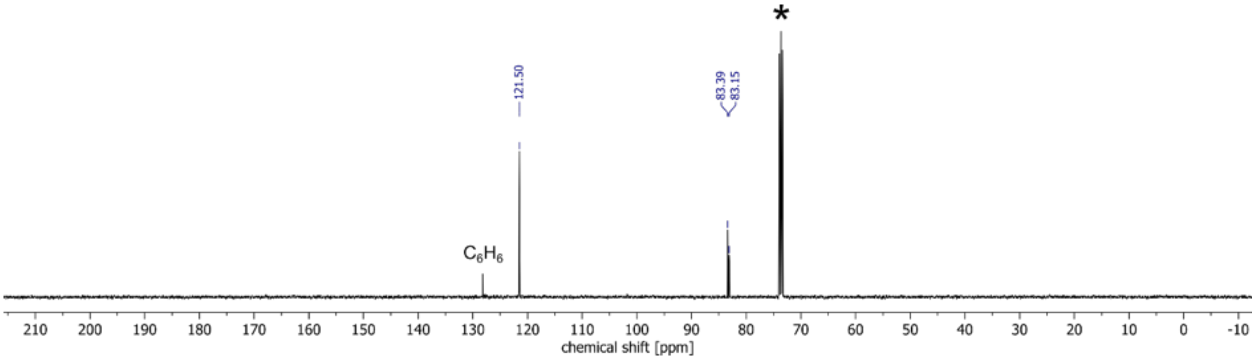

Supplementary Figure 17. ^{13}C NMR spectrum of **S3**, $C_2D_2Cl_4$, 101 MHz, 25 °C. Asterisk indicates residual solvent signal.

<u>**5.2** [18]Annulene **1**</u>

Data are consistent with the literature.[1]

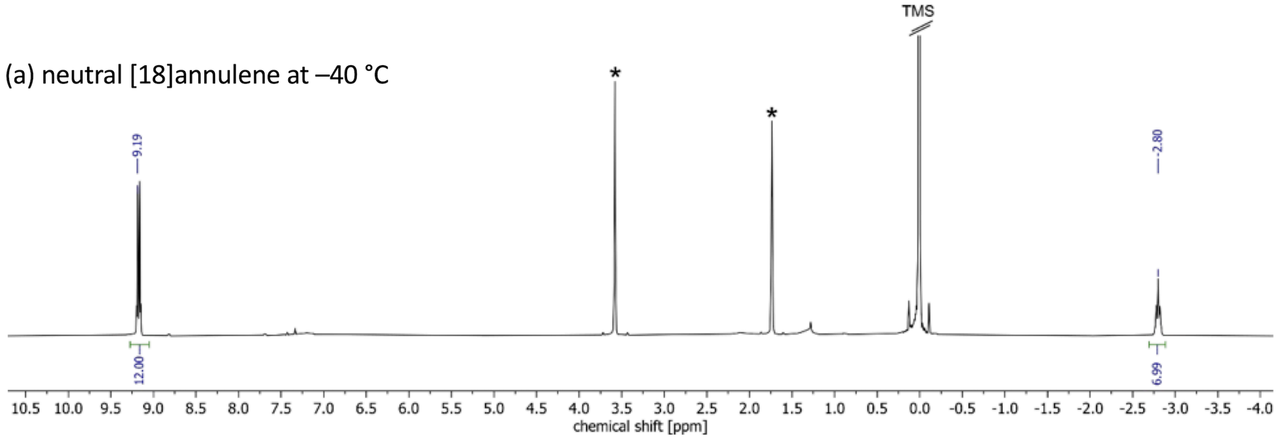

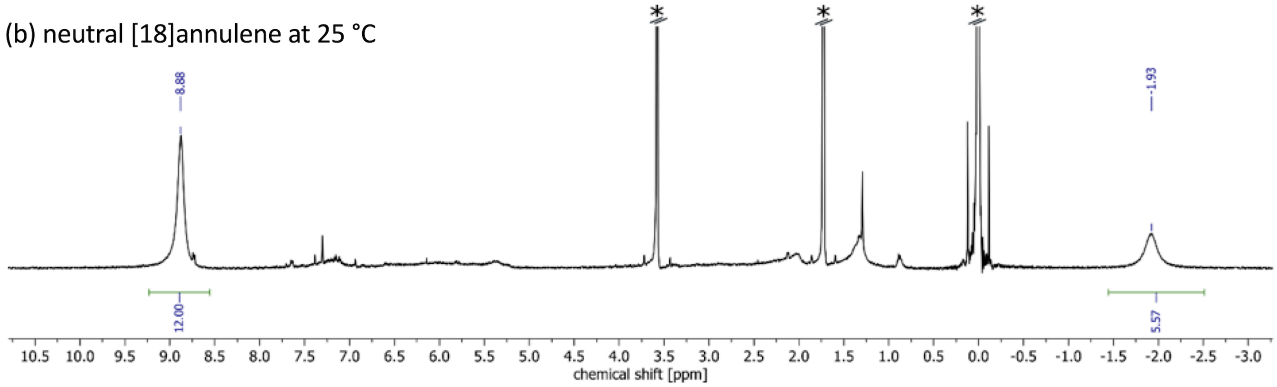

Supplementary Figure 18. ^{1}H NMR spectrum of **1**, THF-d_8, 500 MHz, (a) at –40 °C and (b) at 25 °C. Asterisks indicate residual solvent peaks and tetramethylsilane (TMS).

5.3 Potassium salt of [18]Annulene di-anion **1·K₂**

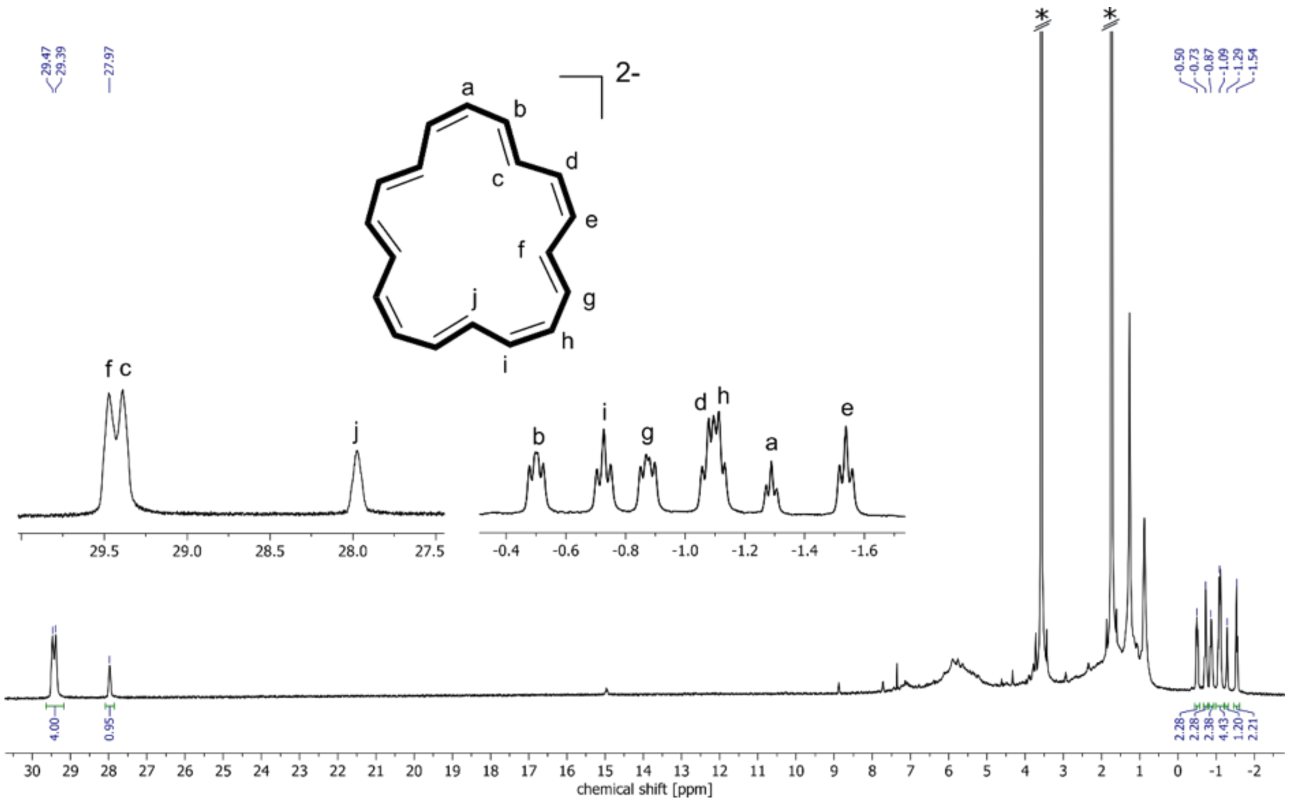

Supplementary Figure 19. ^{1}H NMR spectrum of in-situ generated di-anion **1·K₂**, THF-d_8, –70 °C, 500 MHz.

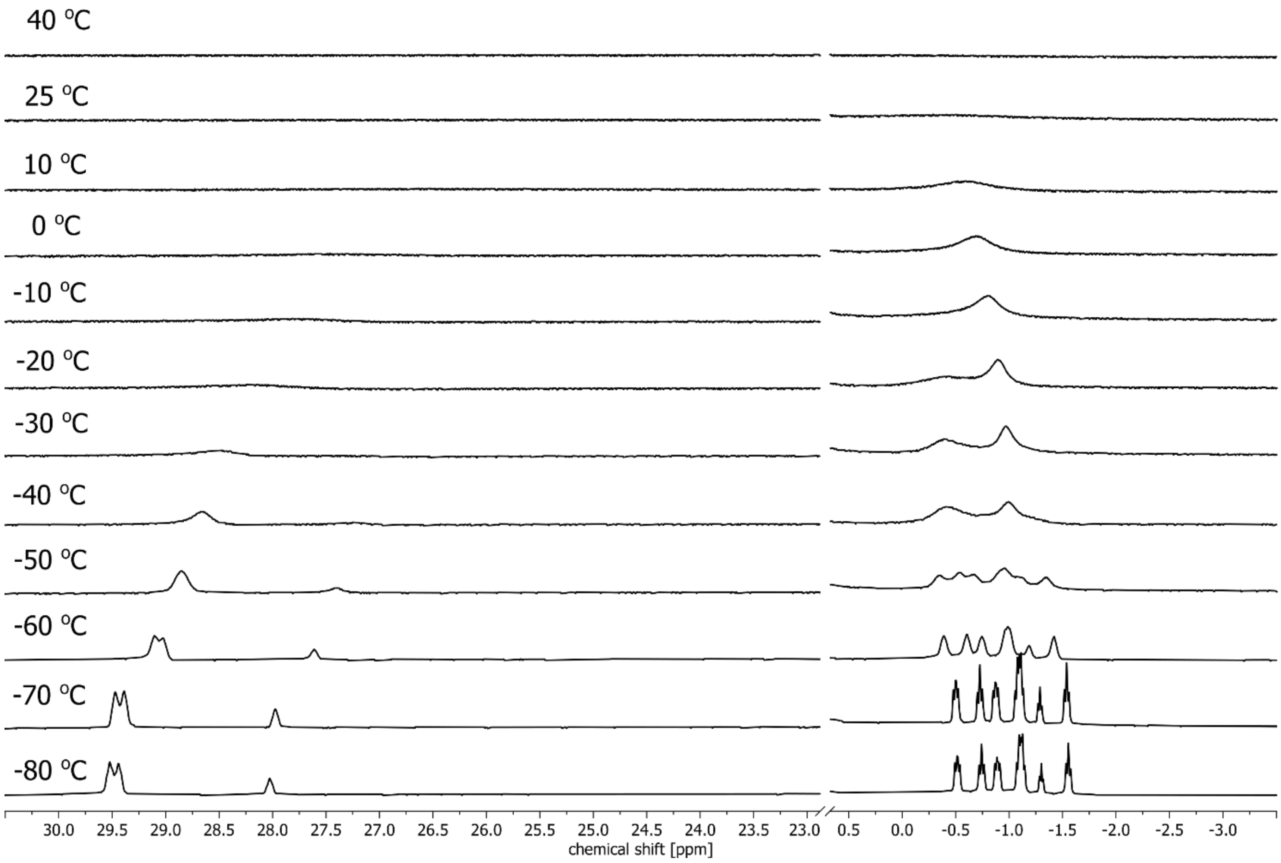

Supplementary Figure 20. Fragments of VT NMR spectra of in-situ generated di-anion **1·K₂**, THF-d_8, 500 MHz.

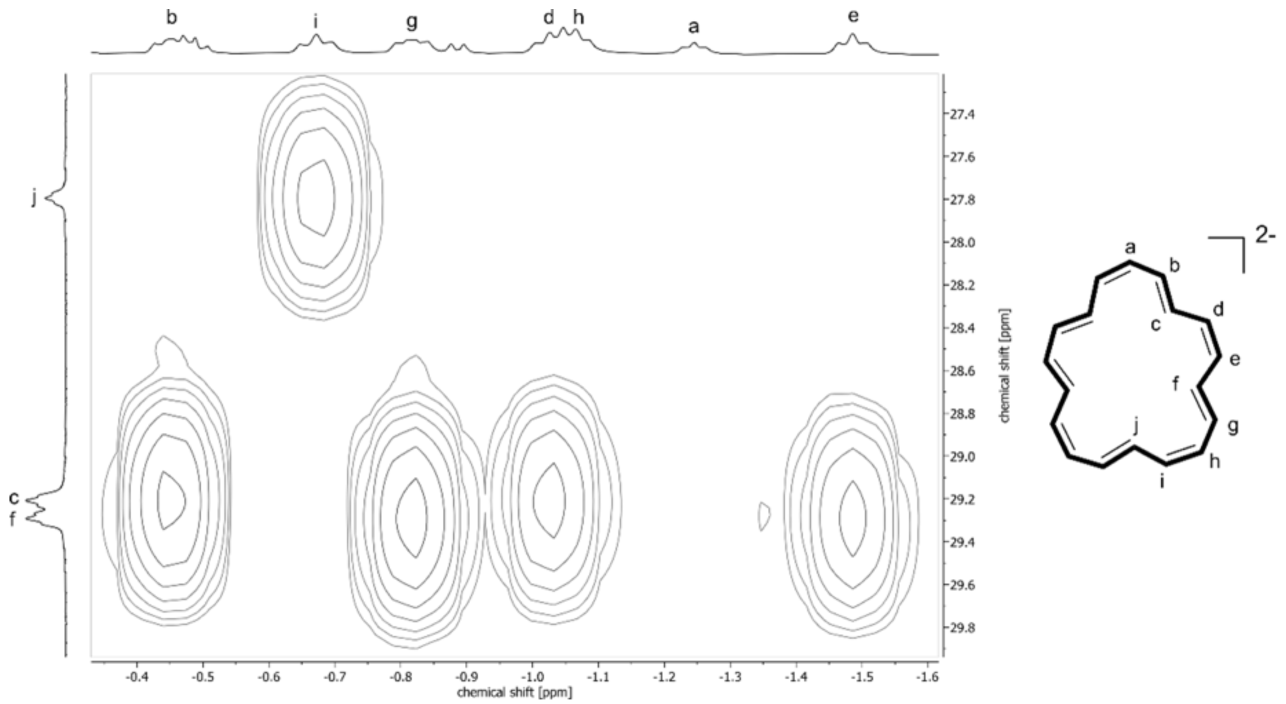

Supplementary Figure 21. Fragment of a ^{1}H-^{1}H COSY spectrum of in-situ generated di-anion **1·K₂**, THF-d_8, –70 °C, 500 MHz.

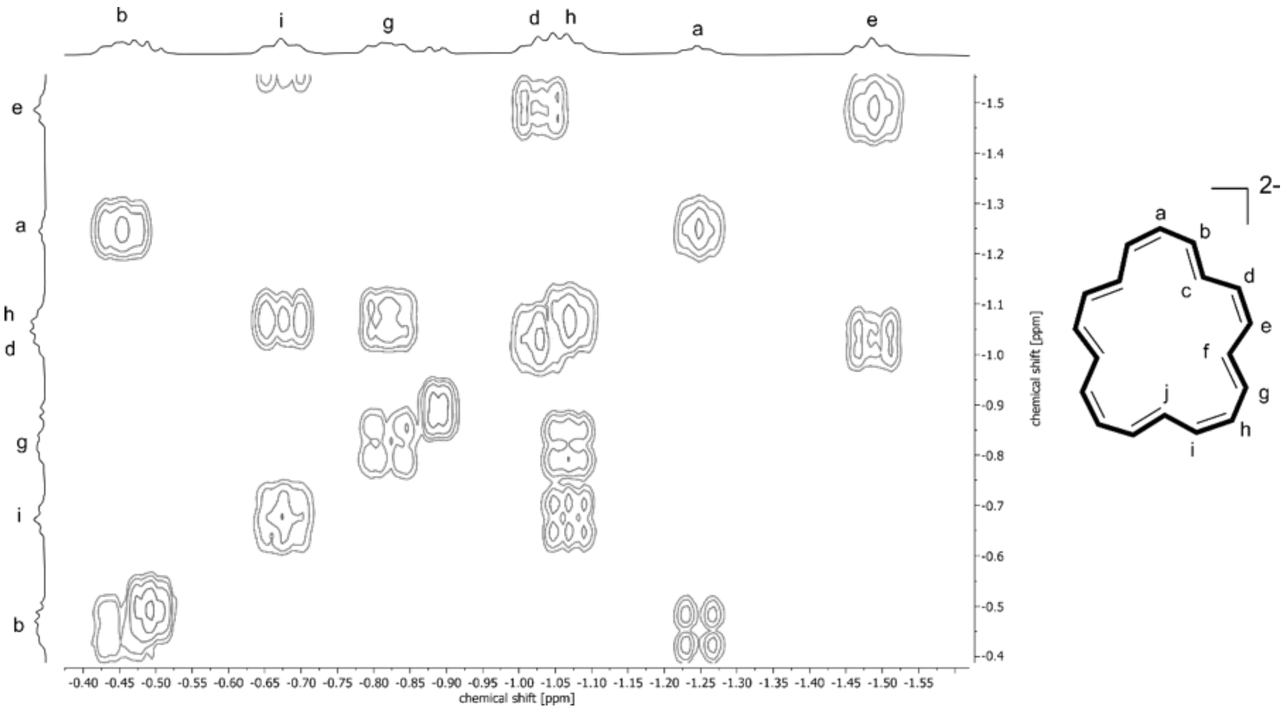

Supplementary Figure 22. Fragment of a ^{1}H-^{1}H COSY spectrum of in-situ generated di-anion **1·K₂**, THF-d_8, –70 °C, 500 MHz.

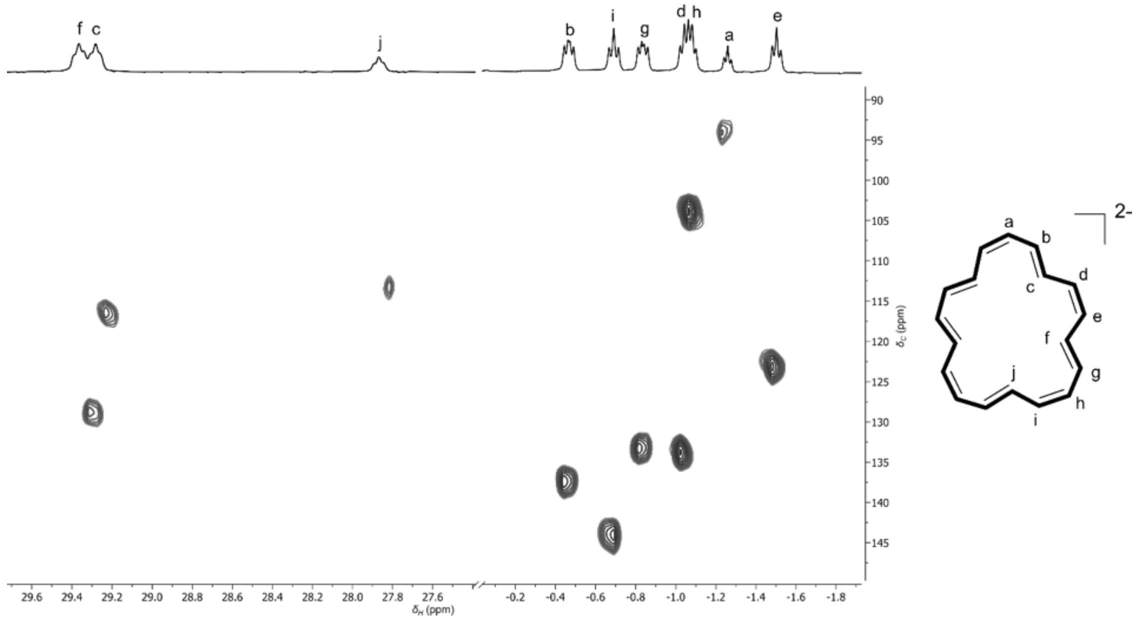

Supplementary Figure 23. Fragments of a ^{1}H-^{13}C HSQC spectrum of in-situ generated di-anion **1·K$_2$**, THF-d_8, –70 °C, 500 MHz.

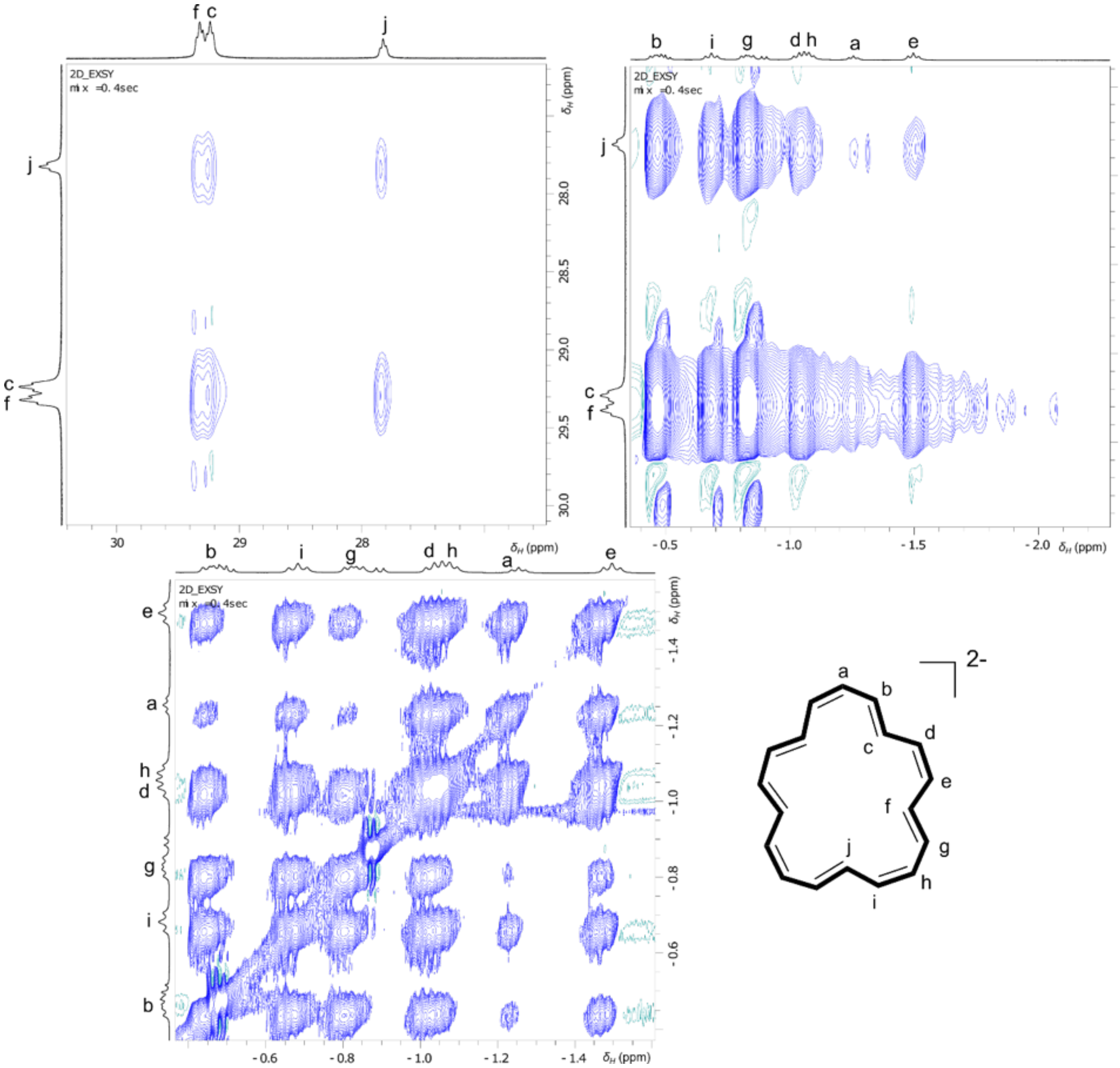

Supplementary Figure 24. Fragments of ^{1}H-^{1}H EXSY spectra of in-situ generated di-anion **1·K$_2$**, mixing time 0.4 sec, THF-d_8, –70 °C, 500 MHz.

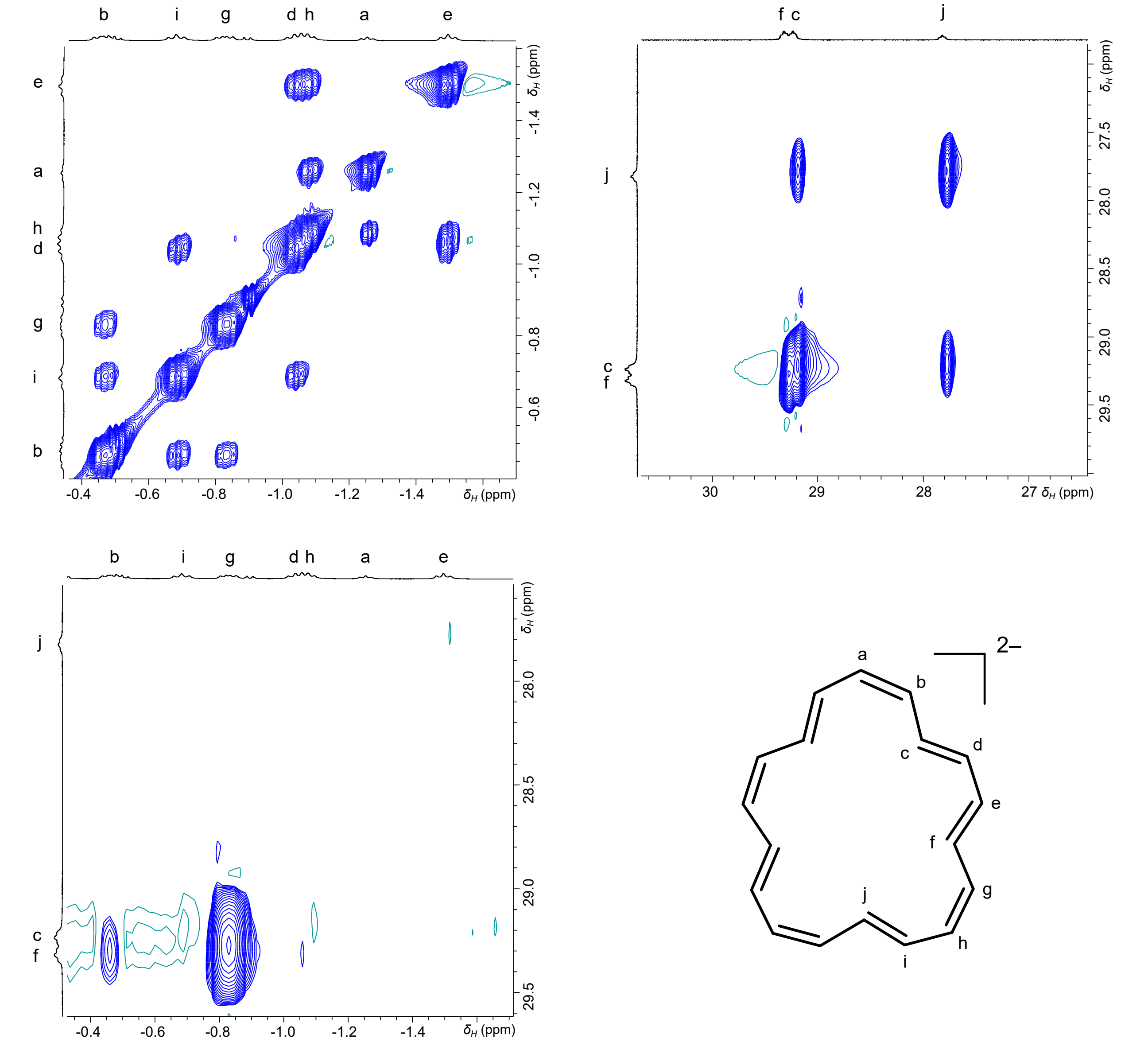

Supplementary Figure 25. Fragments of ^{1}H-^{1}H EXSY spectra of in-situ generated di-anion **1·K$_2$**, mixing time 0.0 sec, THF-d_8, –70 °C, 500 MHz.

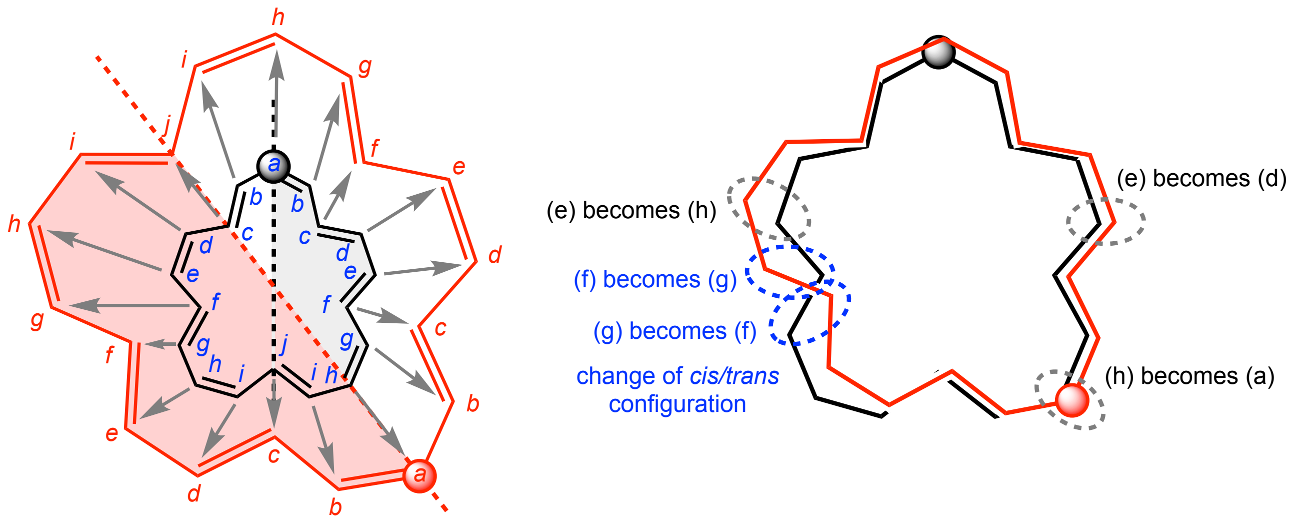

Supplementary Figure 26. Visualization of the exchange processes in **1·K$_2$** based on the observed ^{1}H-^{1}H EXSY NMR correlations observed with 0.0 sec mixing time.

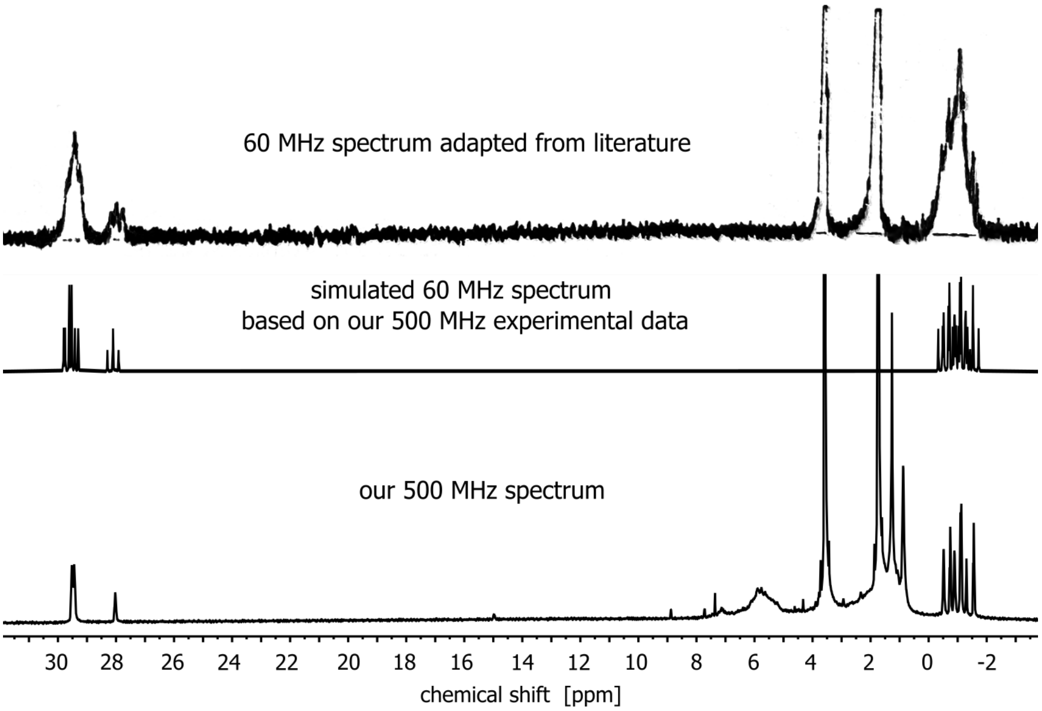

Supplementary Figure 27. Comparison of the reported 60 MHz ^{1}H NMR spectrum (–110 °C),[13] our 500 MHz spectrum (–70 °C) and simulated 60 MHz spectrum based on our experimental data, of the in-situ generated di-anion **1·K$_2$**.

5.3 Monitoring of reaction of **1** with Li metal

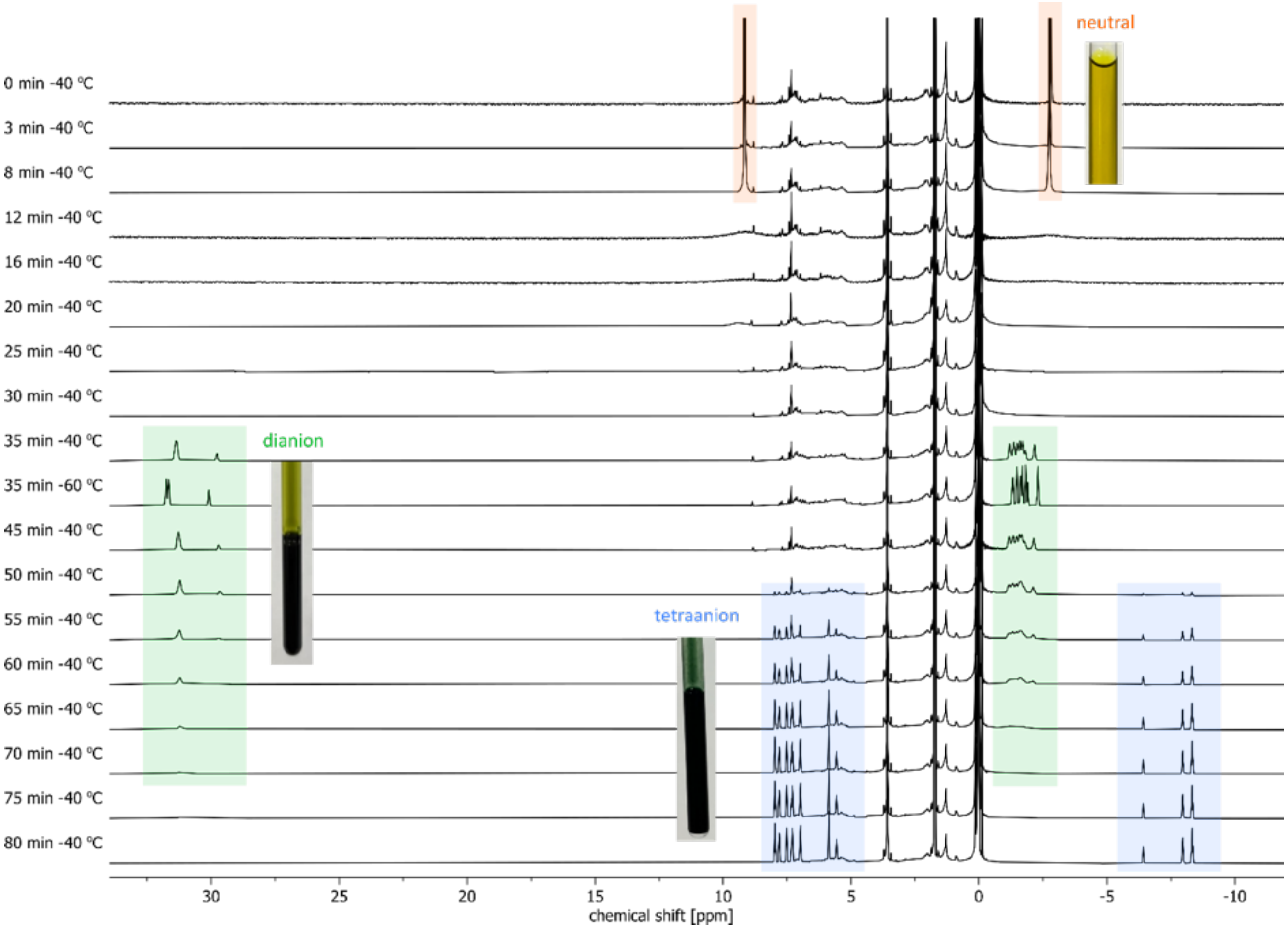

Supplementary Figure 28. ^{1}H NMR monitoring of reaction between **1** and lithium metal, THF-d_8, 500 MHz. The sample was shaken with excess of lithium metal at 20 °C.

5.4 Lithium salt of [18]annulene di-anion **1·Li₂**

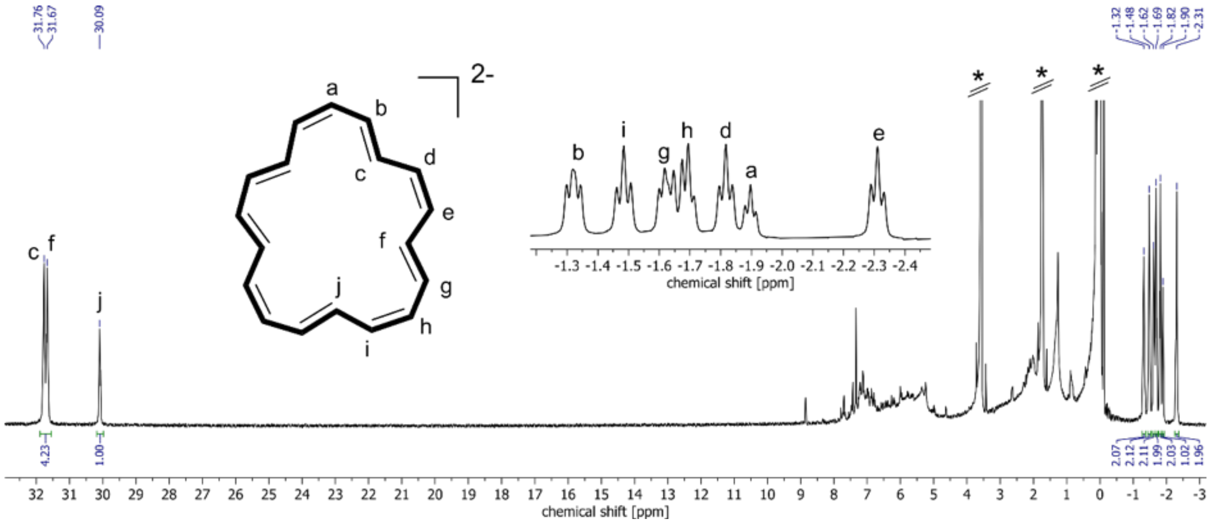

Figure 29. ^{1}H NMR spectrum of in-situ generated di-anion **1·Li₂**, THF-d_8, –60 °C, 500 MHz.

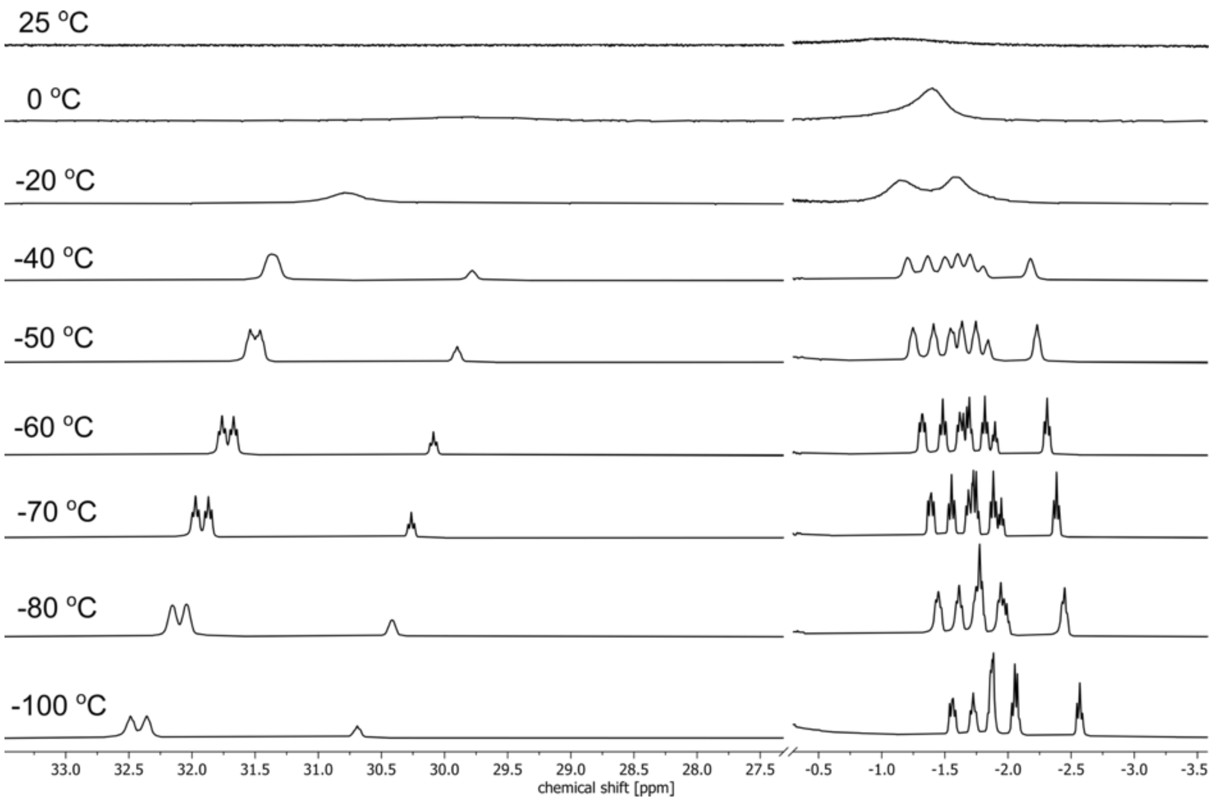

Supplementary Figure 30. Fragments of VT NMR spectra of in-situ generated di-anion **1·Li₂**, THF-d_8, 500 MHz.

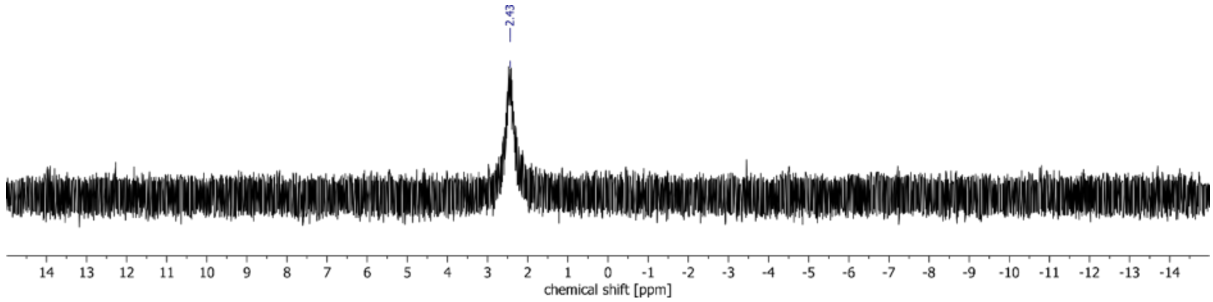

Supplementary Figure 31. ^{7}Li NMR spectrum of in-situ generated di-anion **1·Li₂**. THF-d_8, –60 °C, 194 MHz.

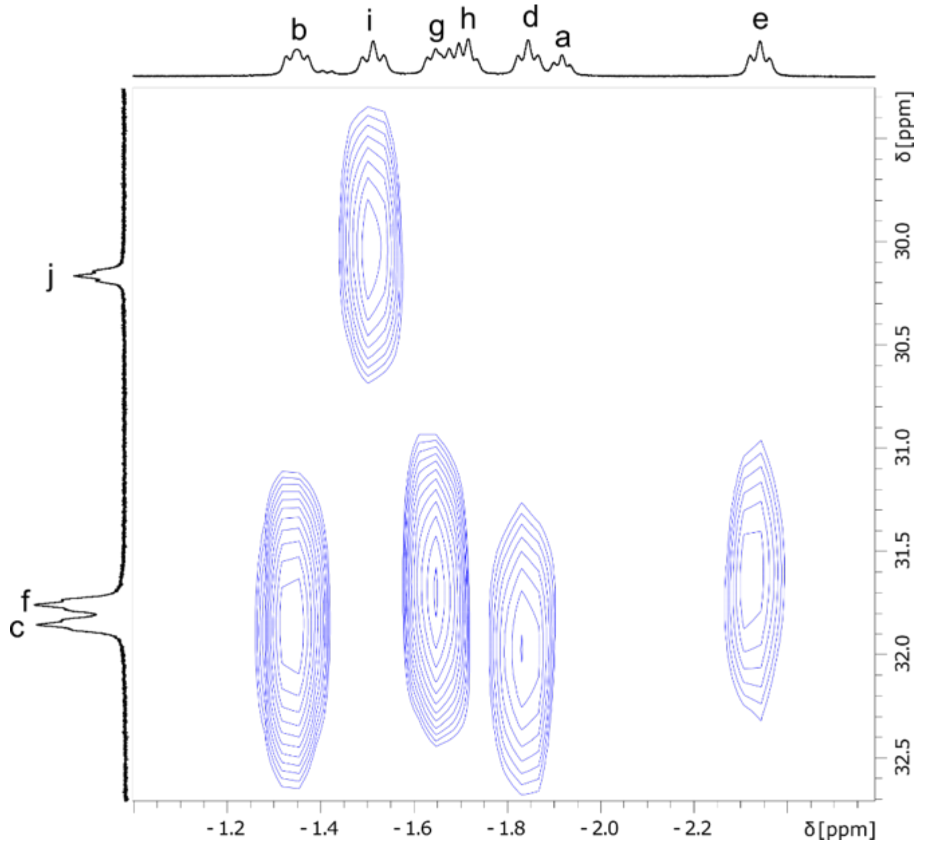

Supplementary Figure 32. Fragment of a ^{1}H-^{1}H COSY spectrum of in-situ generated di-anion **1·Li$_2$**, THF-d_8, –60 °C, 500 MHz.

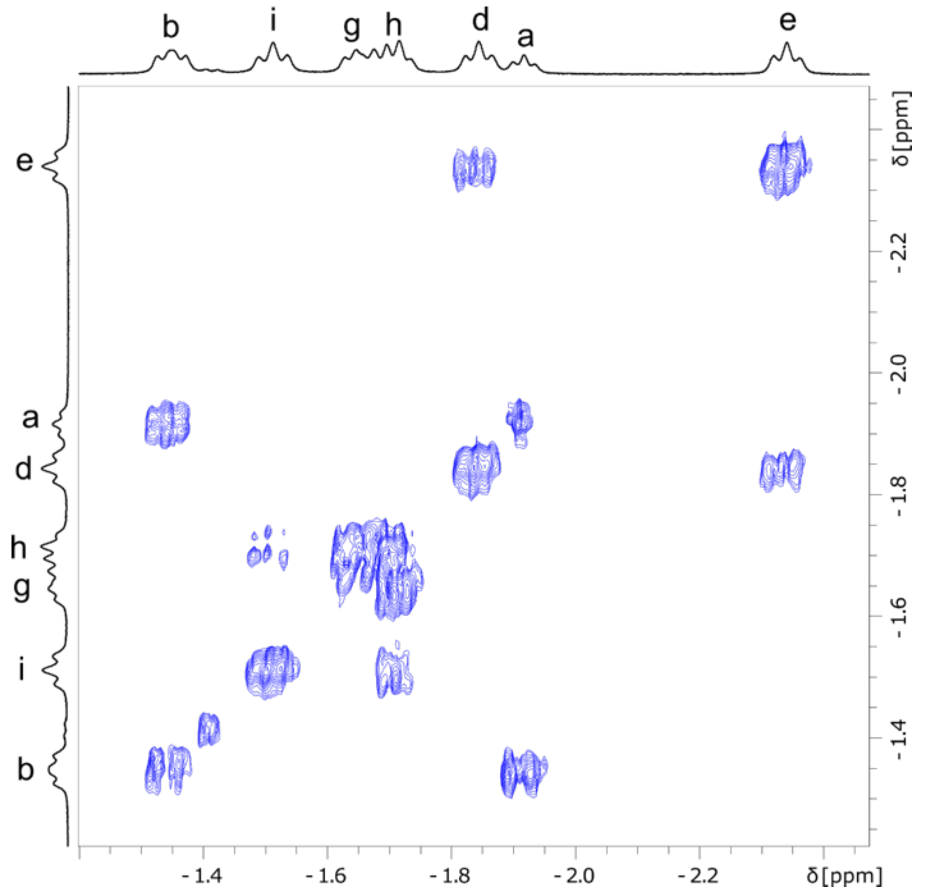

Supplementary Figure 33. Fragment of a ^{1}H-^{1}H COSY spectrum of in-situ generated **1·Li$_2$**, THF-d_8, –60 °C, 500 MHz.

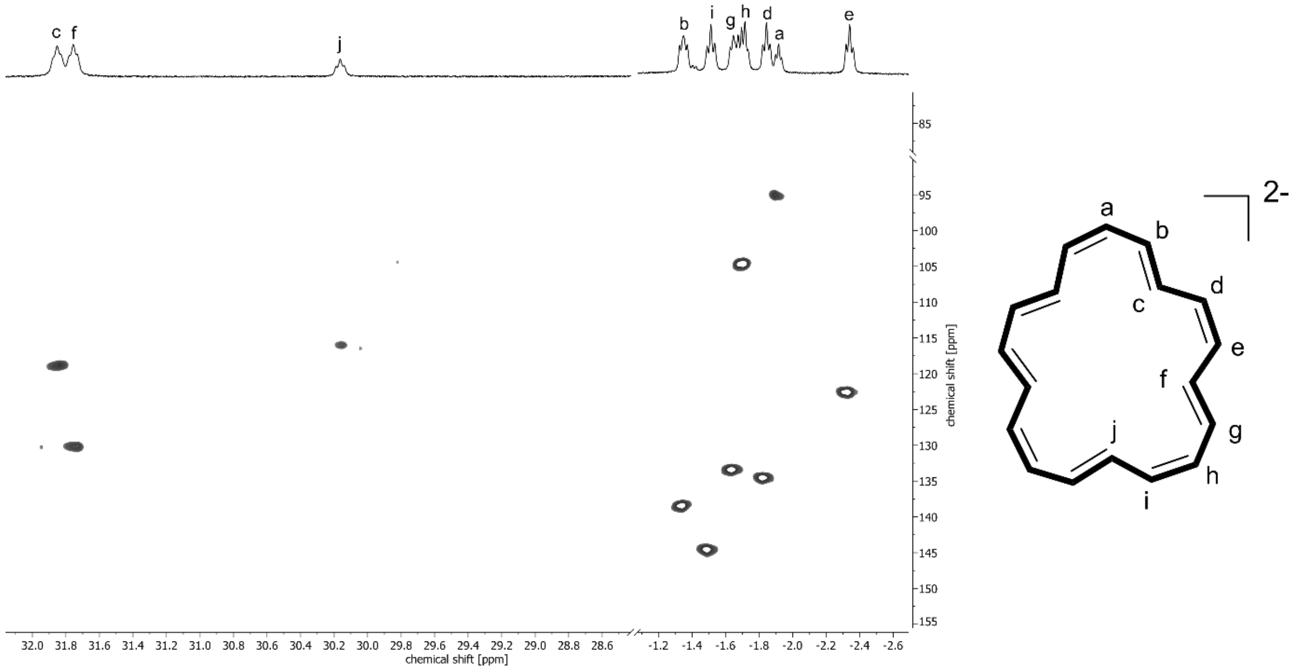

Supplementary Figure 34. Fragment of a ^{1}H-^{13}C HSQC spectrum of in-situ generated di-anion **1·Li₂**, THF-d_8, –60 °C, 500 MHz.

5.5 [18]Annulene tetra-anion $\mathbf{1_2{\cdot}Li_8}$

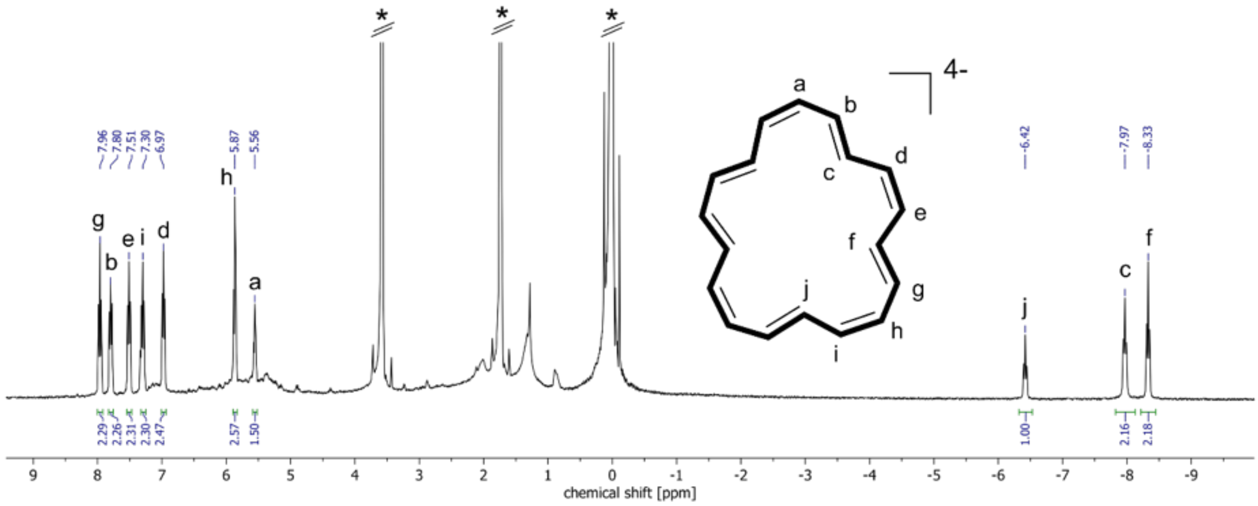

Supplementary Figure 35. ^{1}H NMR spectrum of in-situ generated tetra-anion $\mathbf{1_2{\cdot}Li_8}$, THF-d_8, –40 °C, 500 MHz.

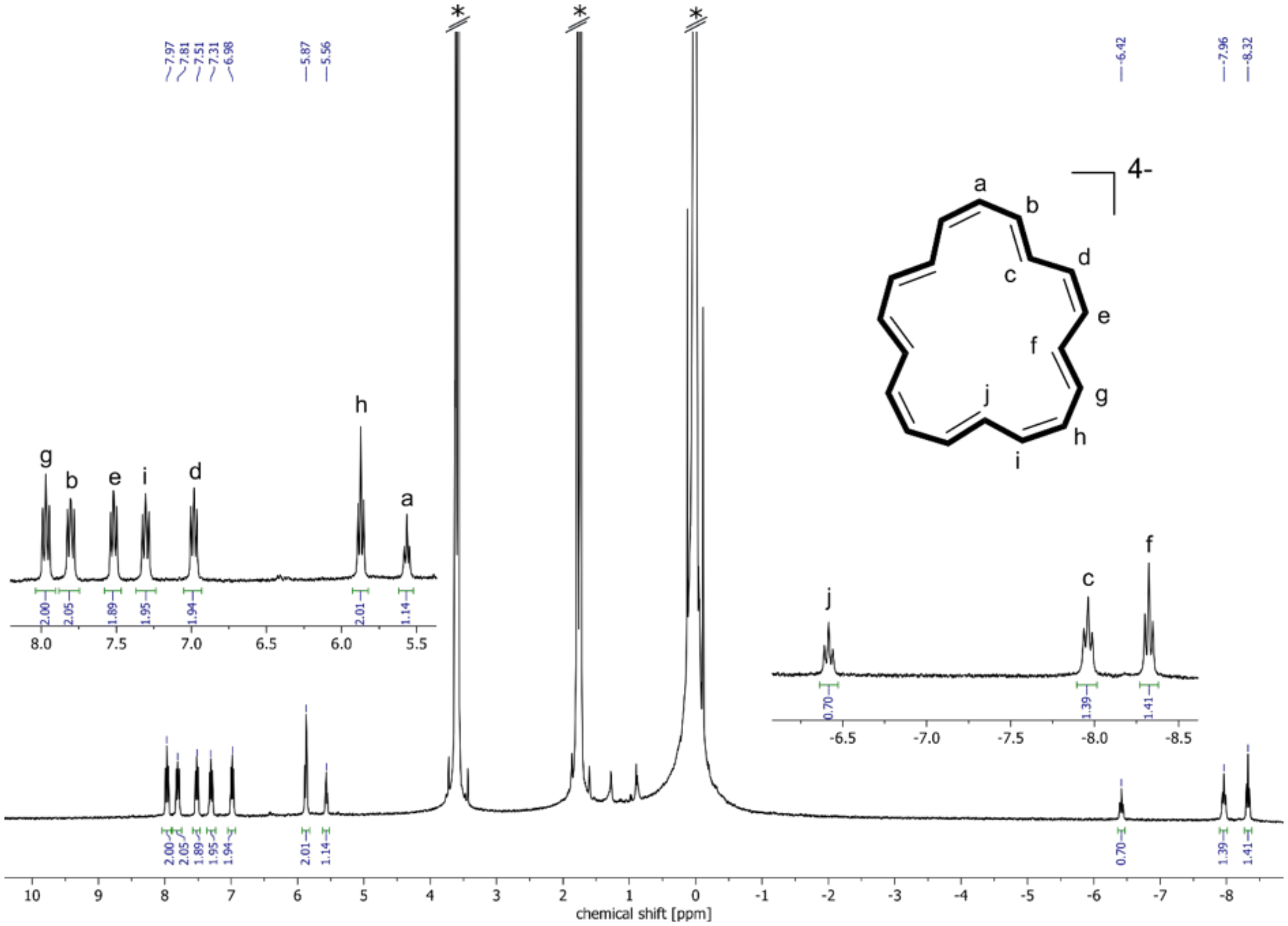

Supplementary Figure 36. ^{1}H NMR spectrum of dissolved crystals of tetra-anion **1_2·Li_8**, THF-d_8, –40 °C, 500 MHz. Asterisks indicate residual THF solvent and TMS signals.

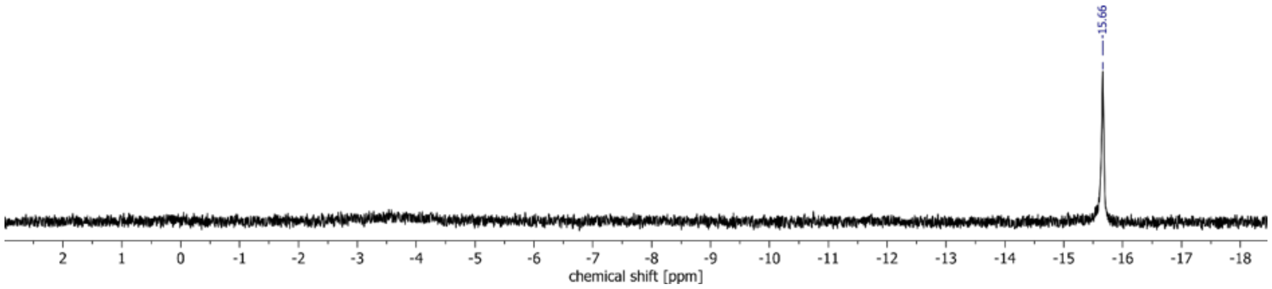

Supplementary Figure 37. ^{7}Li NMR spectrum of dissolved crystals of in-situ generated tetra-anion **1_2·Li_8**, THF-d_8, –40 °C, 500 MHz.

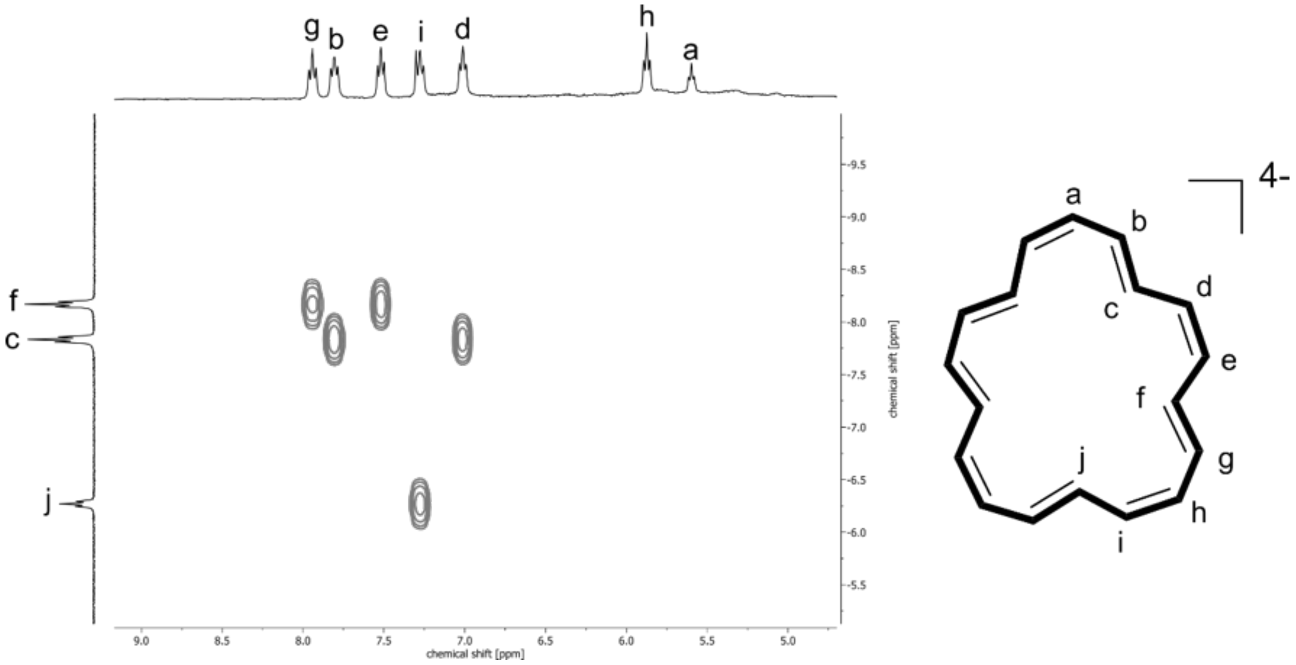

Supplementary Figure 38. Fragment of a ^{1}H-^{1}H COSY spectrum of in-situ generated tetra-anion **1_2·Li$_8$**, THF-d_8, 25 °C, 500 MHz.

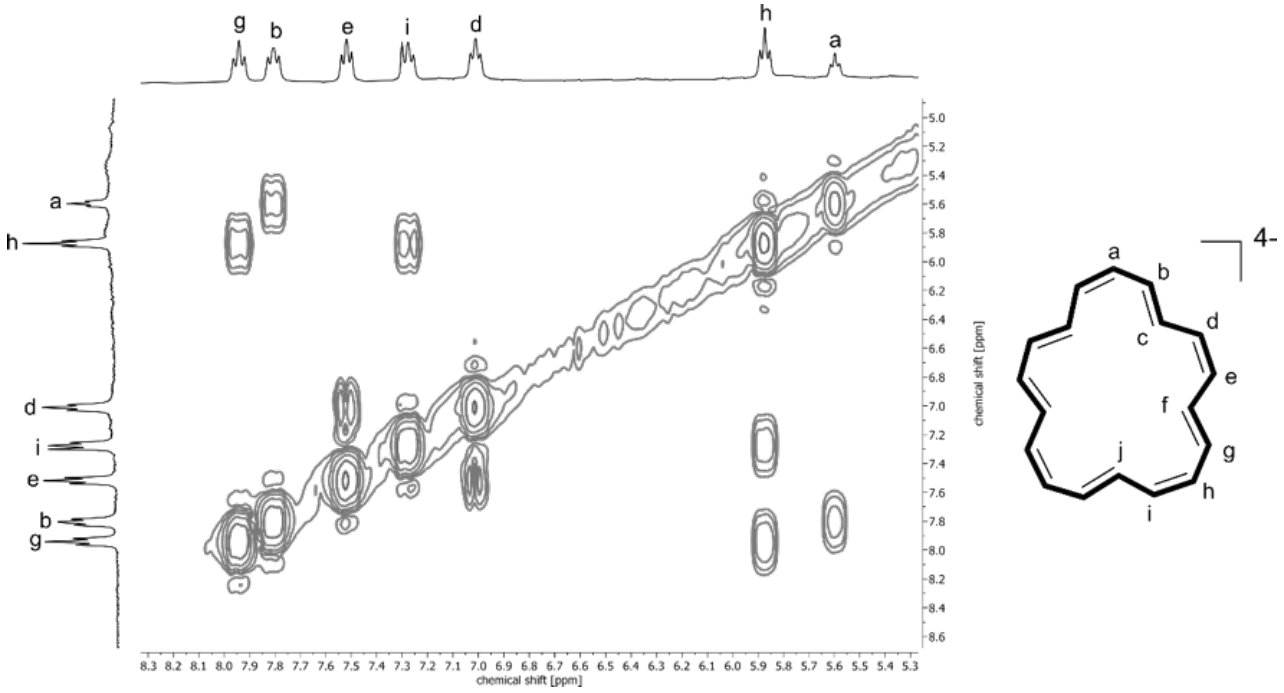

Supplementary Figure 39. Fragment of a ^{1}H-^{1}H COSY spectrum of in-situ generated tetra-anion **1_2·Li$_8$**, THF-d_8, 25 °C, 500 MHz.

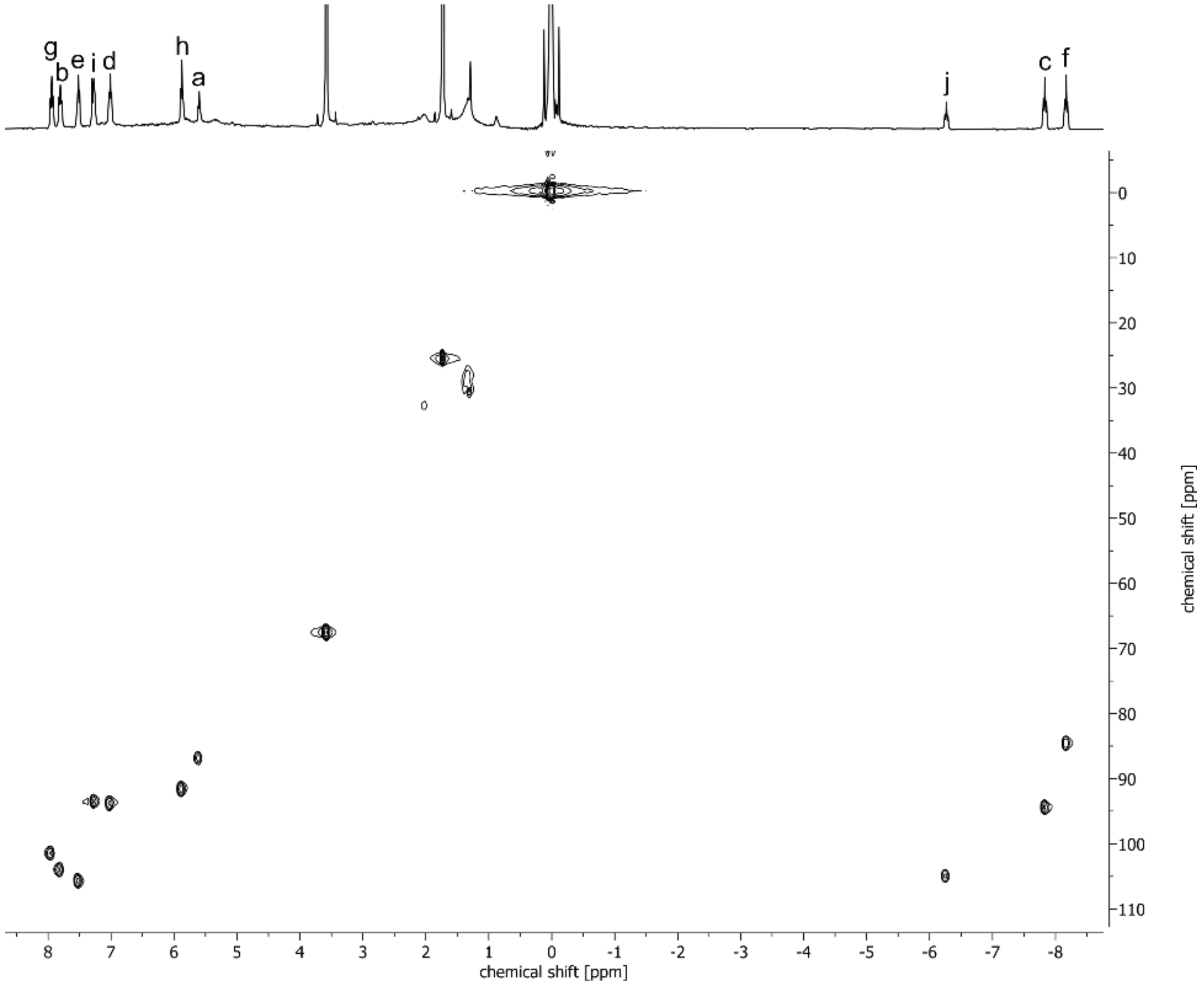

Supplementary Figure 40. ^1H-^{13}C HSQC spectrum of in-situ generated tetra-anion $\mathbf{1_2{\cdot}Li_8}$, THF-d_8, 25 °C, 500 MHz.

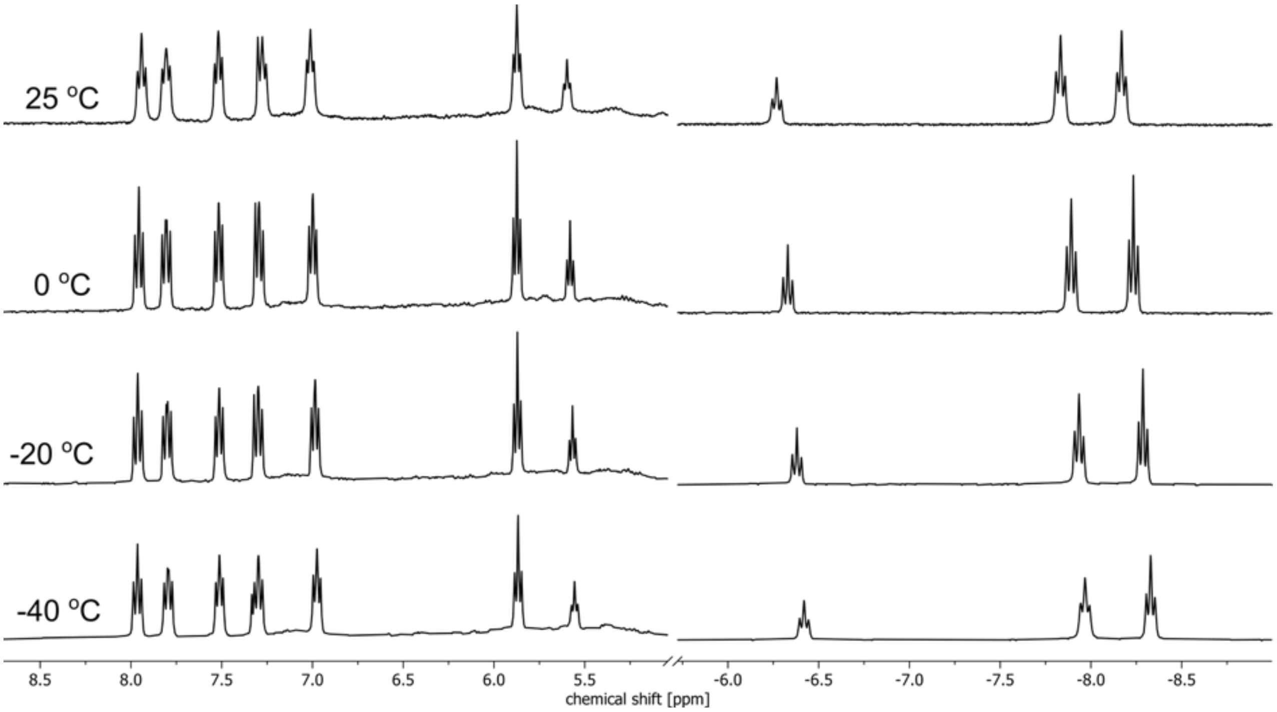

Supplementary Figure 41. Fragments of VT NMR spectra of in-situ generated tetra-anion $\mathbf{1_2{\cdot}Li_8}$, THF-d_8, 500 MHz.

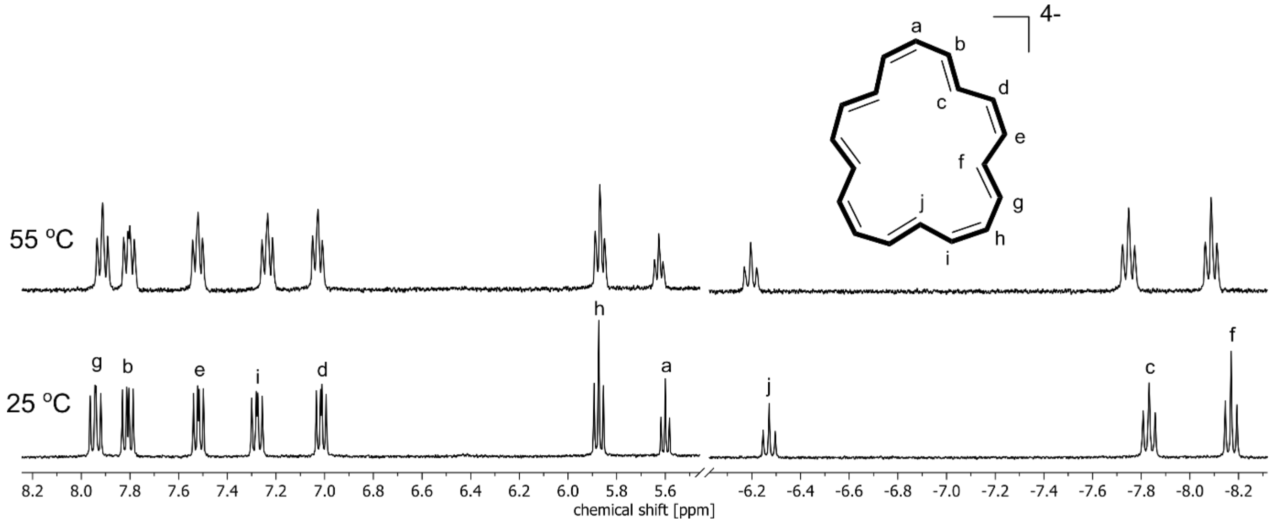

Supplementary Figure 42. Fragments of VT NMR spectra of dissolved crystals of tetra-anion $\mathbf{1_2{\cdot}Li_8}$, THF-d_8, 500 MHz.

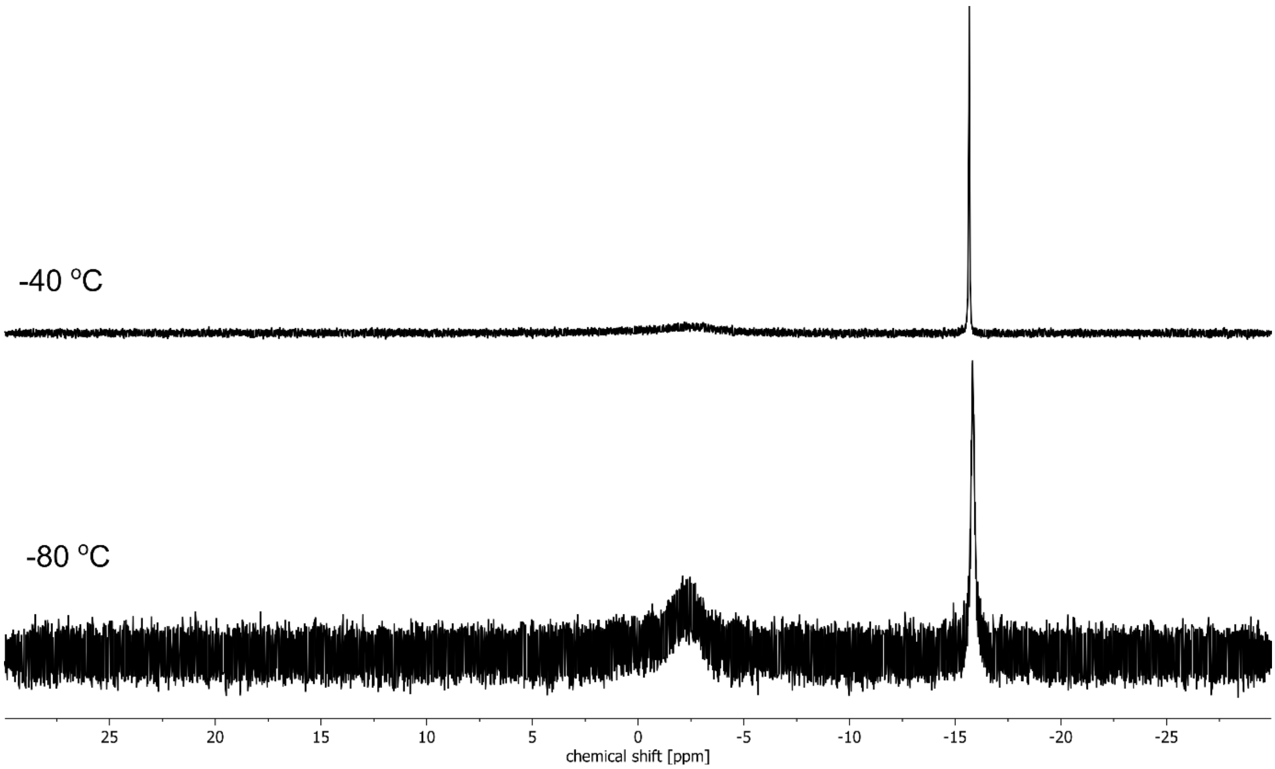

Supplementary Figure 43. ^{7}Li NMR spectra of in-situ generated tetra-anion $\mathbf{1_2{\cdot}Li_8}$ at –40 °C (top) and –80 °C (bottom). THF-d_8, 194 MHz.

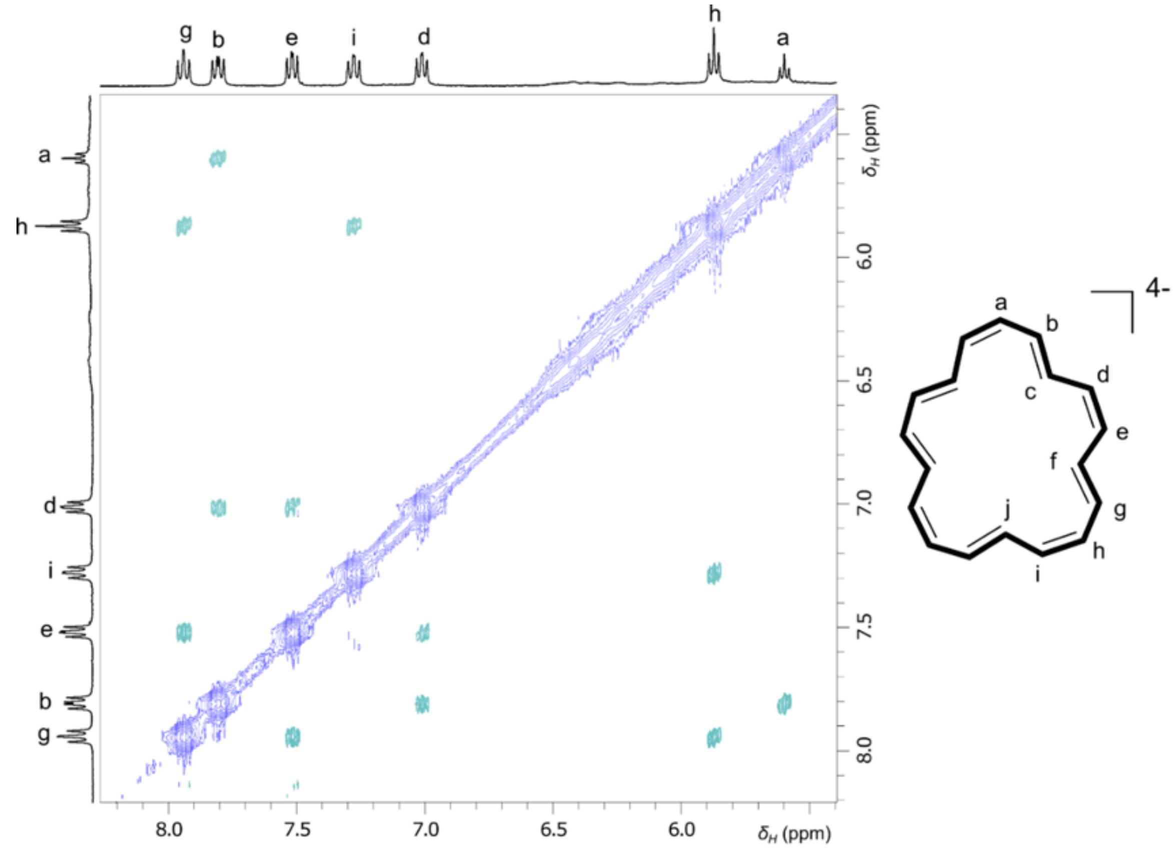

Supplementary Figure 44. Fragment of an ^{1}H-^{1}H EXSY spectrum of in-situ generated $\mathbf{1_2{\cdot}Li_8}$, THF-d_8, 25 °C, 500 MHz, mixing time 0.4 sec. The cross peaks have opposite phase to the diagonal, indicating that they are in fact NOE peaks.

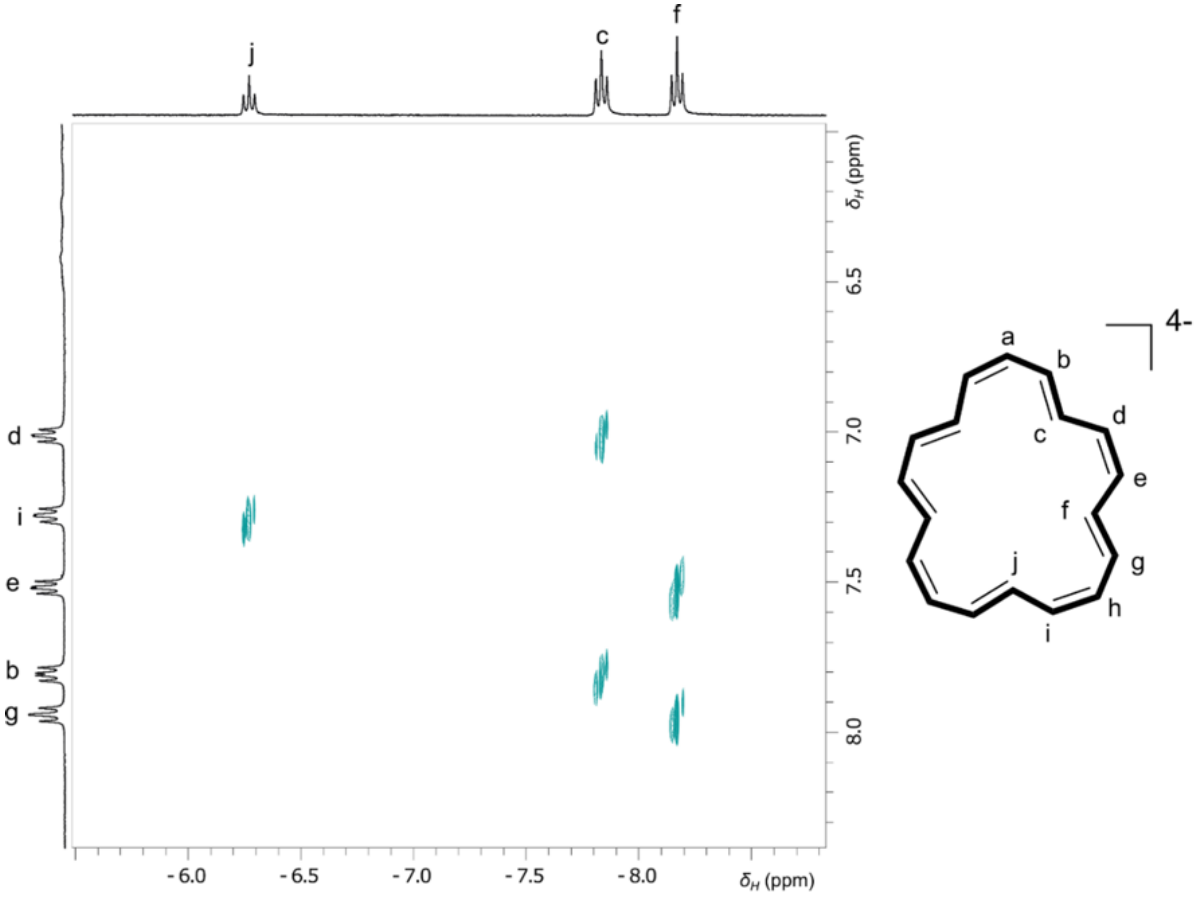

Supplementary Figure 45. Fragment of an ^{1}H-^{1}H EXSY spectrum of in-situ generated $\mathbf{1_2{\cdot}Li_8}$, THF-d_8, 25 °C, 500 MHz, mixing time 0.4 sec. The cross peaks have opposite phase to the diagonal, indicating that they are in fact NOE peaks.

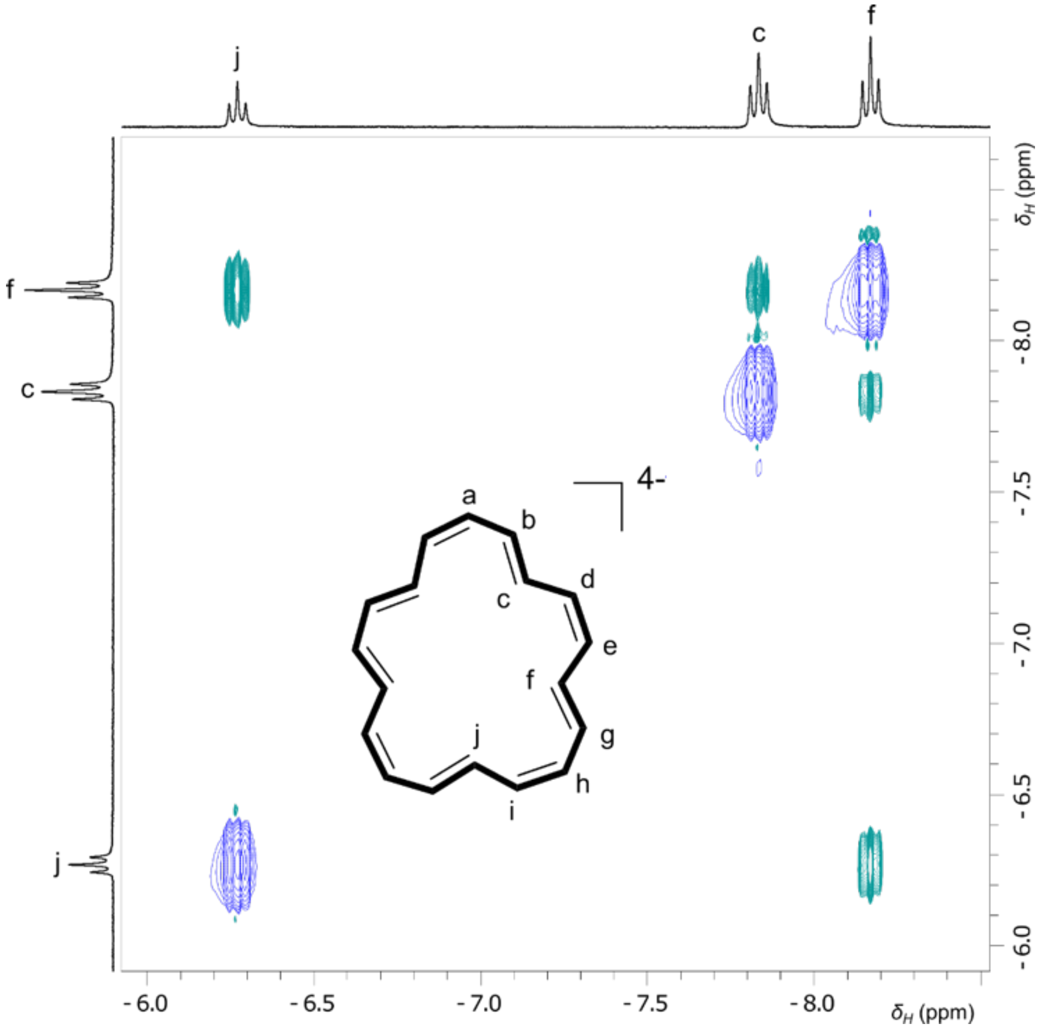

Supplementary Figure 46. Fragment of an ^{1}H-^{1}H EXSY spectrum of in-situ generated $\mathbf{1_2{\cdot}Li_8}$, THF-d_8, 25 °C, 500 MHz, mixing time 0.4 sec. The cross peaks have opposite phase to the diagonal, indicating that they are in fact NOE peaks.

5.6 Heteroleptic sandwich **1·2·Li₈**

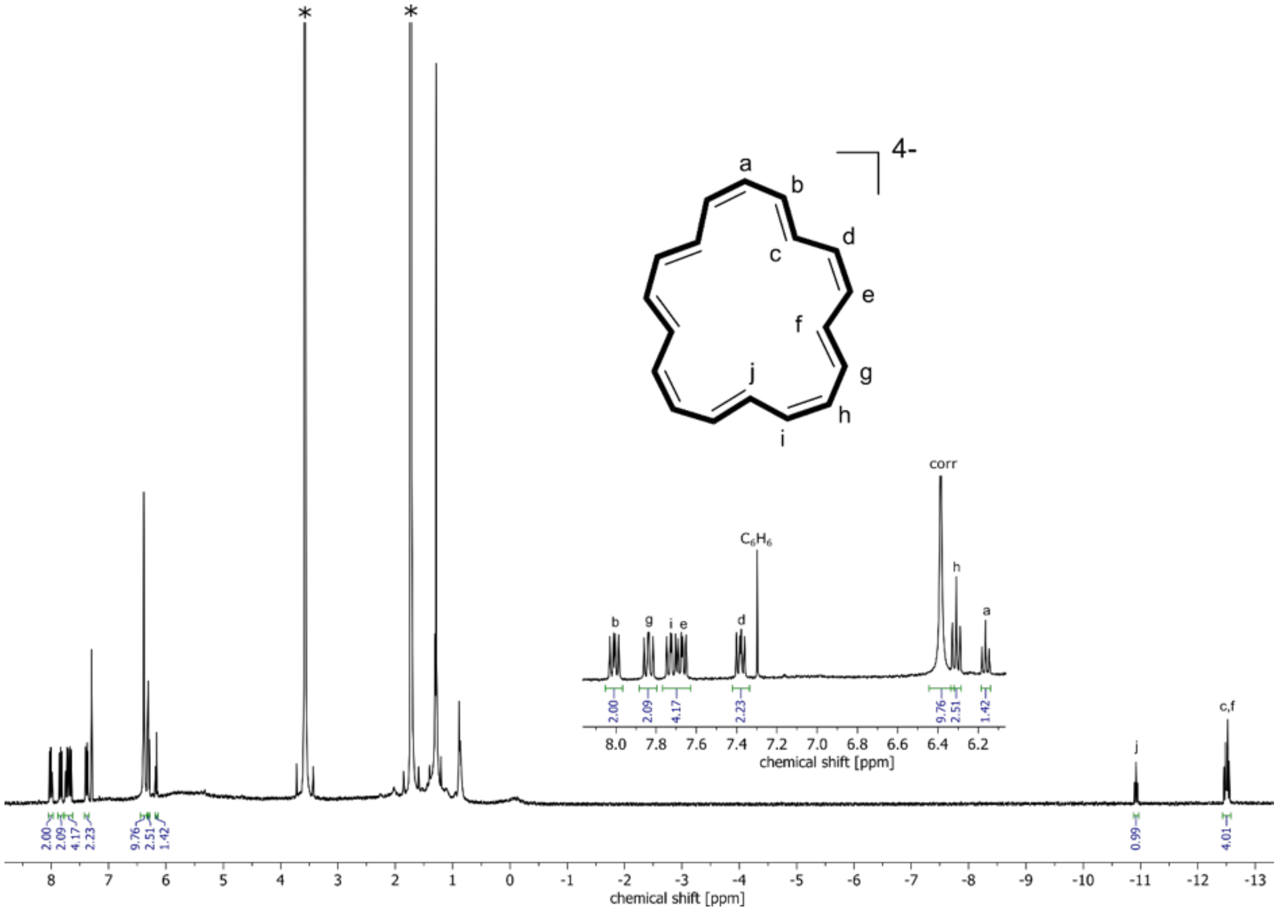

Supplementary Figure 47. Full ^{1}H NMR spectrum of the in-situ generated heteroleptic sandwich **1·2·Li$_8$** together with signals assignment. THF-d_8, 25 °C. 500 MHz. Residual signals of THF are marked with asterisk.

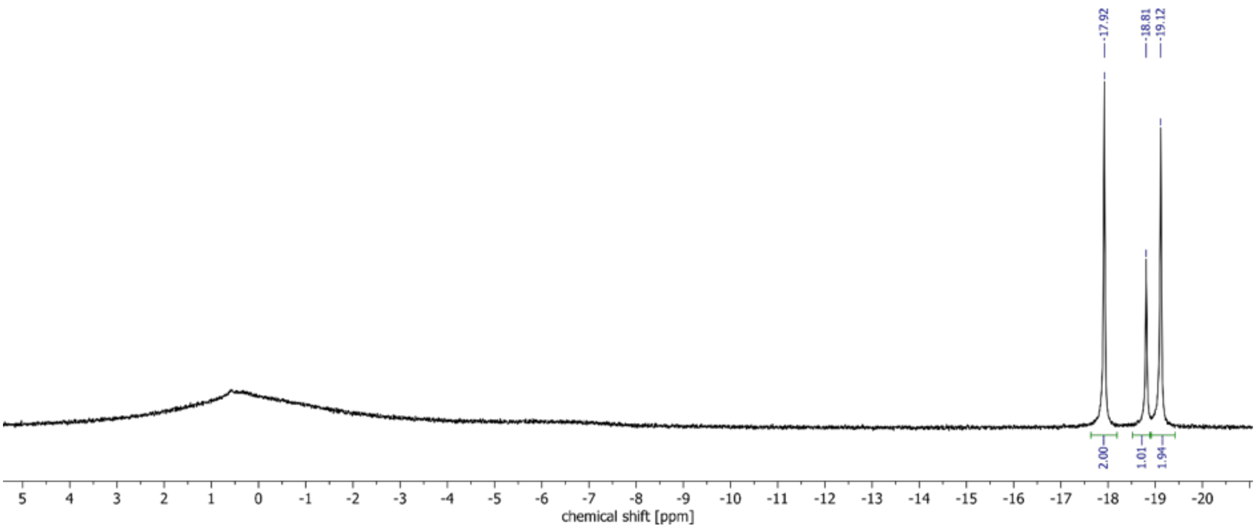

Supplementary Figure 48. ^{7}Li NMR spectrum of the in-situ generated heteroleptic sandwich **1·2·Li$_8$**, THF-d_8, –80 °C. 194 MHz.

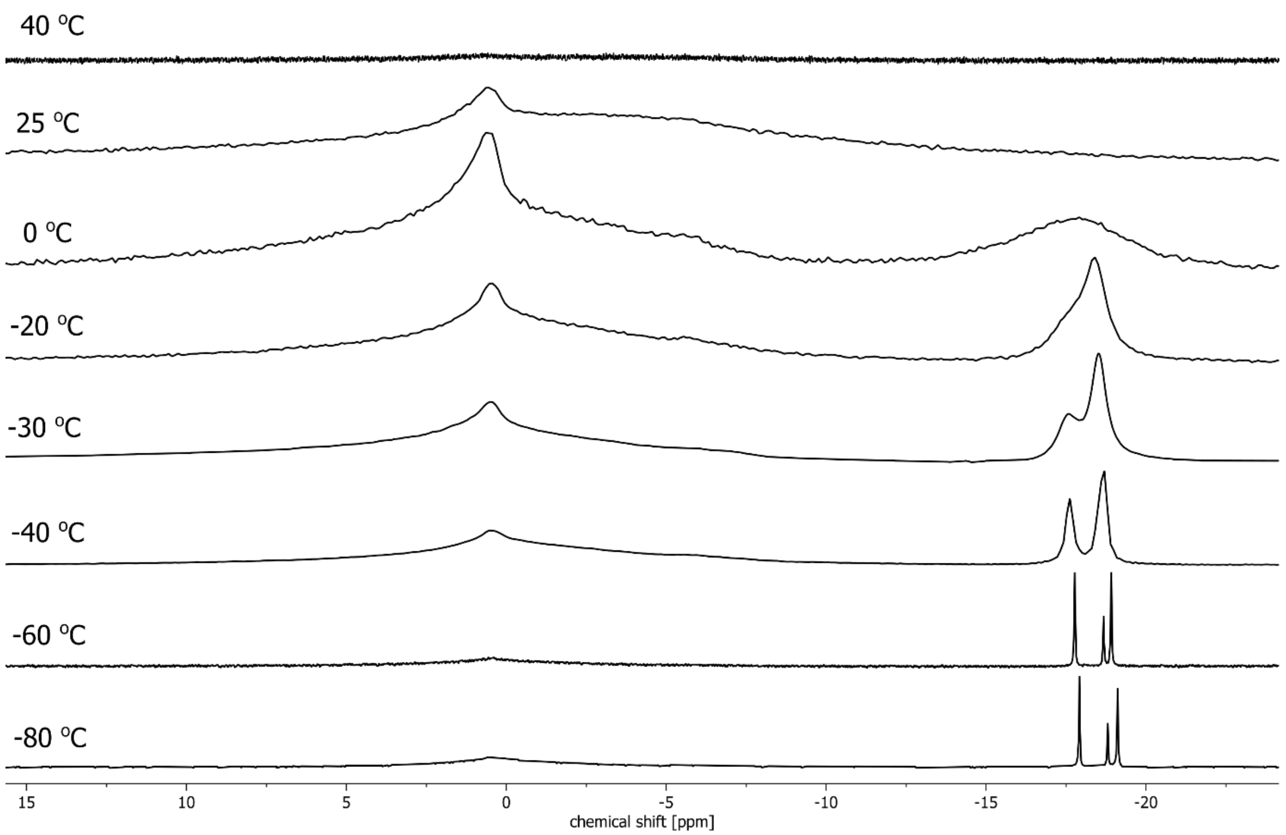

Supplementary Figure 49. Fragments of VT ^{7}Li NMR spectra of the in-situ generated heteroleptic sandwich **1·2·Li$_8$**, THF-d_8, 194 MHz.

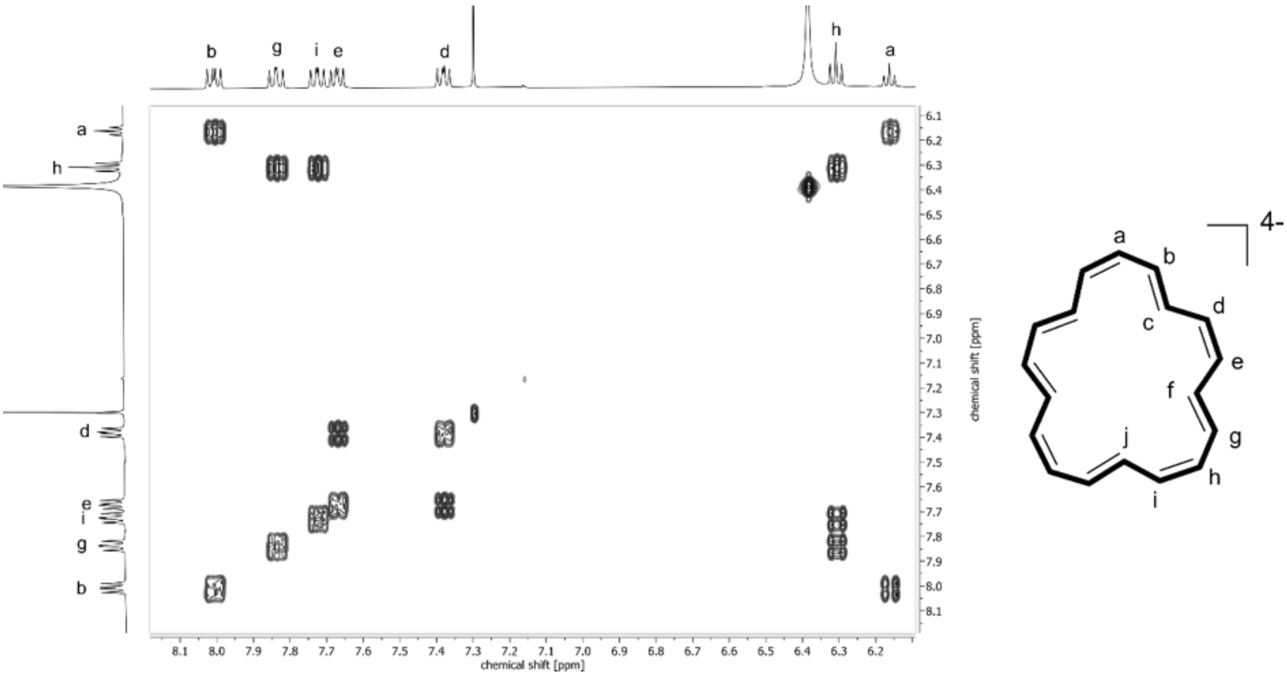

Supplementary Figure 50. Fragment of a ^{1}H-$^{1}COSY$ spectrum of in-situ generated **1·2·Li$_8$**, THF-d_8, 25 °C, 600 MHz.

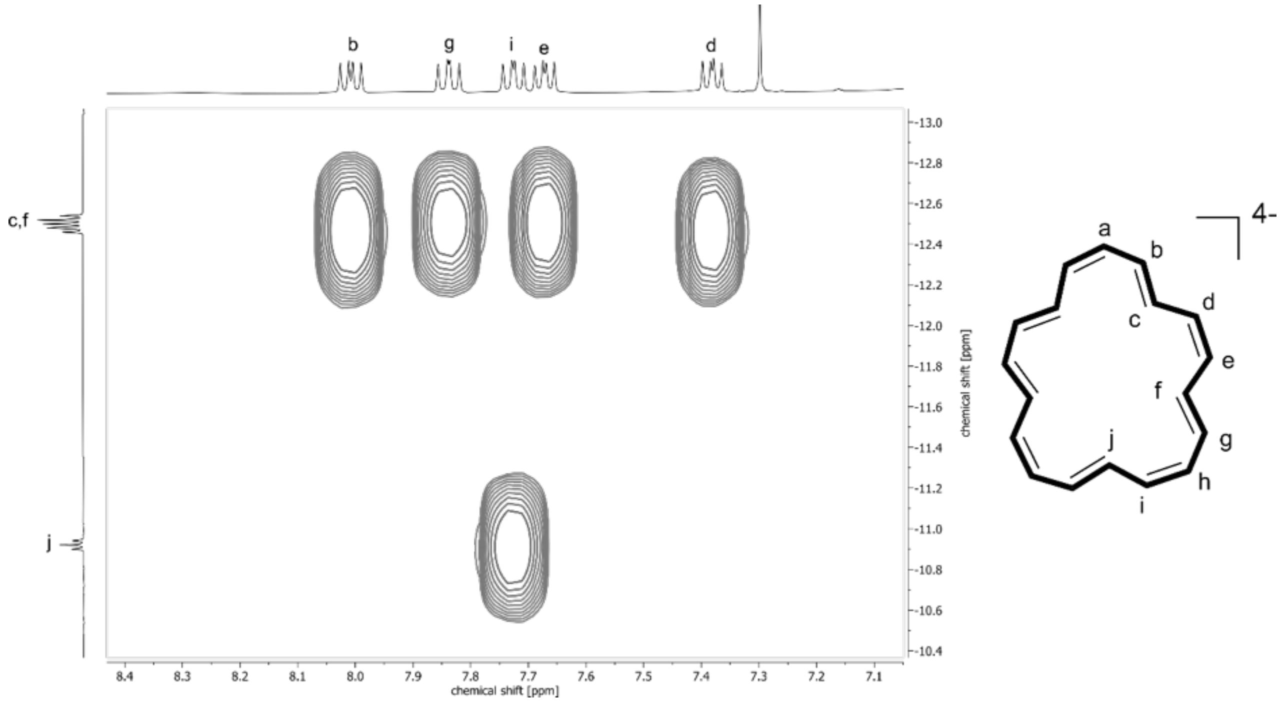

Supplementary Figure 51. Fragment of a ^{1}H-^{1}COSY spectrum of in-situ generated **1·2·Li$_8$**, THF-d_8, 25 °C, 600 MHz.

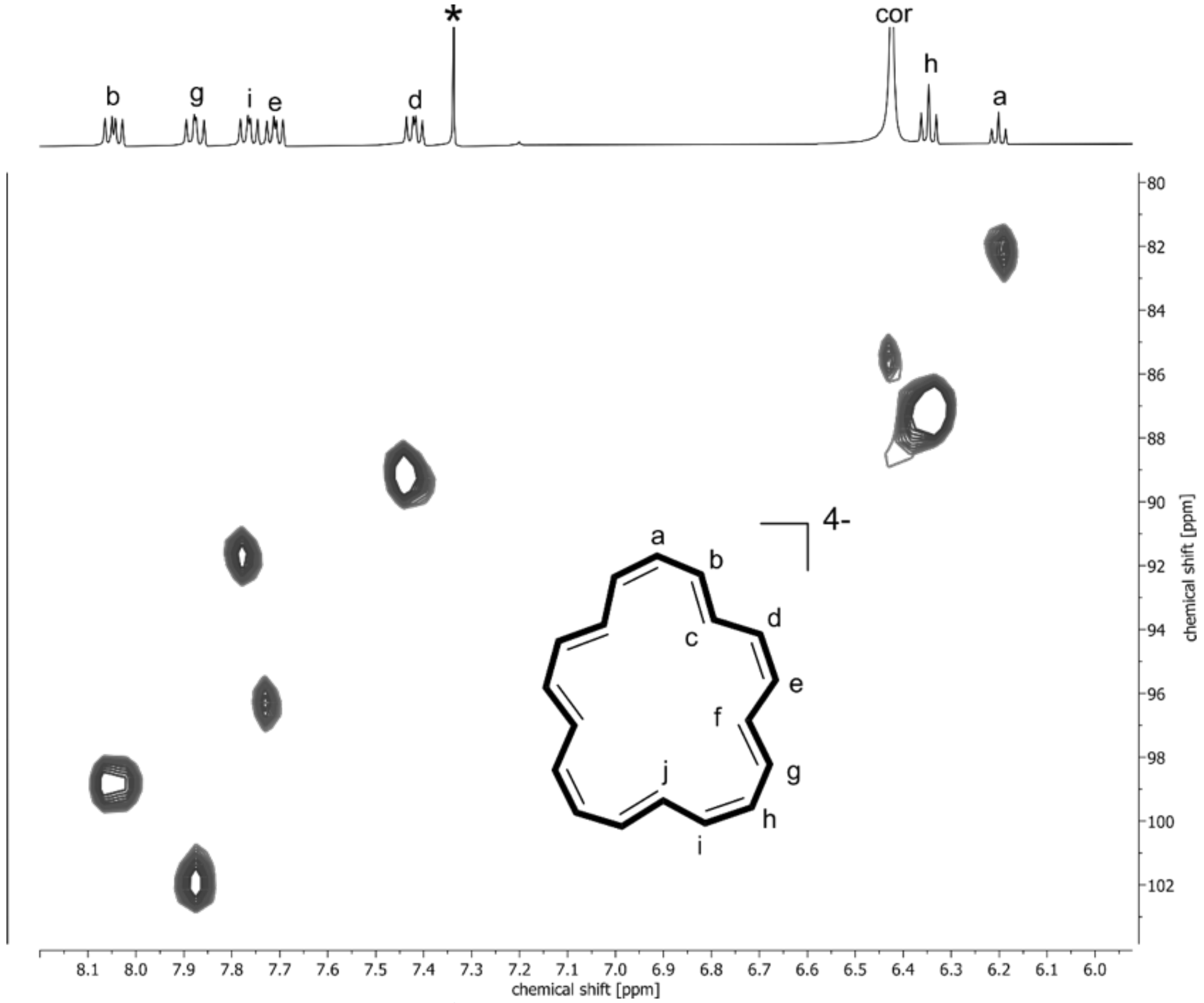

Supplementary Figure 52. Fragment of a ^{1}H-^{13}C HSQC spectrum of in-situ generated **1·2·Li$_8$**, THF-d_8, 25 °C, 600 MHz. Benzene signal is marked with an asterisk.

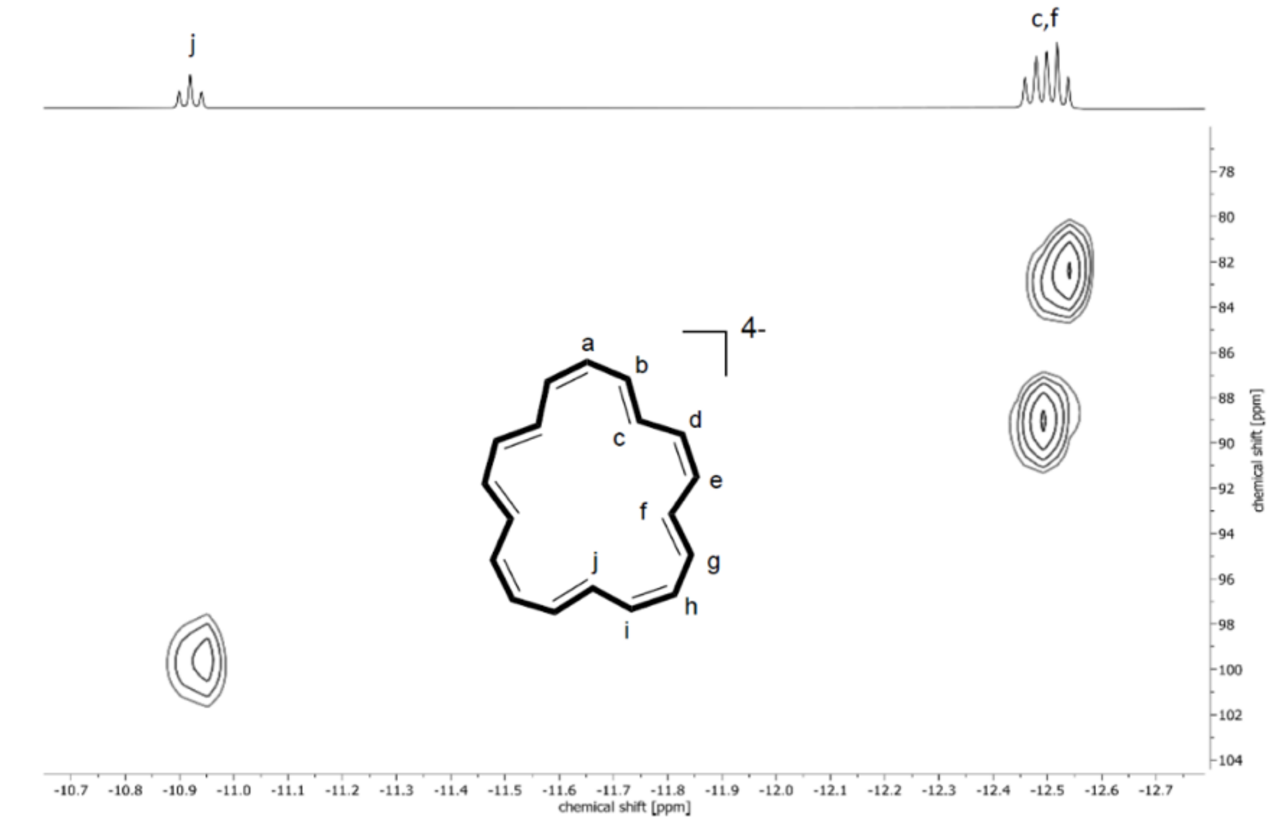

Supplementary Figure 53. Fragment of a ^{1}H-^{13}C HSQC spectrum of in-situ generated **1·2·Li$_8$**, THF-d_8, 25 °C, 600 MHz.

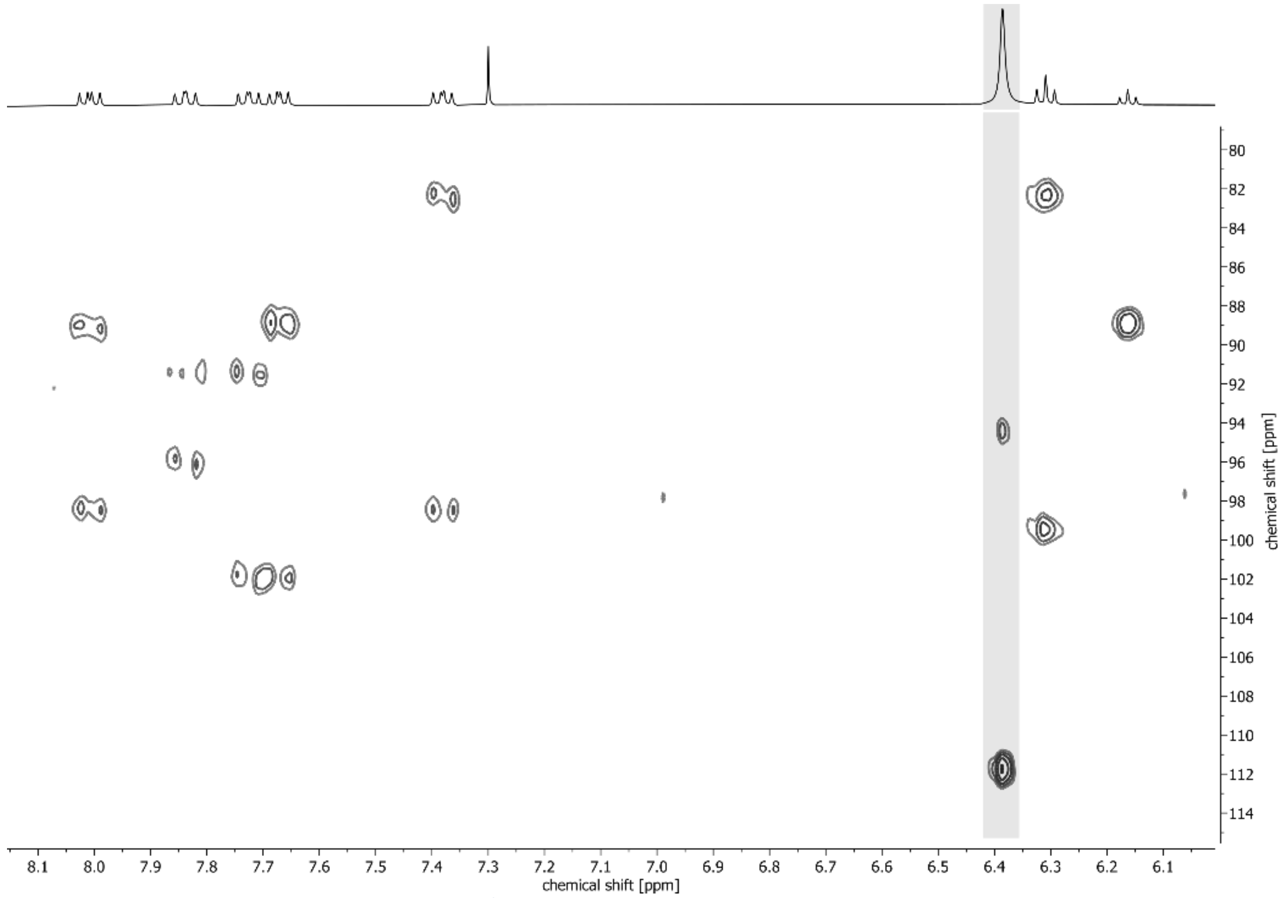

Supplementary Figure 54. Fragment of a ^{1}H-^{13}C HMBC spectrum of in-situ generated **1·2·Li$_8$**, THF-d_8, 25 °C, 600 MHz. Corannulene cross peaks are highlighted in grey.

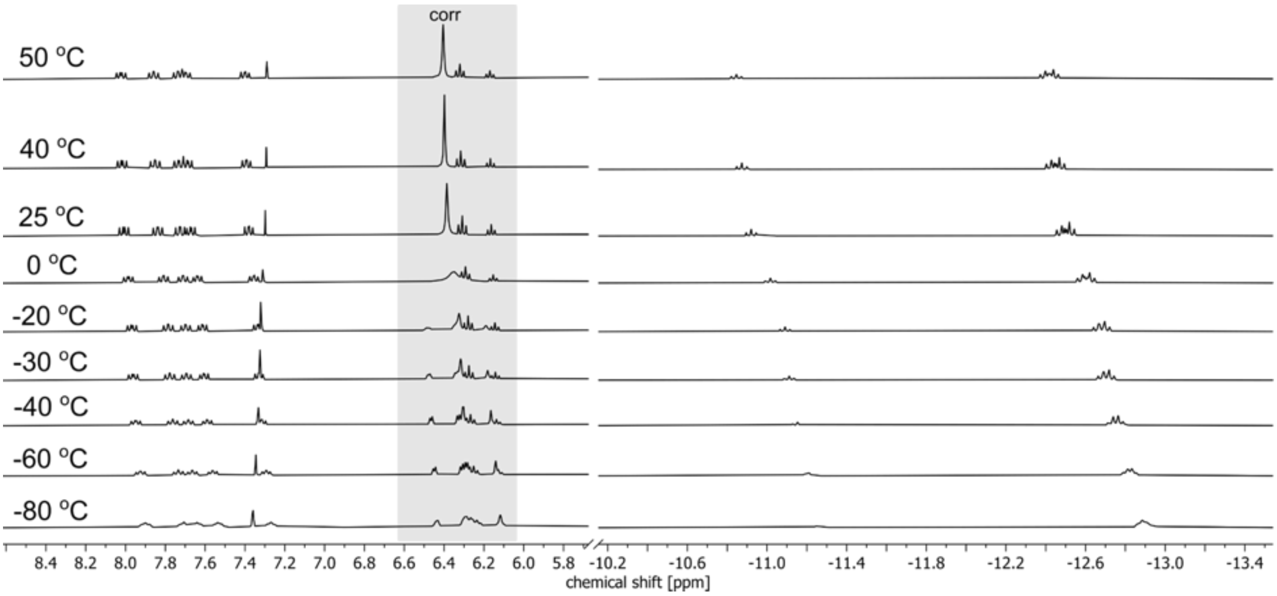

Supplementary Figure 55. VT NMR spectra of in-situ generated heteroleptic sandwich **1·2·Li₈**, THF-d_8, 500 MHz with changes in the corannulene region marked in grey.

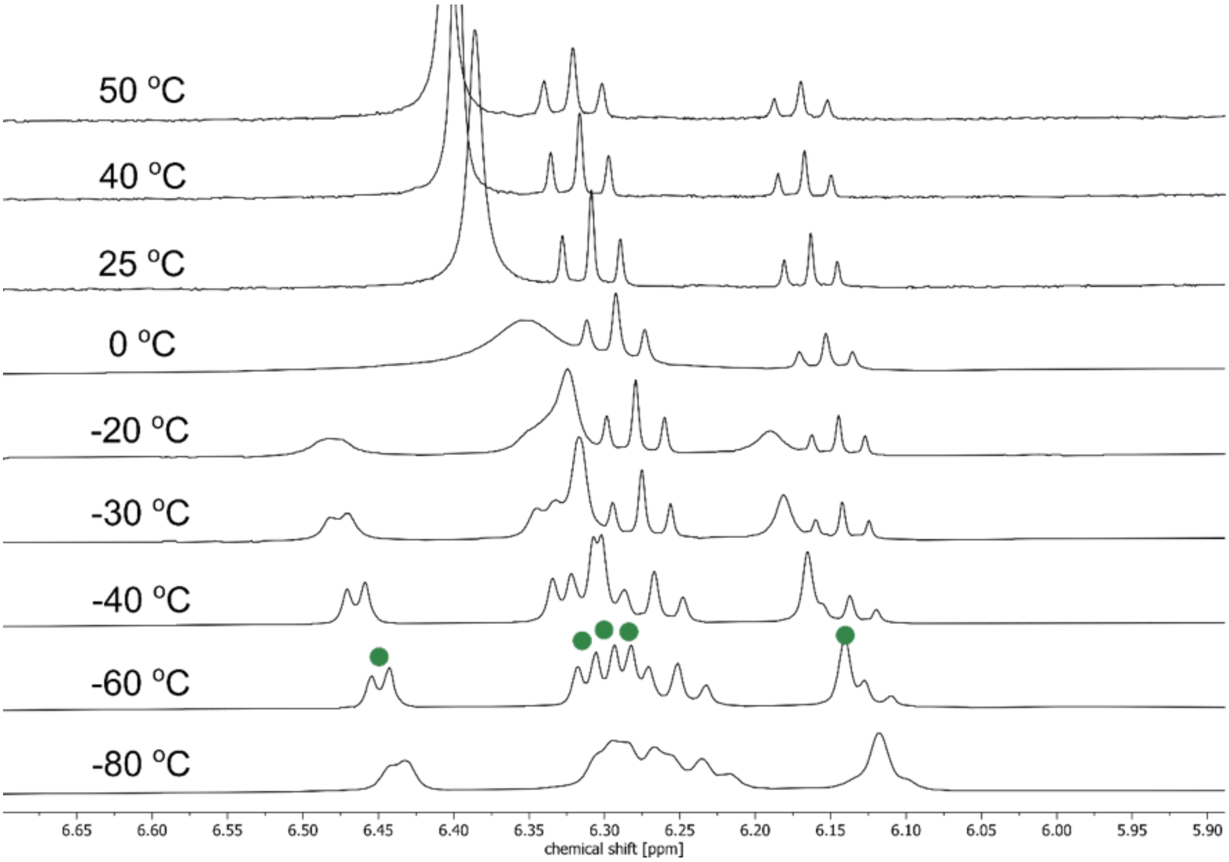

Supplementary Figure 56. VT NMR spectra of the in-situ generated heteroleptic sandwich **1·2·Li₈**, zoom at the corannulene region. THF-d_8, 500 MHz. Signals corresponding to corannulene at low temperature are marked in green.

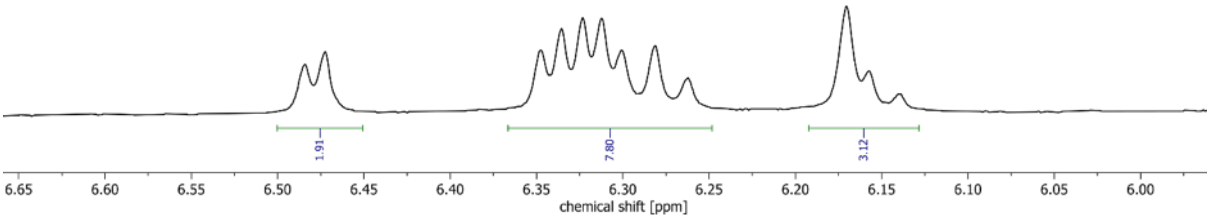

Supplementary Figure 57. Fragment of a ^{1}H NMR spectrum of the in-situ generated heteroleptic sandwich **1·2·Li₈** with integration. THF-d_8, –60 °C, 500 MHz.

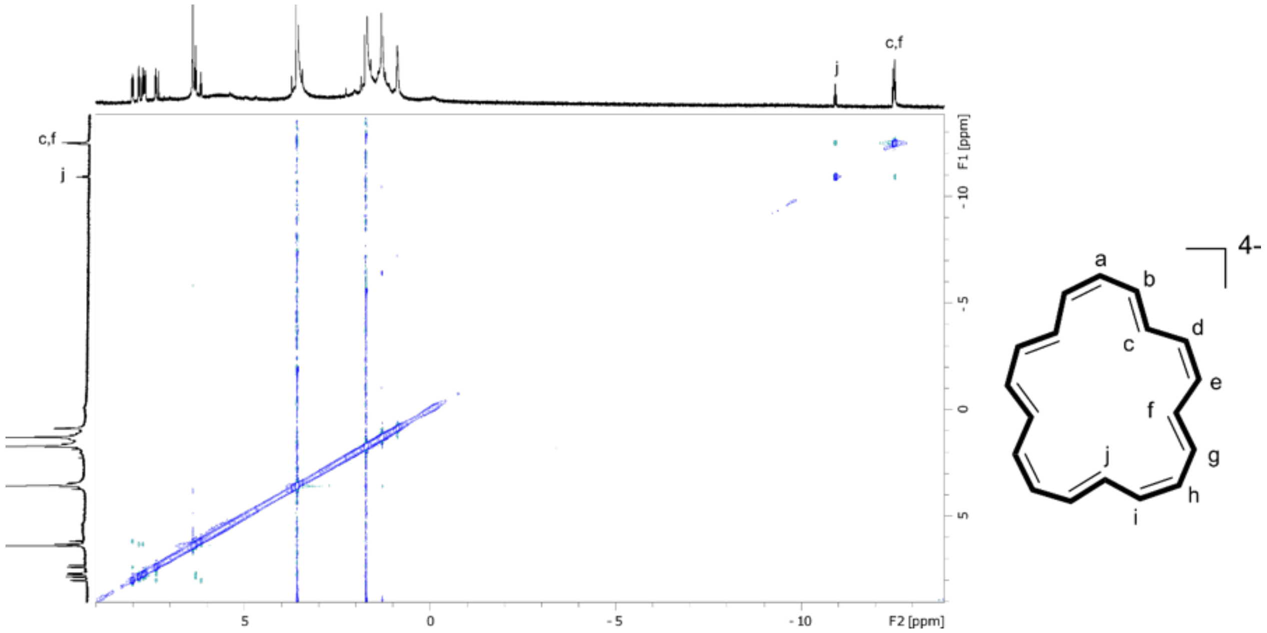

Supplementary Figure 58. ^{1}H-^{1}H EXSY spectrum of in-situ generated **1·2·Li$_8$**, THF-d_8, 25 °C, 500 MHz, mixing time 0.4 sec. The cross peaks have opposite phase to the diagonal, indicating that they are in fact NOE peaks.

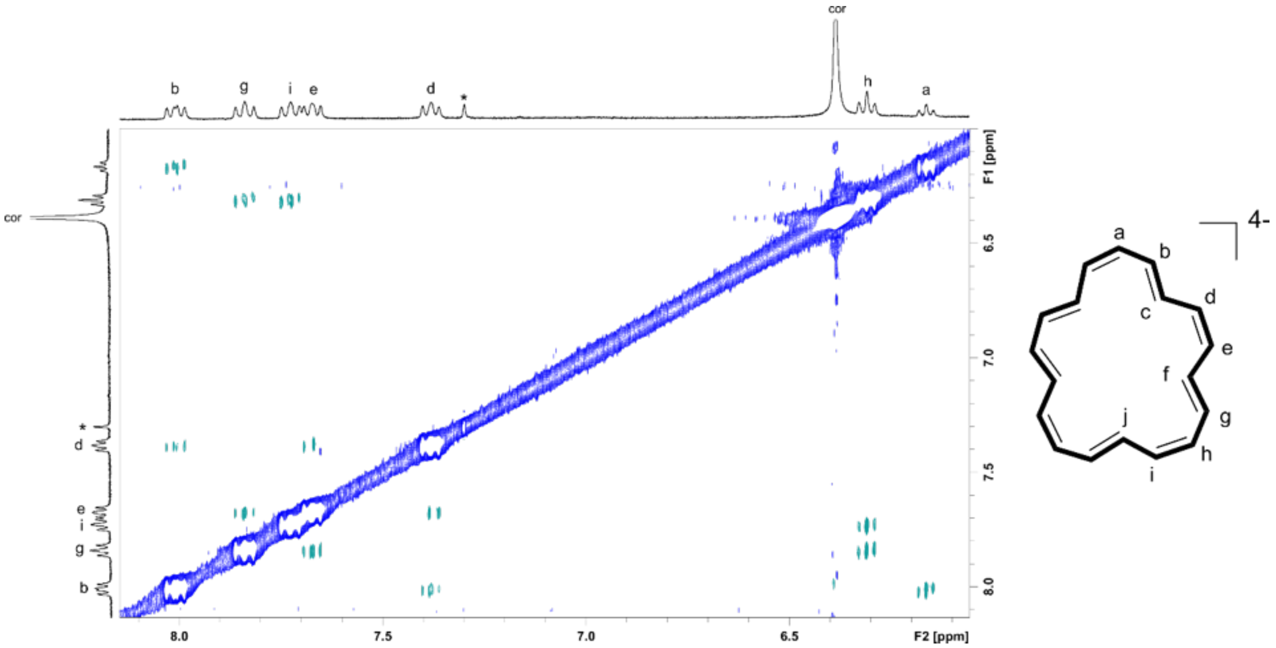

Supplementary Figure 59. Fragment of a ^{1}H-^{1}H EXSY spectrum of in-situ generated **1·2·Li$_8$**, THF-d_8, 25 °C, 500 MHz, mixing time 0.4 sec. The cross peaks have opposite phase to the diagonal, indicating that they are in fact NOE peaks. Asterisk indicates signal of benzene.

Molar ratio **1**:**2** = 1:2

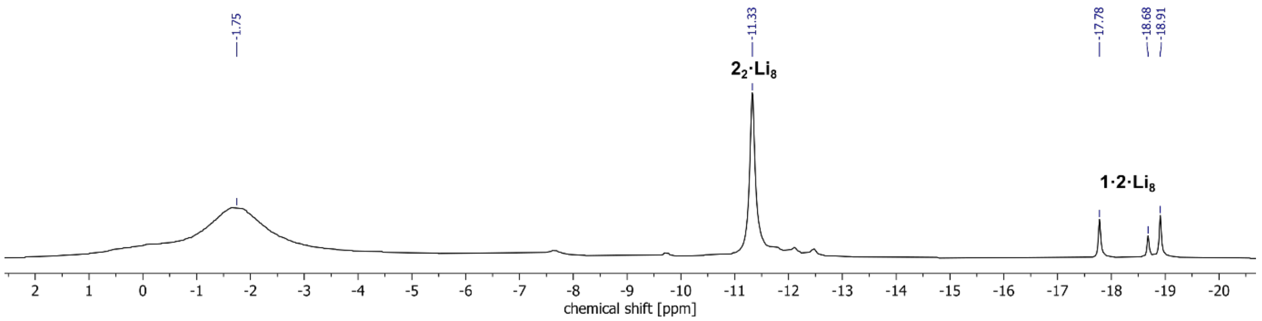

Supplementary Figure 60. ^{7}Li NMR spectrum obtained after in-situ reducing a 2:1 mixture of **1** and **2**. THF-d_8, –60 °C, 194 MHz. Inner Li signals are assigned to particular sandwiched structures. When [18]annulene **1** and corannulene **2** were mixed in 2:1 or 1:2 molar ratio with excess of lithium metal, formation of heteroleptic sandwich was observed together with the homo-sandwich of a hydrocarbon which was used in excess.

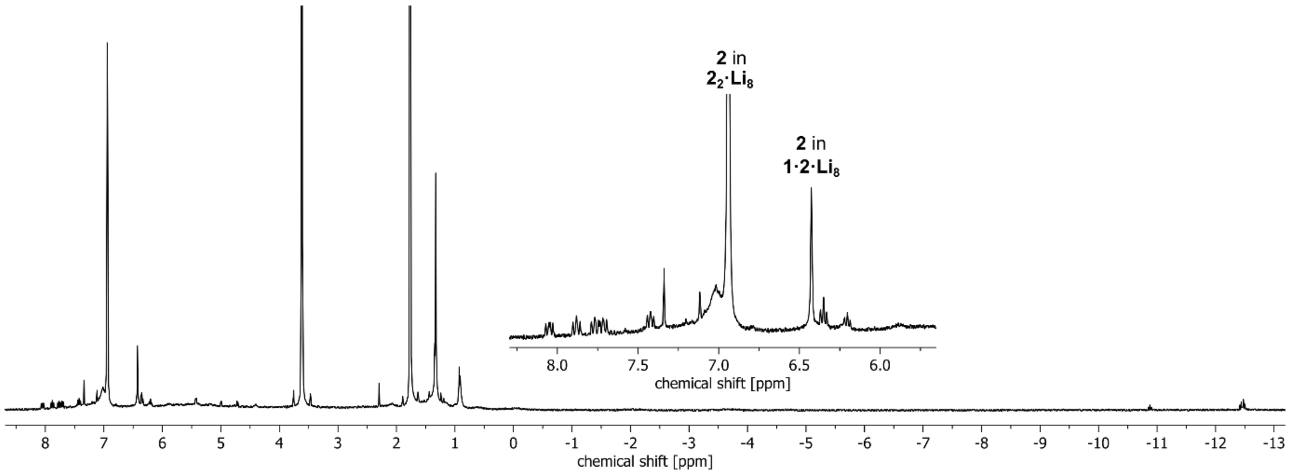

Supplementary Figure 61. ^{1}H NMR spectrum obtained after reducing a 1:2 mixture of **1** and **2**. THF-d_8, 25 °C, 500 MHz. Corannulene signals are assigned to particular sandwiched structures.

Molar ratio **1**:**2** = 2:1

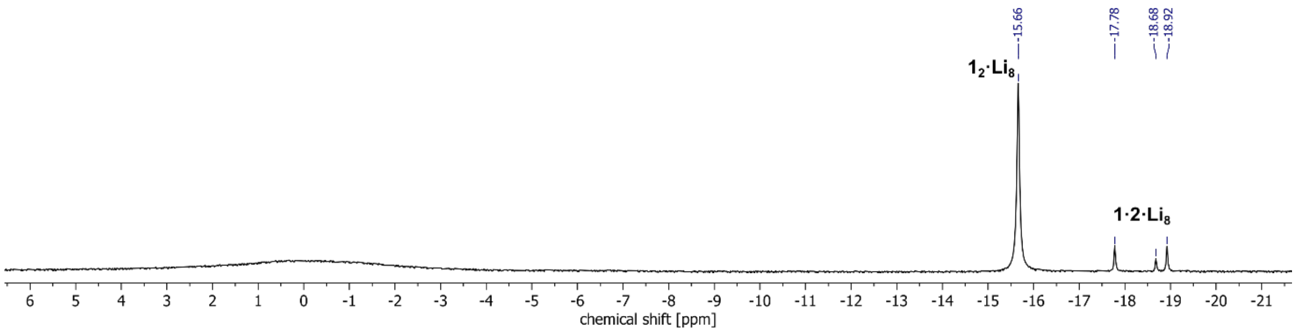

Supplementary Figure 62. ^{7}Li NMR spectrum obtained after in-situ reducing a 2:1 mixture of **1** and **2**. THF-d_8, –60 °C, 194 MHz. Inner Li signals are assigned to particular sandwiched structures.

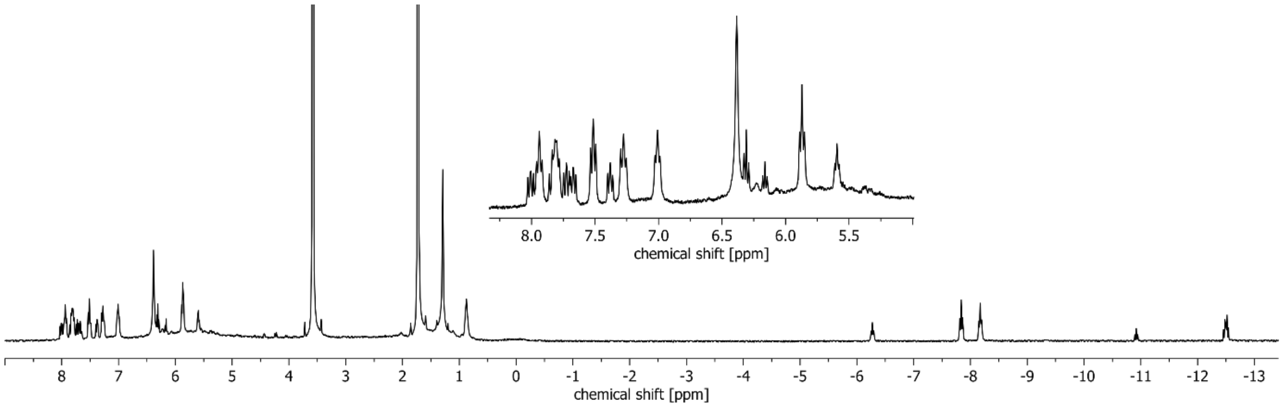

Supplementary Figure 63. ^{1}H NMR spectrum obtained after in-situ reducing a 2:1 mixture of **1** and **2**.

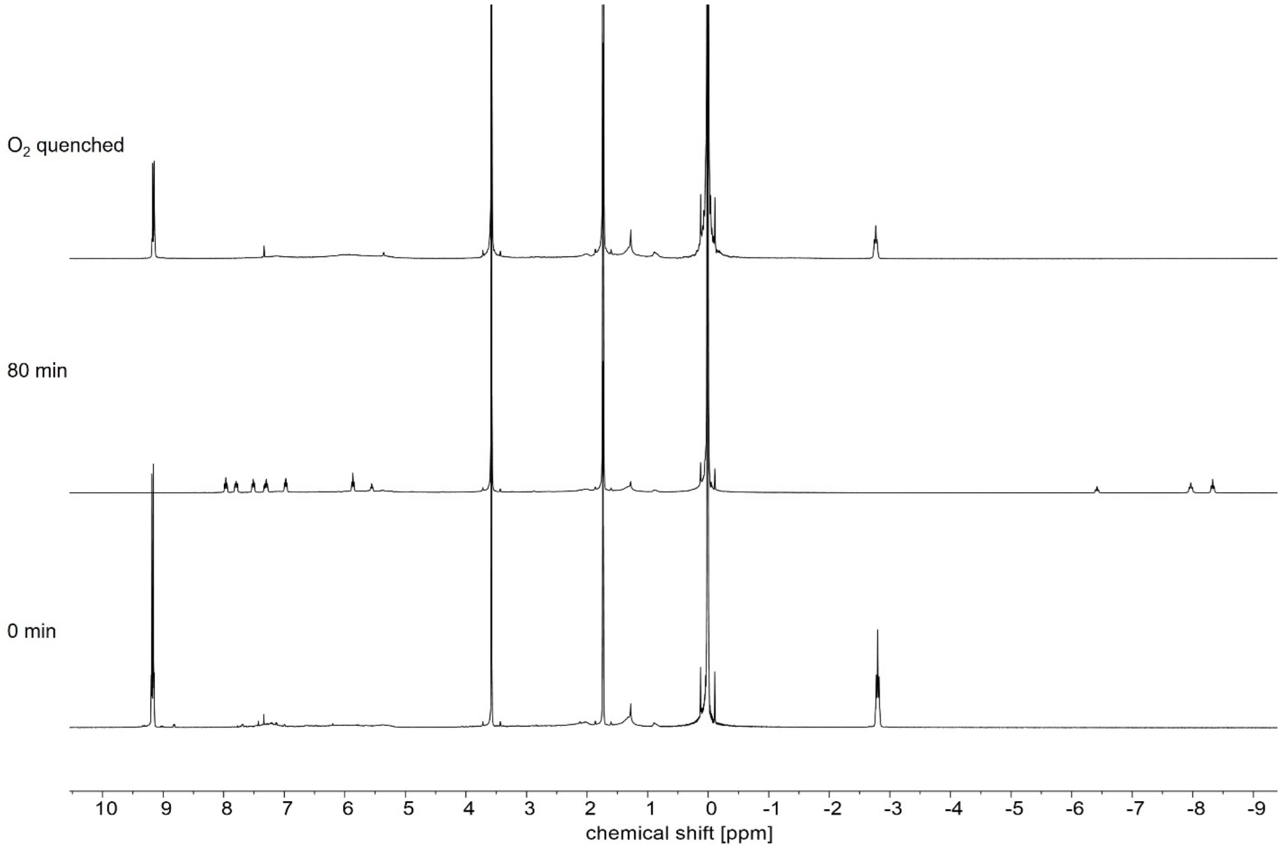

Supplementary Figure 64. ^{1}H NMR demonstrating the reversibility of the reduction of **1** to $\mathbf{1}^{4-}$. Preparation: THF-d_8 (0.70 mL) was added to an NMR tube containing excess Li metal (3.0 mg, 0.528 mmol) and **1** (2.0 mg, 0.0085 mmol). The tube was sealed under argon and ^{1}H NMR spectra were monitored at different reaction times at –40 °C. After stabilizing at the tetra-reduced state, the NMR ampule was exposed to air in order to test the reversibility of the process. All spectra recorded in THF-d_8 at –40 °C, 500 MHz.

6. HOMA and BLA Calculations

HOMA (Harmonic Oscillator Model of Aromaticity) is one of the simplest and most widely used indices for describing aromaticity based on molecular geometry. It uses the C-C bond length in benzene as a standard of perfect aromaticity. The HOMA index can be calculated using the following equation:[14-18]

$$\mathrm{HOMA} = 1 - \frac{\alpha}{n}\sum_{i=1}^{n}(R_i - R_{\mathrm{opt}})^2$$

where R_i and R_{opt} are the ith bond length of the C-C bond in the analyzed ring and the bond length of benzene ring (R_{opt} = 1.388 Å), respectively. n is the number of C-C bonds in the analyzed ring and α = 257.7 Å^{-2} is a normalization factor that gives HOMA value of 1 for perfect aromatic benzene ring and a HOMA value of 0 for an alternating nonaromatic Kekulé cyclohexatriene ring.

The uncertainty (standard deviation) in the HOMA value (σ_{H}) was calculated from the uncertainty of the bond lengths ($\sigma_{\mathrm{R},i}$) using the equation:

$$\sigma_{\mathrm{H}} = \frac{\alpha}{n}\sqrt{\sum_{i=1}^{n}(2\sigma_{\mathrm{R},i}(R_i - R_{\mathrm{opt}})) + \sigma_{\mathrm{R},i}{}^2)^2}$$

Supplementary Table S2. Analysis of HOMA and BLA in lithium salt of [18]annulene tetra-anion $\mathbf{1_2{\cdot}Li_8}$.

For each carbon, BLA is calculated as the absolute value of the difference between the two C-C bonds associated with that atom.

**bond**	**bond length Å**	**uncertainty Å**
C2 → C1	1.39892	0.00313
C3 → C2	1.41786	0.00261
C4 → C3	1.41151	0.00330
C5 → C4	1.40795	0.00295
C6 → C5	1.42017	0.00292
C7 → C6	1.40097	0.00261
C8 → C7	1.40599	0.00300
C9 → C8	1.41614	0.00271
C10 → C9	1.39543	0.00286
C11 → C10	1.39433	0.00270
C12 → C11	1.40782	0.00288
C13 → C12	1.42251	0.00265
C14 → C13	1.39378	0.00289
C15 → C14	1.38949	0.00260
C16 → C15	1.40996	0.00300
C17 → C16	1.41888	0.00270
C18 → C17	1.40568	0.00297
C18 → C1	1.43557	0.00293
	For this ring, HOMA = 0.857 ± 0.009	
	For this ring, BLA = 0.015 ± 0.010 Å<br>(here the uncertainty is the standard deviations of the bond length differences)	
C20 → C19	1.43294	0.00372
C21 → C20	1.40246	0.00348
C22 → C21	1.39822	0.00398
C23 → C22	1.41522	0.00449
C24 → C23	1.41033	0.00362
C25 → C24	1.41338	0.00391
C26 → C25	1.39218	0.00426
C27 → C26	1.41665	0.00404
C28 → C27	1.41579	0.00400
C29 → C28	1.39780	0.00416
C30 → C29	1.42294	0.00419
C31 → C30	1.41639	0.00429
C32 → C31	1.41090	0.00405
C33 → C32	1.40997	0.00390
C34 → C33	1.39173	0.00409
C35 → C34	1.42282	0.00387
C36 → C35	1.41043	0.00387
C36 → C19	1.42642	0.00416
	For this ring, HOMA = 0.826 ± 0.014	
	For this ring, BLA = 0.014 ± 0.010 Å<br>(here the uncertainty is the standard deviations of the bond length differences)	
	Overall average BLA = 0.014 ± 0.010 Å	
	For both rings together, HOMA = 0.841 ± 0.008	

7. Theoretical calculations

7.1. Coulombic repulsion in different conformers of **1**$^{2-}$ was qualitatively evaluated by calculating atomic charges using the Natural Population Analysis[19] in the natural bond orbital (NBO) package supplied with Gaussian16[20]. Results are shown in Fig. S65. In conformer **B**, the most strongly negatively charged atoms (–0.43 |*e*|) are separated by d_{1B} = 7.8 Å, while analogous atoms in **A** are separated by 6.3 Å. The negative charge is also spread more uniformly across the ring in the **B** conformer, with a ~10% smaller mean absolute deviation (MAD_A = 0.099 |*e*|; MAD_B = 0.088 |*e*|).

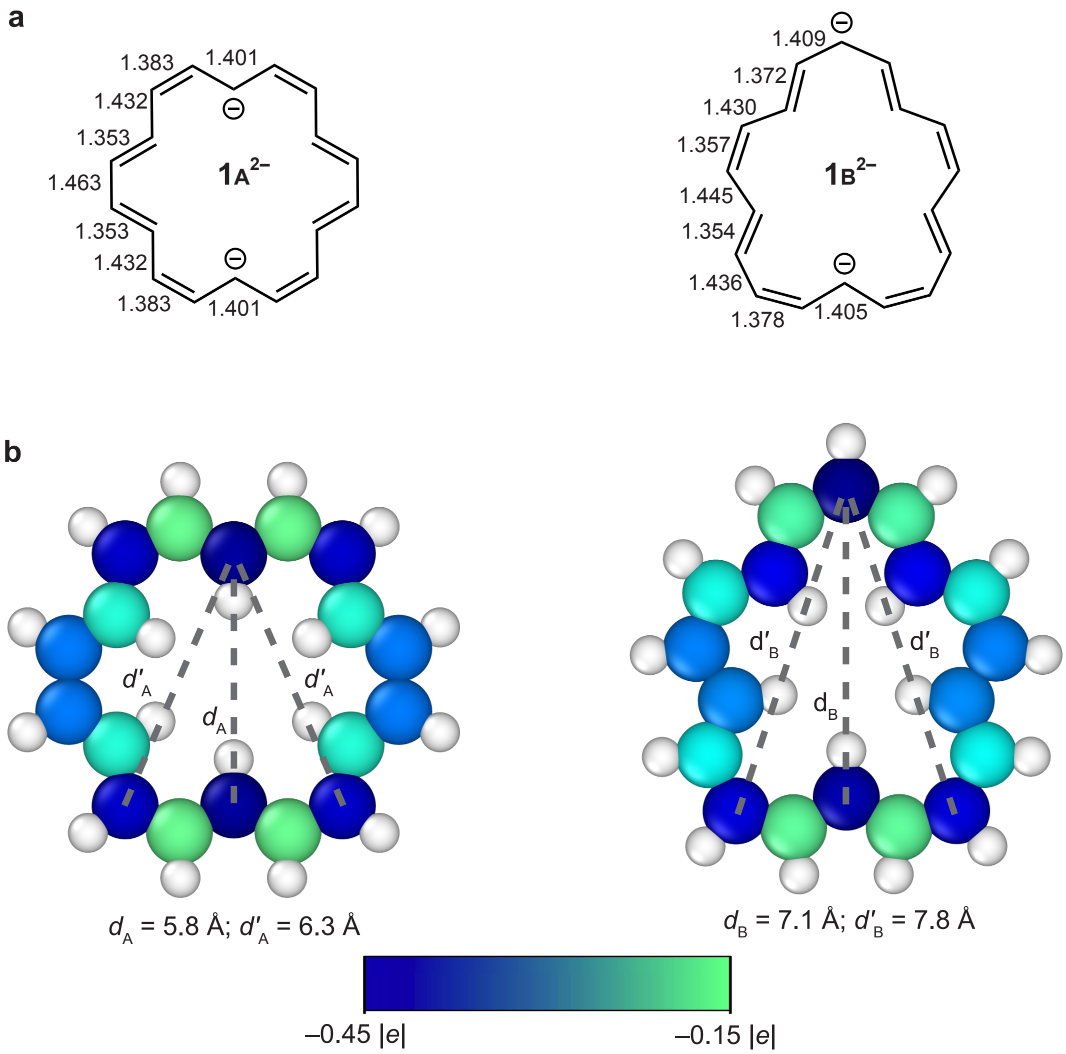

Supplementary Figure 65. Bond lengths (**a**), and NBO charges (**b**) in **1A**$^{2-}$ (left) and **1B**$^{2-}$ (right) calculated at the BLYP45/def2-TZVP level. Dark blue corresponds to –0.45 |*e*|, light green to –0.15 |*e*|. Distances between atoms with the largest negative charge are highlighted.

7.2. Chemical shift calculations were done to evaluate how accurately the chosen functionals describe the electronic structure of **1** and its anions. The experimental difference between the chemical shift of the outer and inner protons was used as reference. For **1** and $\mathbf{1}^{2-}$, best agreement with experiment was obtained in the 40–45% EE range using BLYPxx (Fig. S66), with highly tuned MPWB1K[21] and M06-2X[22] functionals performing worse. This is perhaps not surprising, as the latter two functionals were built for energetics (indeed, M06-2X gives the lowest DLPNO-CCSD(T) energies in all cases), while BHLYP (which serves as a starting point for BLYPxx) was recently found to give excellent magnetizabilities.[23] Due to reasonably good performance for both energies and chemical shifts, BLYP45 was chosen to be the most suitable functional for further orbital and charge analysis. In case of $\mathbf{1}^{4-}$, agreement with experiment was much better for a dimer with six lithium cations (~2 ppm error) than for an isolated molecule (~5 ppm error), as shown in Fig S67.

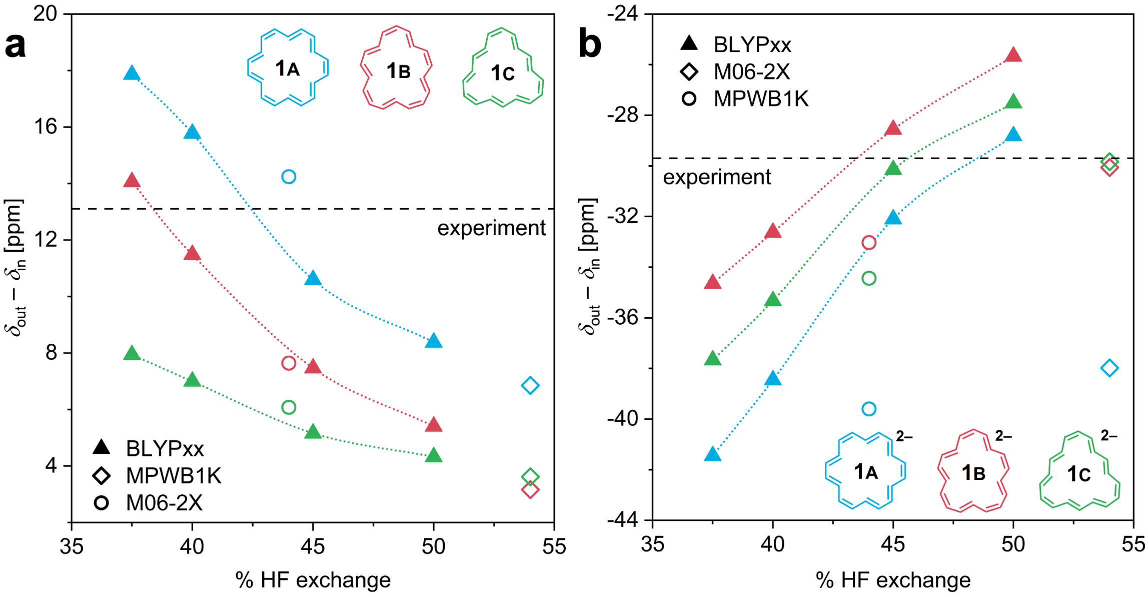

Supplementary Figure 66. Calculated difference between the chemical shift of the outer and the inner protons in (**a**) neutral **1A** (blue), **1B** (red), and **1C** (green) and in their respective anions (**b**), compared to experimental values (dashed line).

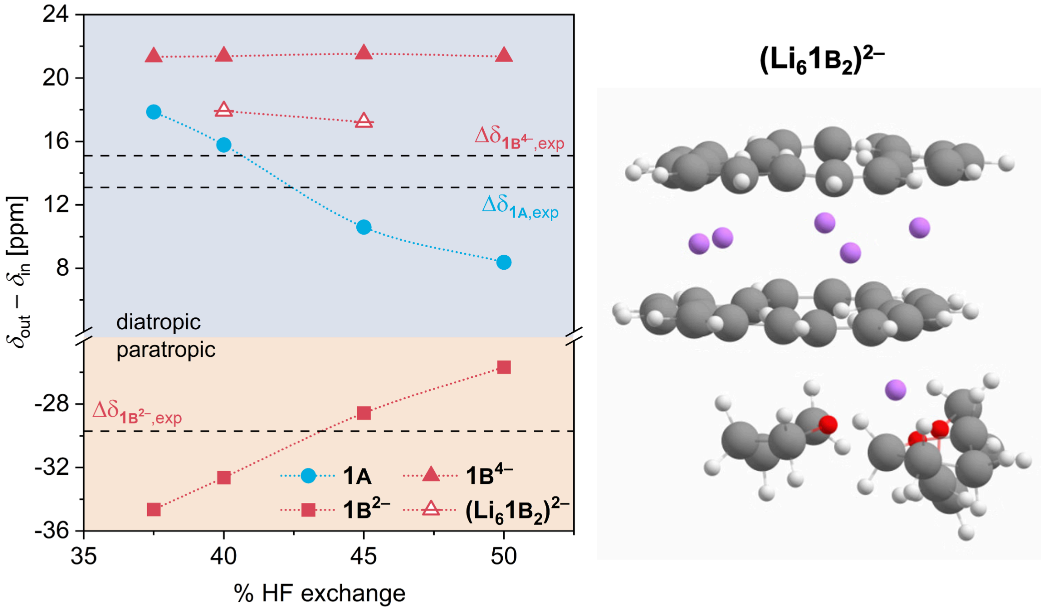

Supplementary Figure 67. Calculated difference between the chemical shift of the outer and the inner protons in conformer **1A** (blue circles), $\mathbf{1B}^{2-}$ (red squares), $\mathbf{1B}^{4-}$ (red triangles) and a $\mathbf{(Li_6 1B_2)^{2-}}$ sandwich with three THF molecules (hollow red triangles, geometry shown on the right), calculated at the BLYPxx level, where xx is the proportion of exact exchange displayed on the horizontal axis. Dashed lines show the experimental values.

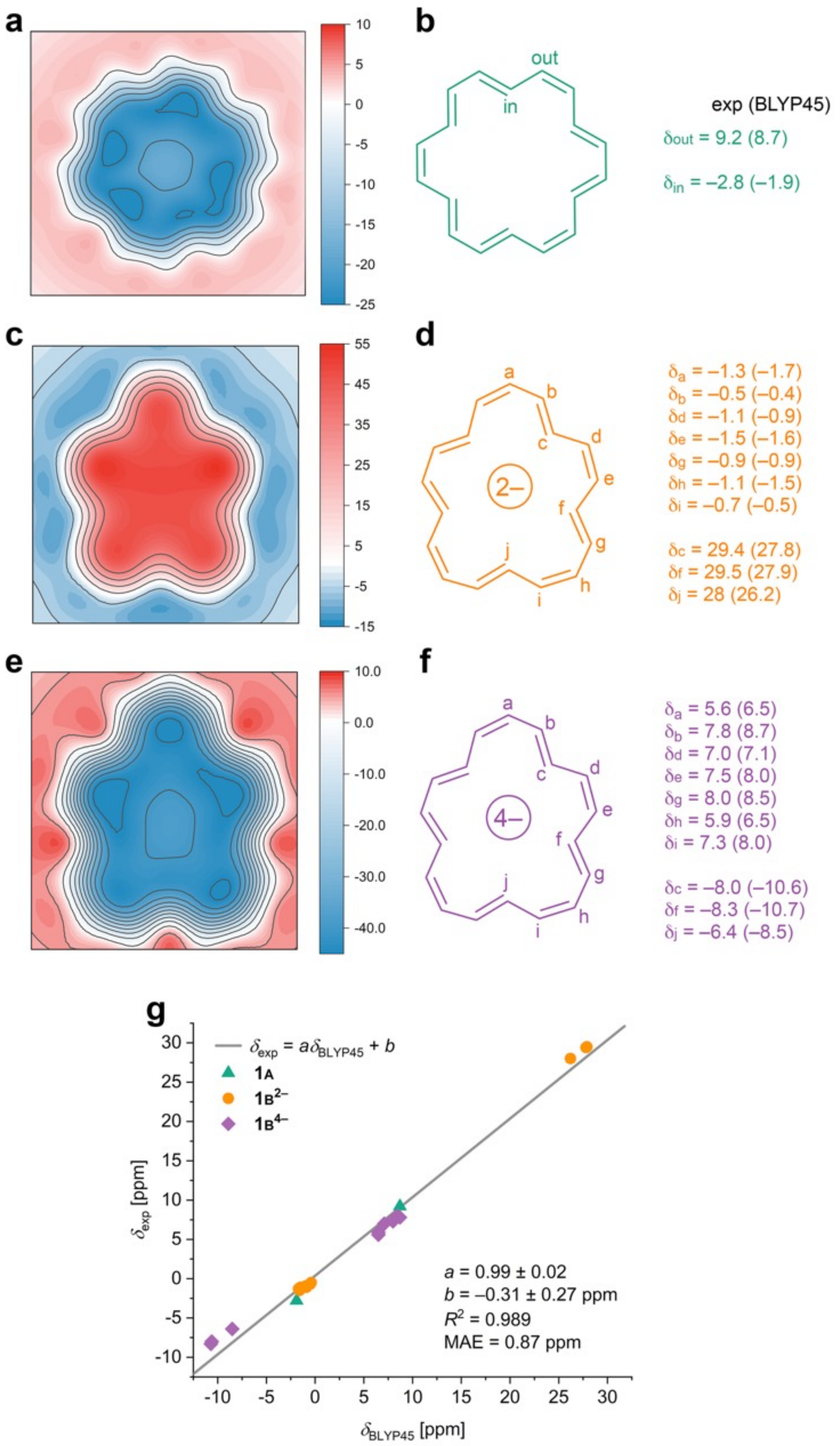

Supplementary Figure 68. NICS$_{zz}$(1.5) values for **1A** (**a**), **1B**$^{2-}$ (**c**), and **1B**$^{4-}$ (**e**). Experimental (and calculated) proton chemical shifts for **1A** (**b**), **1B**$^{2-}$ (**d**; experimental data for K$^+$ salt at –70 °C, Fig. S19; calculated values without counter ions), and **1B**$^{4-}$ (**f**; experimental data for Li$^+$ salt at –40 °C, Fig. S36; calculated values for a **(Li$_6$1b$_2$)**$^{2-}$ sandwich as in Fig. S67, average over both decks). All experimental data were recorded in THF-d_8); **g**, linear fit between the experimental (*y* axis) and calculated (*x* axis) proton chemical shifts **1A** (green triangles), **1B**$^{2-}$ (orange circles), and **1B**$^{4-}$ (purple diamonds), with the slope (*a*), intercept (*b*), coefficient of determination (R^2), and mean absolute error (MAE) shown. All values are computed at BLYP45/def2-TZVP using the chemical shift of benzene in THF-d_8 (7.31 ppm) as a reference.[24]

7.3. Representative input files

All geometry optimizations and DLPNO-CCSD(T*)-F12 calculations were done using ORCA 5.0.4.[25] DFT calculations with the BLYPxx functional used the following input card:

```
# BLYP40 example
! tightopt TightSCF BHLYP RIJCOSX def2-TZVP def2-TZVP/C def2/J

%method
    ACM 0.40, 0.60, 1.0
end

%maxcore 4000

%pal
    nprocs 36
end

* xyzfile 0 1 run.xyz
```

Explicitly correlated[26] local[27] coupled clusters calculations with iterative perturbative triples[28] (DLPNO-CCSD(T*)-F12) were done using the following input card:

```
# ccsdt
! DLPNO-CCSD(T1)-F12D TightPNO RIJCOSX cc-pVDZ-F12 cc-pVDZ-F12-CABS cc-pVTZ/C

%maxcore 9000

%pal
    nprocs 18
end

* xyzfile 0 1 run.xyz
```

Tight thresholds for correlating natural orbitals (*TightPNO* keyword) were shown to be important for a correct description of systems with extended conjugation.[29] Explicitly correlated basis set (*cc-pVDZ-12* and *cc-pVDZ-F12-CABS* keywords) give an advantage of about 2-3 tiers relative to ordinary basis sets[26,30] (i.e. cc-pVDZ-F12 is comparable to cc-pVQZ or cc-pV5Z noncorrelated sets). Including iterative triples (*CCSD(T1)* keyword, denoted as CCSD(T*) in text) was found to improve general performance of the DLPNO-CCSD(T) method, and it was particularly useful for long cumulene chains[28], which are somewhat similar to annulenes.

7.4. Optimized geometries of **1a**, **1b**$^{2-}$, and **1b**$^{4-}$ at BLYP45/def2-TZVP are provided in a separate xyz file.

8. References

[1] Lungerich, D. *et al.* [18]Annulene put into a new perspective. *Chem. Commun.* **52**, 4710–4713 (2016).

[2] Suzuki, M., Comito, A., Khan, S. I. & Rubin, Y. Nanochannel array within a multi-layered network of a planarized dehydro[24]annulene. *Org. Lett* **12**, 2346–2349 (2010).

[3] Spisak, S. N. *et al.* Tuning the separation and coupling of corannulene trianion-radicals through sizable alkali metal belts. *Chem. Sci.* **8**, 3137–3145 (2017).

[4] Kozhemyakina, N. V., Nuss, J. & Jansen, M. Demonstration of the "break-and-seal" approach to fullerides of complex cations at the example of $KC_{60}(THF)_5{\cdot}2THF$ *Z. Anorg. Allg. Chem.*, **635**, 1355–1361 (2009).

[5] SAINT; part of Bruker APEX3 software package (version 2017.3-0): Bruker AXS, 2017.

[6] SADABS; part of Bruker APEX3 software package (version 2017.3-0): Bruker AXS, 2017.

[7] Sheldrick, G. M. SHELXT – Integrated space-group and crystal-structure determination. *Acta Crystallogr.* **A71**, 3–8 (2015).

[8] Sheldrick, G. M. Crystal structure refinement with SHELXL. *Acta Crystallogr.* **C71**, 3–8 (2015).

[9] Dolomanov, O.V. *et al.* OLEX2: A complete structure solution, refinement and analysis program. *J. Appl. Crystallogr.* **42**, 339–341 (2009).

[10] Bregman, J., Hirshfeld, F. L., Rabinovich, D. & Schmidt, G. M. J. The crystal structure of [18]annulene, I. X-ray study. *Acta Cryst.* ***19***, 227–234 (1965).

[11] Hirshfeld, F. L. & Rabinovich, D. The crystal structure of [18]annulene, II. Results. *Acta Cryst.* ***19***, 235–241 (1965).

[12] Gorter, S., Rutten-Keulemans E., Krever, M., Romers, C. & Cruickshank, D. W. J. [18]-Annulene, $C_{18}H_{18}$, structure, disorder and Hückel's 4*n* + 2 rule. *Acta Cryst., Section B: Structural Sci.* **51**, 1036–1045 (1995).

[13] Oth, J. F. M., Woo, E. P. & Sondheimer, F. Unsaturated macrocyclic compounds. LXXXIX. Dianion of [18]annulene. *J. Am. Chem. Soc.* **95**, 7337–7345 (1973)

[14] Kruszewski, J. & Krygowski, T. M. Definition of aromaticity basing on the harmonic oscillator model. *Tetrahedron Lett.* ***13***, 3839–3842 (1972).

[15] Krygowski, T. M. & Cyrański, M. K. Structural aspects of aromaticity. *Chem. Rev.* **101**, 1385–1419 (2001).

[16] Cyrański, M. K. Energetic aspects of cyclic pi-electron delocalization: evaluation of the methods and estimating aromatic stabilization energies. *Chem. Rev.* **105**, 3773–3811 (2005).

[17] Krygowski, T. M., Szatylowicz, H., Stasyuk, O. A., Dominikowska, J. & Palusiak M. Aromaticity from the point of view of molecular geometry: application to the planar systems. *Chem. Rev.* **114**, 6383–6422 (2014).

[18] Makino, M., Nishina, N. & Aihara, J. Critical evaluation of HOMA and MBL as local aromaticity indices. *J. Phys. Org. Chem.* **31**, e3783 (2018).

[19] Reed, A. E., Weinstock, R. B., Weinhold, F. Natural population analysis. *J. Chem. Phys.* **83**, 735–746 (1985).

[20] Gaussian 16, Revision A.03, Frisch, M. J., Trucks, G. W., Schlegel, H. B., Scuseria, G. E., Robb, M. A., Cheeseman, J. R., Scalmani, G., Barone, V., Petersson, G. A., Nakatsuji, H., Li, X., Caricato, M., Marenich, A. V., Bloino, J., Janesko, B. G., Gomperts, R., Mennucci, B., Hratchian, H. P., Ortiz, J. V., Izmaylov, A. F., Sonnenberg, J. L., Williams-Young, D., Ding, F., Lipparini, F., Egidi, F., Goings, J., Peng, B., Petrone, A., Henderson, T., Ranasinghe, D., Zakrzewski, V. G., Gao, J., Rega, N., Zheng, G., Liang, W., Hada, M., Ehara, M., Toyota, K., Fukuda, R., Hasegawa, J., Ishida, M., Nakajima, T., Honda, Y., Kitao, O., Nakai, H., Vreven, T., Throssell, K., Montgomery, J. A., Jr., Peralta, J. E., Ogliaro, F., Bearpark, M. J., Heyd, J. J., Brothers, E. N., Kudin, K. N., Staroverov, V. N., Keith, T. A., Kobayashi, R., Normand, J., Raghavachari, K., Rendell, A. P., Burant, J. C., Iyengar, S. S., Tomasi, J., Cossi, M., Millam, J. M., Klene, M., Adamo, C., Cammi, R.,

Ochterski, J. W., Martin, R. L., Morokuma, K., Farkas, O., Foresman, J. B., Fox, D. J. Gaussian, Inc., Wallingford CT, 2016.

[21] Zhao, Y., Truhlar, D. G. Hybrid meta density functional theory methods for thermochemistry, thermochemical kinetics, and noncovalent interactions: The MPW1B95 and MPWB1K models and comparative assessments for hydrogen bonding and van der Waals interactions. *J. Phys. Chem. A* **108**, 6908–6918 (2004).

[22] Zhao, Y., Truhlar, D. G. The M06 suite of density functionals for main group thermochemistry, thermochemical kinetics, noncovalent interactions, excited states, and transition elements: Two new functionals and systematic testing of four m06-class functionals and 12 other functionals. *Theor Chem Acc* **120**, 215–241 (2008).

[23] Lehtola, S., Dimitrova, M., Fliegl, H., Sundholm, D. Benchmarking magnetizabilities with recent density functionals. *J. Chem. Theory Comput.* **17**, 1457–1468 (2021).

[24] Fulmer, G. R., Miller, A. J. M., Sherden, N. H., Gottlieb, H. E., Nudelman, A., Stoltz, B. M., Bercaw, J. E. & Goldberg, K. I. NMR chemical shifts of trace impurities: Common laboratory solvents, organics, and gases in deuterated solvents relevant to the organometallic chemist. *Organometallics* **29**, 2176–2179, (2010).

[25] Neese, F., Wennmohs, F., Becker, U., Riplinger, C. The ORCA quantum chemistry program package. *J. Chem. Phys.* **152**, 224108 (2020).

[26] Kong, L., Bischoff, F. A., Valeev, E. F. Explicitly correlated R12/F12 methods for electronic structure. *Chem. Rev.* **112**, 75–107 (2012).

[27] Riplinger, C., Pinski, P., Becker, U., Valeev, E. F., Neese, F. Sparse maps—a systematic infrastructure for reduced-scaling electronic structure methods. II. Linear scaling domain based pair natural orbital coupled cluster theory. *J. Chem. Phys.* **144**, 024109 (2016).

[28] Guo, Y., Riplinger, C., Becker, U., Liakos, D. G., Minenkov, Y., Cavallo, L., Neese, F. Communication: An improved linear scaling perturbative triples correction for the domain based local pair-natural orbital based singles and doubles coupled cluster method [DLPNO-CCSD(T)]. *J. Chem. Phys.* **148**, 011101 (2018).

[29] Sylvetsky, N., Banerjee, A., Alonso, M., Martin, J. M. L. Performance of localized coupled cluster methods in a moderately strong correlation regime: Hückel–Möbius interconversions in expanded porphyrins. *J. Chem. Theory Comput.* **16**, 3641–3653 (2020).

[30] Hättig, C., Klopper, W., Köhn, A., Tew, D. P. Explicitly correlated electrons in molecules. *Chem. Rev.* **112**, 4–74 (2012).
